# SIDR: simultaneous isolation and parallel sequencing of genomic DNA and total RNA from single cells

**DOI:** 10.1101/gr.223263.117

**Published:** 2018-01

**Authors:** Kyung Yeon Han, Kyu-Tae Kim, Je-Gun Joung, Dae-Soon Son, Yeon Jeong Kim, Areum Jo, Hyo-Jeong Jeon, Hui-Sung Moon, Chang Eun Yoo, Woosung Chung, Hye Hyeon Eum, Sangmin Kim, Hong Kwan Kim, Jeong Eon Lee, Myung-Ju Ahn, Hae-Ock Lee, Donghyun Park, Woong-Yang Park

**Affiliations:** 1 Samsung Genome Institute, Samsung Medical Center, Seoul 06351, South Korea;; 2 Department of Health Sciences and Technology, SAIHST, Sungkyunkwan University, Seoul 06351, South Korea;; 3 Department of Biomedical Sciences, College of Medicine, Seoul National University, Seoul 03080, South Korea;; 4 Department of Breast Cancer Center, Samsung Medical Center, Seoul 06351, South Korea;; 5 Department of Thoracic and Cardiovascular Surgery, Samsung Medical Center, Seoul 06351, South Korea;; 6 Department of Surgery, Samsung Medical Center, Sungkyunkwan University School of Medicine, Seoul, 06351, South Korea;; 7 Division of Hematology-Oncology, Department of Medicine, Samsung Medical Center, Sungkyunkwan University School of Medicine, Seoul 06351, South Korea;; 8 Department of Molecular Cell Biology, Sungkyunkwan University School of Medicine, Suwon 16419, South Korea

## Abstract

Simultaneous sequencing of the genome and transcriptome at the single-cell level is a powerful tool for characterizing genomic and transcriptomic variation and revealing correlative relationships. However, it remains technically challenging to analyze both the genome and transcriptome in the same cell. Here, we report a novel method for simultaneous isolation of genomic DNA and total RNA (SIDR) from single cells, achieving high recovery rates with minimal cross-contamination, as is crucial for accurate description and integration of the single-cell genome and transcriptome. For reliable and efficient separation of genomic DNA and total RNA from single cells, the method uses hypotonic lysis to preserve nuclear lamina integrity and subsequently captures the cell lysate using antibody-conjugated magnetic microbeads. Evaluating the performance of this method using real-time PCR demonstrated that it efficiently recovered genomic DNA and total RNA. Thorough data quality assessments showed that DNA and RNA simultaneously fractionated by the SIDR method were suitable for genome and transcriptome sequencing analysis at the single-cell level. The integration of single-cell genome and transcriptome sequencing by SIDR (SIDR-seq) showed that genetic alterations, such as copy-number and single-nucleotide variations, were more accurately captured by single-cell SIDR-seq compared with conventional single-cell RNA-seq, although copy-number variations positively correlated with the corresponding gene expression levels. These results suggest that SIDR-seq is potentially a powerful tool to reveal genetic heterogeneity and phenotypic information inferred from gene expression patterns at the single-cell level.

As cell-to-cell variability has come to be recognized as fundamental to a variety of biological processes, there has been a demand for high-throughput analysis technologies that would allow quantification of a large number of parameters in a single cell. In particular, recent improvements in sequencing technology have led to the advancement of genome-wide quantitative analysis of single cells. Although intercellular genetic heterogeneity in a population of cells has been frequently ignored in genome analyses at the population level, there is increasing evidence of unexpectedly high genetic variability in cell populations within an organism (Shapiro et al. 2013; Junker and van Oudenaarden 2014). Along with other technological advances, single-cell genome sequencing has become crucial for characterizing intercellular genetic heterogeneity and thus cell-lineage relationships (Dey et al. 2015; Macaulay et al. 2015). Examples of intercellular genetic heterogeneity are found in every tissue in the human body under normal physiological conditions, including the immune system, as well as cells under pathological conditions, such as cancer cells.

Although genomic differences are arguably the most fundamental source of cellular variability, stochastic gene expression processes cause intercellular heterogeneity even within a genetically homogenous population. To uncover cell-to-cell variability in gene expression, single-cell RNA-seq (scRNA-seq) utilizing massively parallel sequencing has emerged as the preferred method for providing a full overview of the expression of all genes, overtaking other assays analyzing only a handful of genes at a time. In fact, a number of different scRNA-seq methods have been developed, including Smart-Seq (Ramsköld et al. 2012), STRT-seq (Islam et al. 2012), CEL-Seq (Hashimshony et al. 2012), MARS-Seq (Jaitin et al. 2014), and Quartz-Seq (Sasagawa et al. 2013). These technologies measuring genome-wide mRNA expression at the single-cell level are being utilized to uncover distinct cell types, states, and circuits within cell populations and tissues. After profiling genome-wide mRNA expression of single cells in a plethora of cell populations, it is clear that “seemingly homogeneous” cells are in fact heterogeneous.

Until recently, the effects of genomic variation on phenotypic expression profiles have been primarily studied at the population level (Stranger et al. 2007; Shapiro et al. 2013; Junker and van Oudenaarden 2014). Since the genomic and transcriptomic profiles obtained from pooling thousands to millions of cells represent averaged information of a large population, these conventional methods are inadequate to reflect the typical variability among individual single cells (Shapiro et al. 2013; Junker and van Oudenaarden 2014). Consequently, given the complexity of gene expression regulation and significant cell-to-cell heterogeneity, unveiling the causal relationships between genomic variations and mRNA transcription profiles turned out to be very challenging (Altschuler and Wu 2010; Han et al. 2014). Thus, there is a growing demand to integrate DNA and RNA analyses to study genotype–phenotype associations within single cells, which allows a more accurate assessment of the correlation between genotypes and gene expression levels (Shapiro et al. 2013; Junker and van Oudenaarden 2014).

Although substantial progress has been made in recent years in single-cell analysis technologies, many challenges remain in the simultaneous analysis of genome and transcriptome data from the same cell (Han et al. 2014; Dey et al. 2015). The limited choices of amplification methods, inherent losses of nucleic acids arising from separation methods, and restrictive profiling for genome-wide regions still need to be overcome (Dey et al. 2015; Macaulay et al. 2015; Hou et al. 2016).

Here, we report a simple, yet efficient method for the simultaneous isolation of genomic DNA and total RNA (SIDR) from single cells. The method physically isolates total RNA, regardless of polyadenylation, from the single-cell lysate that contains the nucleus by using magnetic microbead capture.

## Results

### Development of the SIDR method for simultaneously isolating genomic DNA and total RNA from single cells

**Figure 1.**
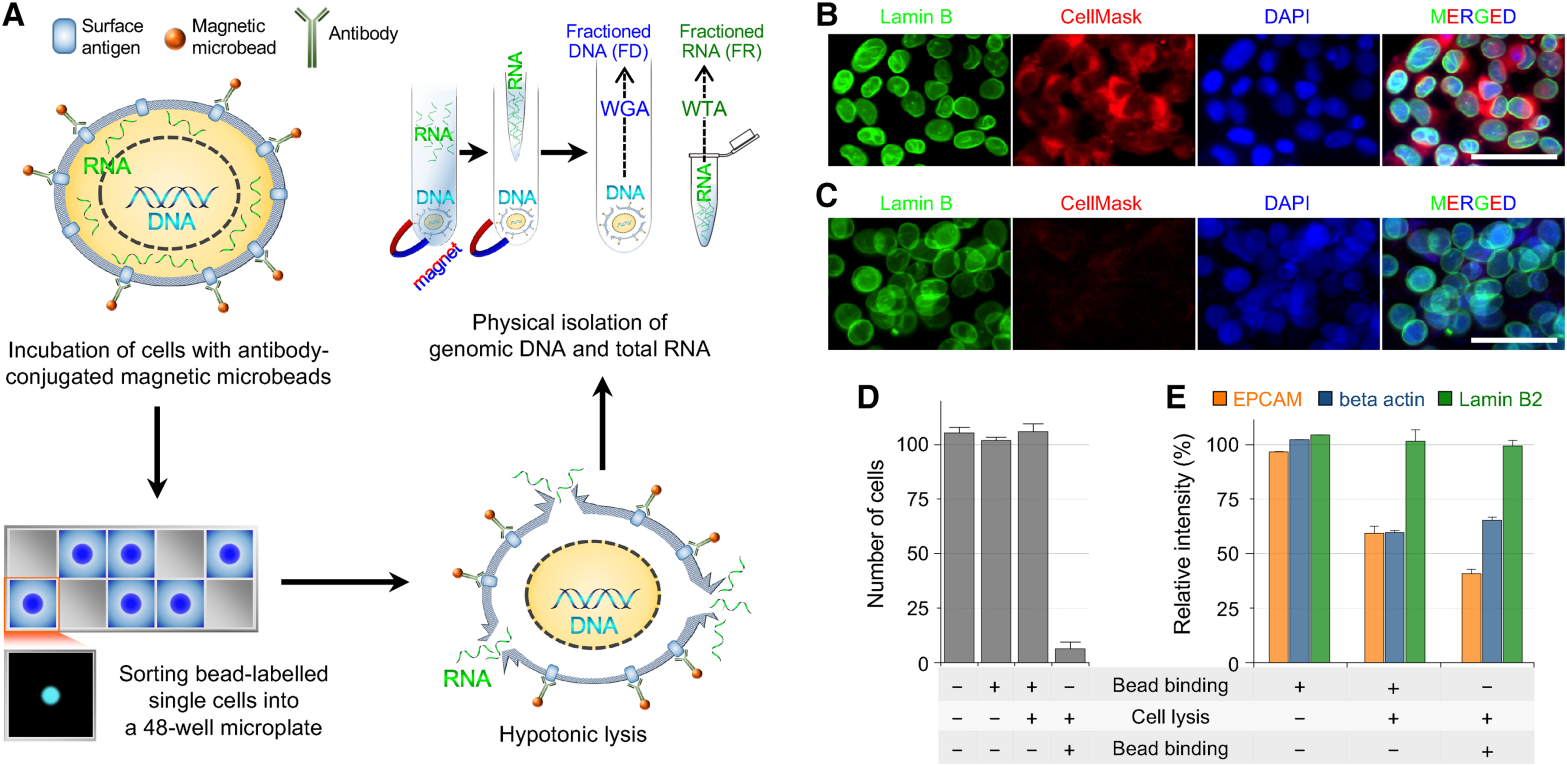
Principles of the SIDR method. (*A*) Schematic of the SIDR method. (WGA) whole-genome amplification; (WTA) whole transcriptome amplification. (*B*,*C*) Immunostaining of the nucleus after cell lysis. Fluorescence images of MCF7 cells in isotonic (*B*) and hypotonic (*C*) conditions. The nuclear lamina, plasma membrane, and nucleus were stained by the Alexa 488-labeled anti-Lamin B2 antibody (green), CellMask (red), and DAPI (blue), respectively. (*D*) The effect of cell lysis on the recovery rate of cells. The recovery rate dramatically depended on whether anti-EPCAM antibody-conjugated microbeads were bound to cells before or after cell lysis as indicated at the *bottom* of the graph. Approximately 100 MCF7 cells underwent bead binding and/or cell lysis and were magnetically recovered, except for control cells that were not bound to microbeads. The plot shows the number of cells recovered (*n* = 3). (*E*) The effect of bead binding on the solubilization of the EPCAM protein. The levels of EPCAM, beta actin, and Lamin B2 proteins in cell lysates not solubilized during cell lysis were measured by Western blot (*n* = 3).

First, we aimed to establish a lysis condition that would allow efficient diffusion of RNA, but not of DNA, out from a lysed cell (Fig. 1A). We examined hypotonic lysis methods, because osmotic pressure can efficiently disrupt the plasma membrane to release cytoplasmic components, whereas the integrity of the nuclear membrane would be maintained because of the presence of nuclear pores. Indeed, we found that a hypotonic solution containing 0.2% Triton X-100, a mild nonionic detergent, efficiently lysed the cell membrane to release cytoplasmic RNA, whereas genomic DNA remained within the nucleus. The nuclear lamina visualized with an anti-Lamin B2 antibody was well-preserved despite slight swelling (Fig. 1B,C). Moreover, genomic DNA visualized by DAPI staining was predominantly confined within the nucleus (Fig. 1C).

To separate the supernatant containing total RNA from the cell lysate, we attempted to coat the latter with antibody-conjugated magnetic microbeads (Fig. 1A). A critical problem was that the detergent included in the hypotonic solution for efficient RNA release extracted the cell surface–associated proteins and cellular organelles (Borner et al. 1994; Koley and Bard 2010). In fact, none of the antibodies we tested could successfully label the lysed cells. Thus, we coated cells with antibody-conjugated magnetic microbeads before cell lysis and then examined whether the microbeads remained associated with the cells after cell lysis. The association of microbeads with the cells after lysis was estimated by measuring the capturing efficiency of cells. We initially tested several antibodies, produced by different vendors, which targeted nuclear membrane proteins or plasma membrane proteins, because the association between beads and cells primarily depends on the interaction between the antibody and its target antigen. When an anti-EPCAM antibody was used, cells bound to microbeads prior to hypotonic cell lysis were efficiently recovered after cell lysis (Fig. 1D).

To elucidate how the change in the procedure (i.e., bead binding after versus before cell lysis) resulted in such a dramatic difference in the recovery yield, we examined whether magnetic beads bound to the plasma membrane could hinder the extraction of the EPCAM protein from the membrane during cell lysis. We quantified the amount of EPCAM protein present before and after cell lysis and investigated how these quantities were modified by prebinding of microbeads. Figure 1E shows that without prebinding of microbeads, the surface EPCAM protein levels decreased to 40.6% after cell lysis compared to the levels detected in the isotonic condition. On the other hand, bead binding prior to cell lysis attenuated the decrease in the amount of surface EPCAM protein after cell lysis to ∼60%, suggesting that the interaction of EPCAM with the antibody-conjugated bead surface prevented EPCAM protein from being solubilized. This allowed us to separate the supernatant (total RNA fraction) from the bead-bound cell lysate (genomic DNA fraction). In contrast to the decline in EPCAM level after cell lysis, the level of the lamin B2 protein measured by Western blotting was well-preserved under the same condition, which was consistent with the results of the lamin B2 immunostaining experiments.

Based on these results, we developed the SIDR method, which consisted of four steps (Fig. 1A): (1) incubation of dissociated cells with the antibody-conjugated magnetic microbeads; (2) sorting of microbead-labeled single cells into a 48-well microplate; (3) hypotonic lysis of bead-labeled single cells; and (4) separation of the supernatant containing total RNA from the nucleus-containing cell lysate using magnetic force. The experimental protocols for the method are available in the Supplemental Methods.

### Highly efficient recovery of DNA and RNA by SIDR

Next, we examined the recovery yields of genomic DNA and total RNA by the SIDR method. For accurate measurements based on real-time PCR, we used 10 dissociated cells instead of single cells. We then obtained preparations of fractionated genomic DNA and total RNA (FD and FR, respectively) by the SIDR method. Whole-cell lysates containing genomic DNA and total RNA (WD and WR, respectively) were used as control preparations for the comparison of nucleic acid concentrations obtained by these methods.

**Figure 2.**
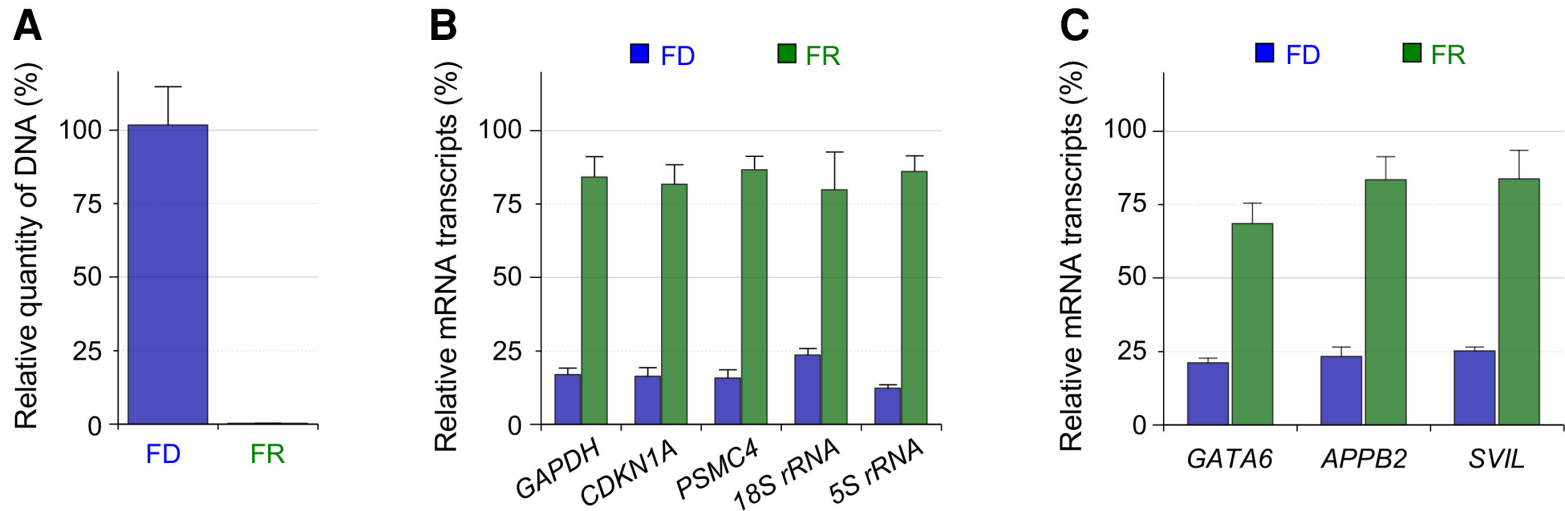
Recovery rates of DNA (*A*) and RNA (*B*,*C*) by the SIDR method. (*A*) The efficiency of DNA recovery by the SIDR method was estimated by real-time PCR targeting the LINE-1 locus. (*B*) The efficiency of cytoplasmic RNA recovery by the SIDR method was estimated by real-time PCR targeting *GAPDH*, *CDKN1A*, *PSMC4*, 18S rRNA, and 5S rRNA. (*C*) The additional three transcripts reported to be enriched in the nucleus were assessed by real-time PCR targeting *GATA6*, *APBB2*, and *SVIL*. Nucleic acids were extracted from 10 MCF7 cells. FD and FR refer to genomic DNA and total RNA, respectively, fractionated by the SIDR method. The amount of DNA in FR and of RNA in FD indicates the amount of residual contamination in the counterpart fractions due to incomplete separation. The amounts of nucleic acids in each fraction were normalized to those in the whole-cell lysates of 10 MCF7 cells. Error bars represent the SEM.

For accurate measurement of small amounts of genomic DNA, we took advantage of a repetitive sequence in the human genome. Since the long interspersed nuclear element-1 (LINE-1) constitutes ∼17% of the human genome (up to 600,000 copies), we used real-time PCR targeting the LINE-1 locus to quantify genomic DNA (Phokaew et al. 2008). At first, MCF7 cell lines were tested to validate the recovery rate of DNA and RNA by SIDR. As shown in Figure 2A, the relative quantity of genomic DNA in FD (C_p_ ≅ 23.6 ± 0.280) was similar to that in WD (C_p_ ≅ 23.7 ± 0.245). In addition, the amount of genomic DNA in FR was minimal (C_p_ ≅ 31.2 ± 1.037), indicating that the leakage of genomic DNA into the supernatant was negligible during cell lysis (Fig. 2A).

In parallel, the recovery of RNA from 10 MCF7 cells by the SIDR method was also assessed. FR and FD obtained by SIDR were reverse transcribed to synthesize cDNA. The amount of RNA in FR and FD was measured to estimate the RNA recovery yield and the level of RNA contamination in the DNA fraction. We performed RT-qPCR to analyze the relative RNA amount of three different genes: *GAPDH* (glyceraldehyde 3-phosphate dehydrogenase), *CDKN1A* (cyclin-dependent kinase inhibitor 1), and *PSMC4* (proteasome 26S subunit, ATPase 4). The SIDR method resulted in negligible contamination of the DNA fraction by RNA, whereas fractions of recovered RNA molecules encoded by the three genes were high: 84.1% for *GAPDH*, 81.7% for *CDKN1A*, and 86.6% for *PSMC4* (Fig. 2B). Although the recovery yields of individual transcripts may vary depending on the target genes, ∼80% of RNA molecules were recovered by the SIDR method. This high RNA recovery rate was also supported by measuring highly abundant ribosomal RNA, which showed values of 79.8% and 86.0% for 18S rRNA and 5S rRNA, respectively (Fig. 2B).

We also evaluated the recovery rates of DNA and RNA of the SIDR method in different cell lines, such as HCC827 and SKBR3 (Supplemental Fig. S1), which showed results consistent with those from MCF7. Furthermore, to investigate whether the SIDR method was sufficiently robust to efficiently isolate DNA and RNA from different tissue samples, we dissociated cells from breast cancer and lung cancer tissues from patients (*n* = 5) and applied the SIDR method to the dissociated cancer cells. Based on the RT-qPCR assays applied for MCF7 cells, the SIDR method recovered most genomic DNA and ∼70%−80% of RNA, which was comparable to the results from MCF7 cells (Supplemental Fig. S2).

To examine whether nuclear-enriched mRNAs remained trapped in the nucleus or cell lysate after the hypotonic lysis process, we selected three transcripts reported to be enriched in nucleus from a previous study: GATA binding protein 6 (*GATA6*), amyloid beta A4 precursor protein-binding family B member 2 (*APBB2*), and Supervillin (*SVIL*) (Barthelson et al. 2007). The relative RNA amounts in FR and FD were analyzed by RT-qPCR, and the SIDR method recovered ∼70% of these transcripts in the three cell lines (Fig. 2C; Supplemental Fig. S1C,F) and tissue samples (Supplemental Fig. S2C,F). These results indicate that nuclear-enriched mRNAs were efficiently released into FR during the SIDR process (Fig. 2C).

Taken together, our data demonstrated that the SIDR method efficiently isolated both genomic DNA and total RNA without significant cross-contamination.

### Sample preparation and data generation for single-cell sequencing by SIDR

To perform single-cell sequencing of DNA and RNA fractions obtained by SIDR (i.e. scSIDR-seq), we used two breast cancer cell lines (MCF7 and SKBR3) and a lung cancer cell line (HCC827). A total of 43 pairs of FD and FR SIDR preparations from single cells were used to construct sequencing libraries. In addition, 30 WDs and 40 WRs of single cells were processed as control preparations. Among the 43 single cells processed by the SIDR method, 38 (88.4%) single cells passed quality-control criteria for library preparation of both RNA and genomic DNA (Supplemental Table S1). We performed low-coverage whole-genome sequencing (WGS; mean coverage ranging from 0.13 to 0.79×) for 68 single cells (38 FDs and 30 WDs) and RNA sequencing (RNA-seq) for 74 single cells (38 FRs and 36 WRs). WGS data generated from FDs and WDs passed sequencing quality control at rates of 81.6% and 90.0%, respectively (Supplemental Table S1; Supplemental Fig. S3). Among the 74 scRNA-seq samples, 37 FRs (97.4%) and 33 WRs (91.7%) generated qualifying RNA-seq data (Supplemental Table S1).

### Single-cell genome sequencing by SIDR-seq

**Figure 3.**
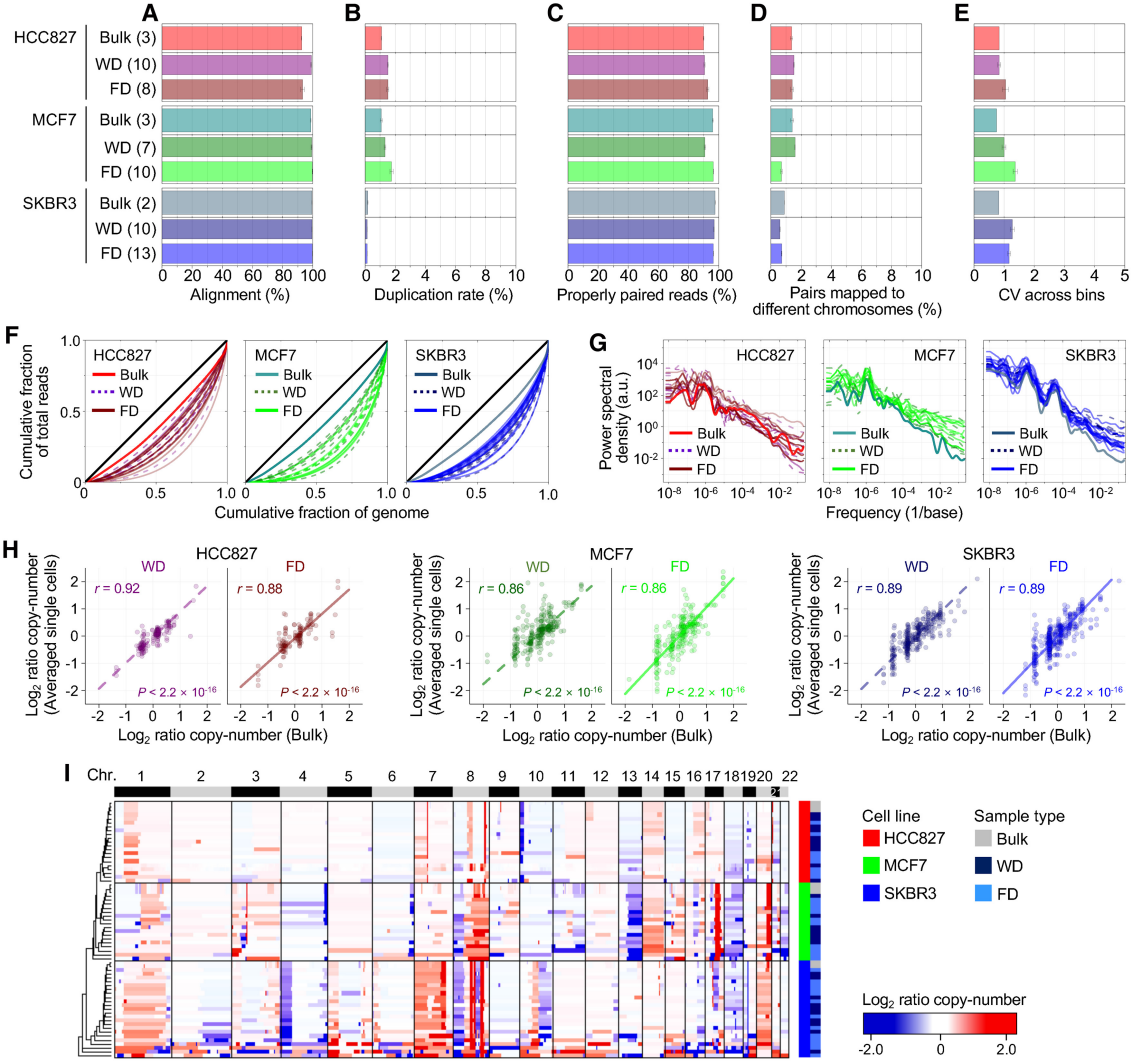
Evaluation of single-cell WGA performance using SIDR-seq. (*A–D*) Summary of sequencing metrics. The number of samples is indicated in parentheses. The plots display fractions of sequencing reads properly aligned to the human reference genome (*A*), duplicated (*B*), properly paired (*C*), and with their paired reads mapped to different chromosomes (*D*). (*E*) Bin-to-bin variability in genomic DNA read counts. (*F*) Lorenz curves illustrating the relationship between the cumulative fractions of the genome covered (*x*-axis) and those of mapped bases (*y*-axis). The diagonal black lines indicate theoretical perfect uniformity. (*G*) Power spectrum of read distributions over different genomic length scales. (*H*) Correlations of copy numbers between bulk cells and averaged single cells. Pearson's correlation coefficients (*r*) with their statistical significances (*P*) are shown. (*I*) Unsupervised clustering heatmap of genome-wide copy-number profiles in bulk and single cells from HCC827, MCF7, and SKBR3 cells. The dendrogram was generated based on the Euclidean distance metric with Ward's method (Ward 1963). (FD) DNA fractionated from single cells by SIDR; (WD) genomic DNA from the whole-cell lysates of single cells.

We applied scSIDR-seq to sequence whole genomes of single cells at a low depth of coverage (0.32 ± 0.02× [mean ± SEM]) and examined whether WGS data generated from FDs were of similar quality in several respects to those from WDs (Fig. 3). After processing sequencing data, sequencing metrics such as the duplicate rate and the fractions of properly aligned and paired reads indicated comparable data quality between FD and WD (Fig. 3A–D). Additionally, FD data from scSIDR-seq showed similar coefficients of variation across the data bins as those seen in bulk cells or WD (Fig. 3E). We used Lorenz curves to evaluate coverage uniformity along the genome, which shows the cumulative fraction of the total reads that cover a given cumulative fraction of the genome. When we compared the Lorenz curves for FD and WD, there was no significant difference between the two groups, indicating a similar level of coverage uniformity and sequencing biases (Fig. 3F). We also plotted the power spectrum of read density variation to show the spatial scale at which any variations might occur between the groups. The power spectrum analysis indicated a similar level of coverage uniformity between FDs and WDs across the entire range of the spatial scale (Fig. 3G).

We next profiled copy numbers across the genome in each sample and averaged the copy-number values from single cells for each group. Across the genome, the average copy-number values from the two groups were highly correlated with those from bulk cells (Fig. 3H; Supplemental Fig. S4A). When we performed unsupervised hierarchical cluster analysis based on the copy-number profiles, samples from the same cell lines were clustered together regardless of the sample type, indicating accurate detection of copy-number variation by scSIDR-seq (Fig. 3I; Supplemental Fig. S4B). This was further supported by our observation that, in a pairwise comparison with bulk samples, copy numbers of FD had similar Pearson correlation coefficients to those of WD (Supplemental Fig. S4B). Furthermore, when we detected single-nucleotide variations (SNVs) in single-cell WGS data, the frequencies at which the SNVs were also found in the corresponding bulk data were not significantly different between FD and WD (Supplemental Fig. S5). These results strongly suggest that FD isolated by SIDR is satisfactory for single-cell DNA sequencing.

### Single-cell transcriptome sequencing by SIDR-seq

**Figure 4.**
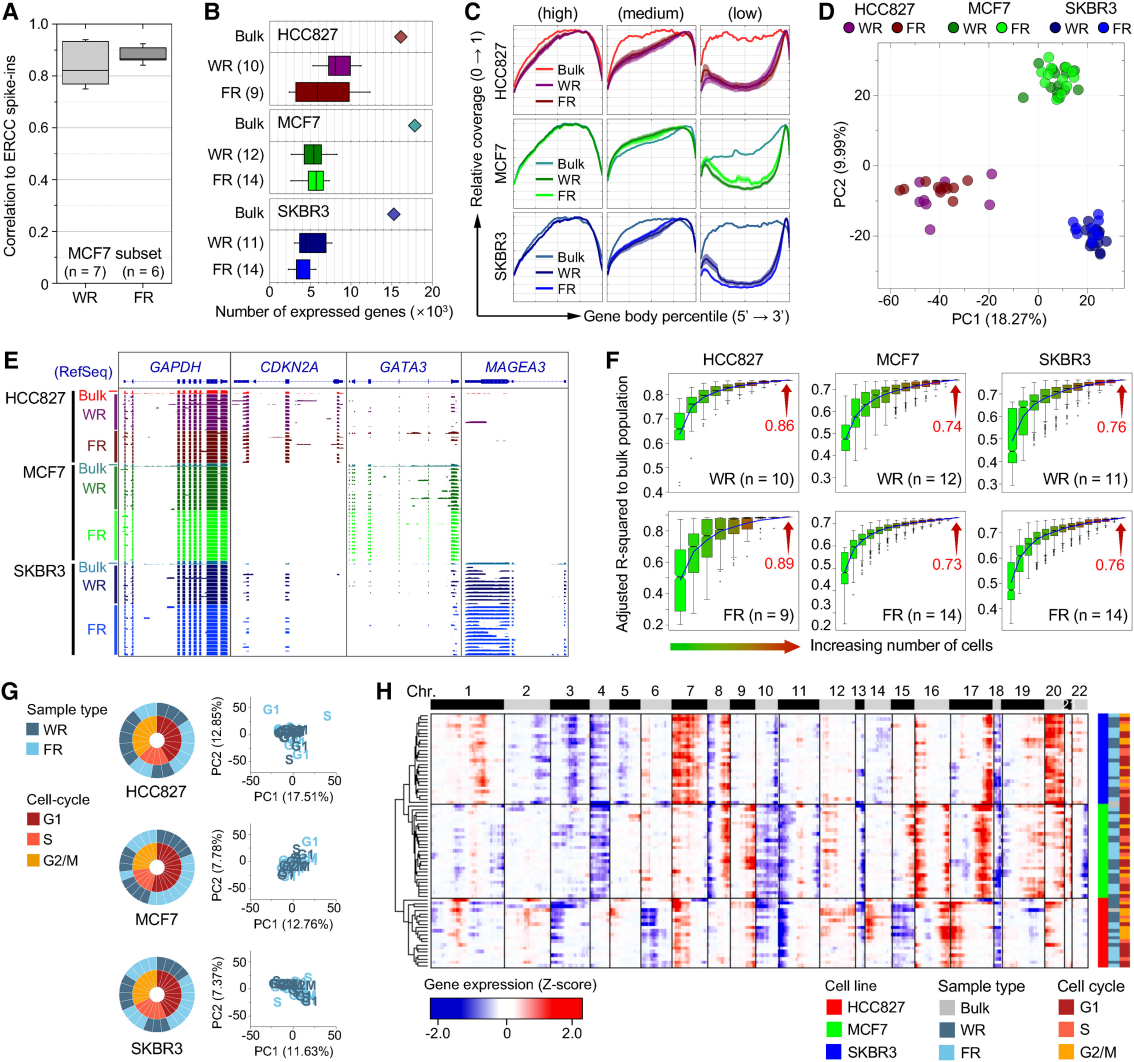
Evaluation of single-cell RNA-seq performance using SIDR-seq. (*A*) Correlations of ERCC spike-in standards between RNA input abundance and read density output for WR and FR. The box plot shows the distribution of the correlation coefficients for the detected synthetic RNAs. (*B*) Number of genes detected in single-cell WRs and FRs. For HCC827, MCF7, and SKBR3 cell lines, bulk data are displayed on *top* of corresponding cell lines as a reference. The number of samples is indicated in parentheses. (*C*) Sequence coverage along the normalized transcript length. For the analysis, transcripts were selected based on their expression level. Transcripts were rank-ordered by expression level and classified into three categories in each sample: top 1000 transcripts (*left*), middle 1000 transcripts (*middle*), and bottom 1000 transcripts (*right*). Coverage ratio was normalized to the maximal degree of coverage in each sample. (*D*) Principal component analysis (PCA) of HCC827, MCF7, and SKBR3 single-cell transcriptomes. Genes used in PCA are identified in Supplemental Figure S11A. RNA-seq data of both WRs and FRs single cells from each cell line clustered together. (*E*) Genomic snapshots of cDNA read alignments in the *GAPDH*, *CDKN2A*, *GATA3*, and *MAGEA3* genes. (*F*) Adjusted *R*^2^ of gene expression in various numbers of single cells relative to the bulk cells was determined by multiple regression analysis with randomly selected cell numbers (with permutation ×1000). For the box plots in *B* and *F*, the box indicates interquartile range (IQR) between the first and the third quartiles, and the error bar shows 10–90th percentiles. (*G*) PCA of single cells subgrouped based on the cell-cycle stages. Gene sets used in PCA were identified in Supplemental Figure S13A. The cell-cycle stage of each single cell was determined by the expression of 874 cell-cycle marker genes (Whitfield et al. 2002), as shown in Supplemental Figure S12D. The overall fractions of cell-cycle phases of WRs and FRs were displayed on the *left* side. (*H*) Unsupervised hierarchical clustering heatmap of chromosomal gene expression patterns. The dendrogram was generated based on the Euclidean distance metric with Ward's method (Ward 1963). (FR) RNA fractionated by SIDR from single cells; (WR) total RNA from whole-cell lysates of single cells.

Before comparing RNA-seq data between single-cell FRs and single-cell WRs, we only retained samples in which the eight genes that were least variably expressed at high levels fit the quality-control criteria as described in the Supplemental Methods for further downstream analyses (Supplemental Figs. S6–S9). To account for technical noise in scRNA-seq as described in Brennecke et al. (2013), equal amounts of External RNA Controls Consortium (ERCC) spike-ins were added into 14 MCF7 single-cell samples (six FRs and eight WRs). Of these samples, RNA-seq data from six FRs and seven WRs satisfied quality-control criteria. The amounts of ERCC spike-in detected in both FR and WR samples were strongly correlated with the initial input molecules across all the ERCC references (Fig. 4A). In transcriptome analysis of 70 single cells (WR 33; FR 37), we detected 2400–11,000 genes per cell, without a discernible difference in the detected gene numbers between the FR group (5209 ± 2247) and the WR group (6172 ± 2178) (Fig. 4B; Supplemental Fig. S10). When ensemble data sets were constructed by pooling raw reads from all single-cell data for each group and randomly subsampled to a given total read count, the numbers of genes detected in the ensembles were also comparable in the two groups (Supplemental Fig. S10). We also examined the depth of coverage depending on the relative position of transcripts because the sequencing bias toward the 3′ ends of transcripts is known to increase among smaller initial RNA templates (Ramsköld et al. 2012). Noticeably skewed coverage at the 3′ end of transcripts, which was inversely proportional to the expression level, was observed in both FRs and WRs at comparable levels (Fig. 4C). Taken together with our RT-qPCR data, these results demonstrated that the SIDR method efficiently recovered RNA with minimal loss.

To estimate the accuracy of single-cell expression profiling, we examined gene expression patterns in scRNA-seq data and compared them with the bulk data. When we performed principal component analysis (PCA) and unsupervised hierarchical cluster analysis of gene expression profiles, both FR and WR samples from the same cell lines clustered together (Fig. 4D; Supplemental Fig. S11). Pearson correlation analysis of gene expression showed that cell-to-bulk pairwise correlations were similar between the FR and WR groups (Supplemental Fig. S11C). Additionally, we selected three different well-characterized targets (*CDKN2A, GATA3, MAGEA3*, and *GAPDH*) and generated genomic snapshots of these genes (Fig. 4E). As expected from previous reports (Shapiro et al. 1995; Wascher et al. 2001; Charafe-Jauffret et al. 2006), we found that FR and WR RNA-seq data shared cell line–specific expression patterns highly concordant with the patterns of their matched bulk cells. Consistently, explanatory power values (adjusted *R*^2^) of gene expression in bulk cells (Fig. 4F) show that averaged single-cell data approximated the bulk cell values up to 89%, suggesting that FR single-cell data are consistent with the bulk data comparably to WR data. These results demonstrate that SIDR-seq is suitable for single-cell transcriptome sequencing.

To survey whether a substantial proportion of the variability in single-cell gene expression could be explained by phases of the cell cycle, as previously reported (Buettner et al. 2015; McDavid et al. 2016), we analyzed the transcriptional profiles of bulk and WR MCF7 cells that had been staged for cell-cycle phases (G1, S, or G2/M) by fluorescence-activated cell sorting (FACS) of Vybrant DyeCycle Orange–stained cells (Supplemental Fig. S12A–C). Analyzing cell cycle–staged single cells validated that the cell cycle inferred by cell-cycle marker gene expression patterns of single cells accurately fit to the known stage information (Supplemental Fig. S12C; Whitfield et al. 2002). Principal component analysis (PCA) and unsupervised hierarchical cluster analysis of single cells also showed that FR and WR samples from the same cell lines clustered together regardless of their cell-cycle stages, and that there were no distinct subpopulations based on cell-cycle stages (Fig. 4G,H; Supplemental Fig. S13). These data indicated that the cell cycle did not cause substantial variability in single-cell gene expression that we observed in this study.

### Simultaneous detection of genomic and transcriptomic variation

**Figure 5.**
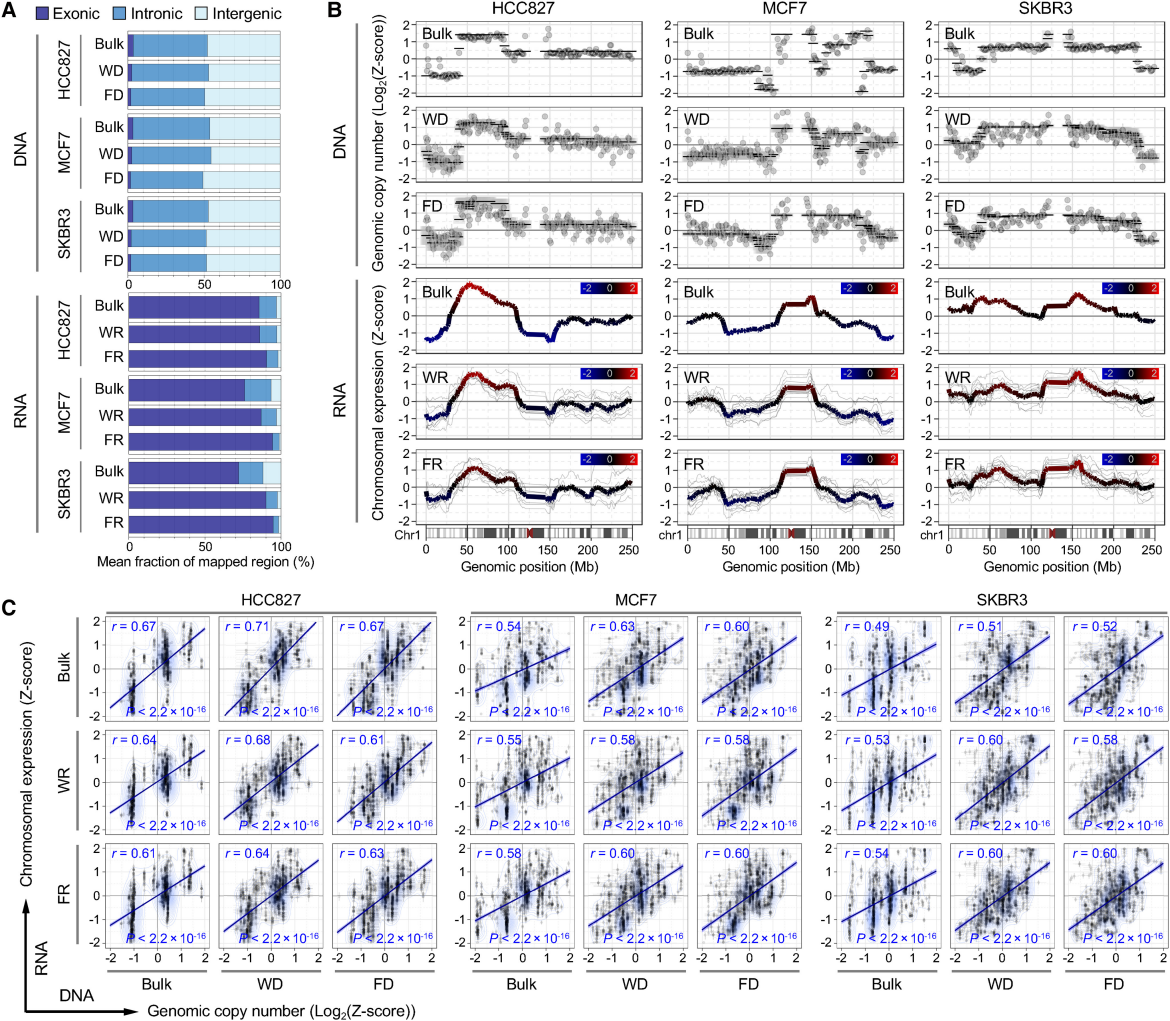
Integration of genome and transcriptome sequencing data generated by SIDR-seq. (*A*) Fractions of sequencing reads mapped to exonic, intronic, and intergenic regions of the human reference genome. Mean fractions of mapped regions were calculated from WGS and RNA-seq. (*B*) Chromosome-wide comparison between genomic copy numbers and gene expression in Chromosome 1. The *upper* three plots show log_2_ ratio of genomic copy numbers (dots) and their CBS-derived segmented values (black lines) estimated from bulk and single cells for DNA sequencing. The *lower* three plots show the chromosomal gene expression values from each single cell (thinner gray line) and their averages (colored line). Chromosomal gene expression correlated with copy-number events in WRs and FRs. Comparison plots for other chromosomes (from Chr 2 to Chr 22) are available in Supplemental Figure S15. (*C*) Correlation between DNA copy numbers and relative gene expression levels binned per 1 Mb genomic scale. Pearson's correlation coefficients (*r*) with their statistical significances (*P*) are shown. Genome sequencing from bulk, WDs, and FDs, and RNA sequencing data from bulk, WRs, and FRs were used for the comparison. (FD) DNA fractionated from single cells by SIDR; (WD) genomic DNA from the whole-cell lysates of single cells; (FR) RNA fractionated by SIDR from single cells; (WR) total RNA from whole-cell lysates of single cells.

We calculated the genome-wide correlation of chromosomal expression levels with copy-number variations (CNVs) at the single-cell level. From 38 sets of paired scSIDR-seq data, 31 genome and 37 transcriptome data qualified and underwent a comparative analysis. When we aligned sequencing reads to the reference genome, the mean fractions of exonic, intronic, and intergenic reads from FDs and FRs were not significantly different from WD and WR data (Fig. 5A). After the alignment of sequencing reads, mean read counts for each segment were used to estimate copy numbers and expression levels of genes in single cells. This analysis showed that the mean expression values of genes correlated with copy-number events. Chromosome-level comparison of DNA copy-number variations and the patterns of expression in single cells showed that the average expression of gene segments was strongly correlated with the copy-number changes of genomic regions (Fig. 5B; Supplemental Fig. S14). Single-cell data from SIDR-seq showed significant positive correlations between genomic copy numbers and mRNA expression levels obtained for bins across the genome (HCC827, Pearson's *r* = 0.63; MCF7, *r* = 0.60; SKBR3, *r* = 0.60; each *P* < 2.2 × 10^−16^) (Fig. 5C). The chromosomal expression data from scSIDR-seq correlate with bulk genomic copy numbers as well as those of WR and FR over the entire genome (Fig. 5C). These positive correlations between chromosomal expression and genomic copy number revealed by scSIDR-seq were consistent with previous studies that successfully inferred large-scale copy-number alterations for each cell by averaging relative expression levels over large genome regions. However, the parallel DNA and RNA sequencing also revealed inconsistencies between chromosome-wide genomic and transcriptomic variations. For example, Chromosome 3 displayed a relatively pronounced discrepancy between CNVs and expression profiles (Supplemental Fig. S15A). Although gene expression showed a trend toward concordance with copy-number changes in general, each single-cell DNA sequencing result showed greater concordance with bulk or other single-cell DNA results than with its own RNA sequencing pair when unsupervised hierarchical clustering was performed for CNVs (Supplemental Fig. S15B). Although single-cell RNA sequencing might be able to reveal copy-number alterations, our data showed that sequencing both the genome and the transcriptome of a single cell by SIDR-seq accurately profiled DNA copy-number variation and gene expression, distinguishing the transcriptional consequences of copy-number variations.

Next, we identified and compared SNVs in RNA and genomic DNA sequences. As previous studies leveraged the power of scRNA-seq to identify SNVs and gene expression variation at the single-cell level, we detected SNVs in single-cell FR and WR samples. We found that, even after removal of potential false positive SNVs via stringent criteria, only ∼35% exonic mutations detected in single-cell FR and WR samples were verified by whole-exome sequencing (WES) results generated from bulk DNA samples (Supplemental Fig. S16A). In contrast, the WES results verified ∼85% of SNVs detected in FD and WD, indicating that SNVs detected by SIDR DNA sequencing were more likely to be true variants than those supported by RNA-seq (Supplemental Fig. S5). For the analysis, we performed WES of nine single-cell FDs, eight single-cell WDs, and two bulk MCF7 cell samples (Supplemental Methods), achieving mean coverage depth of 159.54 ± 12.63, 142.09 ± 17.65, and 111.58 ± 3.56×, respectively. Our results showed that SIDR-seq more precisely captured genetic alterations than did scRNA-seq alone, suggesting that integration of genome and transcriptome sequencing data could provide more reliable and mutually complementary information at the single-cell level.

### Application of SIDR-seq to explore the performance of single-cell sequencing

Using single-cell WGS data sets from SKBR3 cells that were previously published (Wang et al. 2014; Dey et al. 2015), we were able to compare WGS data generated by SIDR-seq with previous data sets from the same cell line generated by different methods. Dey et al. (2015) reported DR-Seq quantifying genomic DNA and mRNA from the same cell without physically separating the nucleic acids before amplification. Wang et al. (2014) used nuc-seq, a single-cell DNA sequencing method developed for accurate description of genetic mutations.

**Figure 6.**
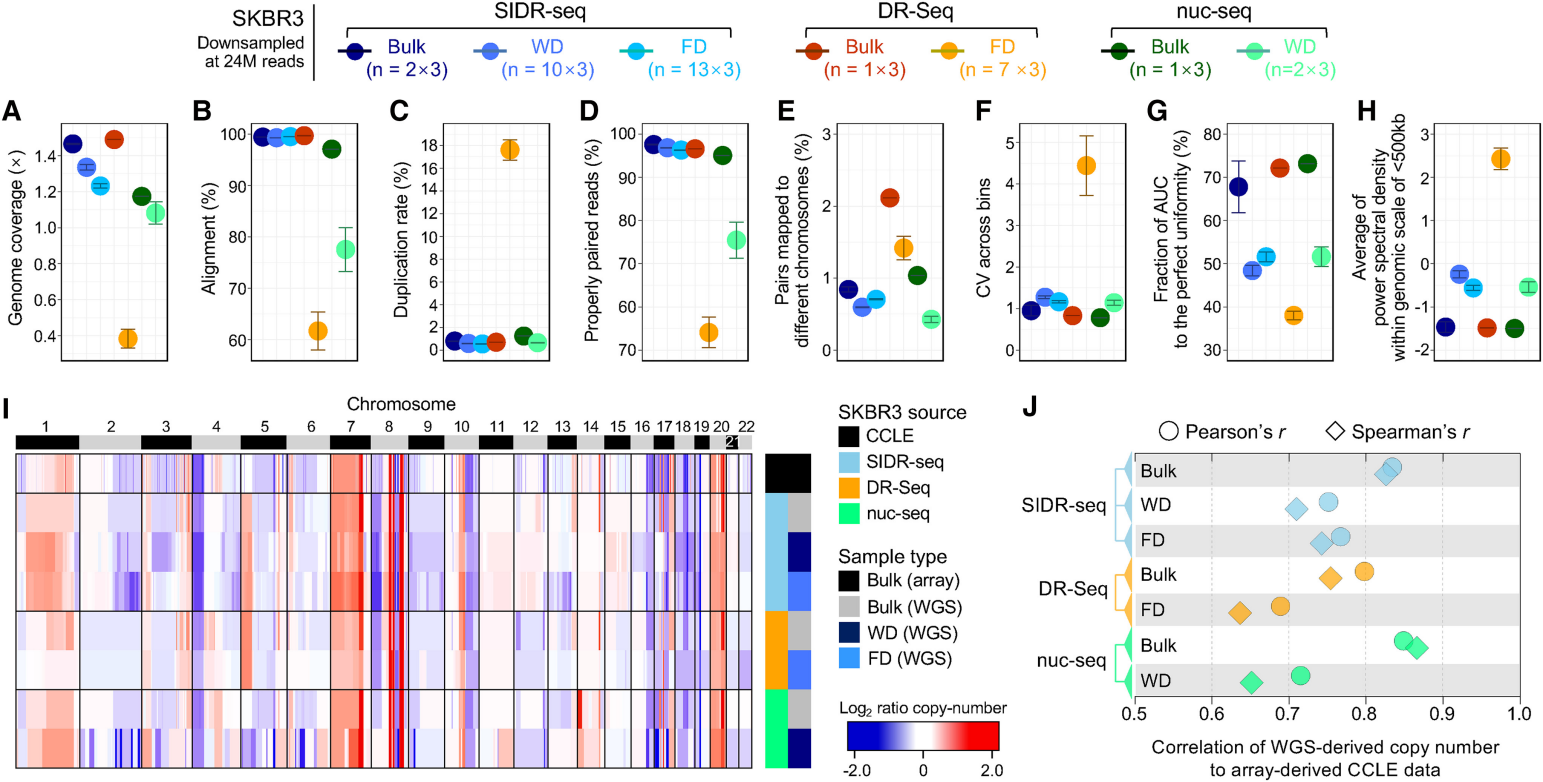
Comparison of single-cell genome sequencing methods: SIDR-seq, DR-Seq, and nuc-seq. The number of sequencing reads in each sample was set to 24 million in triplicate by randomly down-sampling from all available reads. (*A*–*E*) Summary of sequencing metrics. (*A*) Genome sequencing depth of coverage. The plots display fractions of sequencing reads properly aligned to the human reference genome (*B*), duplicated (*C*), properly paired (*D*), and with their paired reads mapped to different chromosomes (*E*). (*F*) Bin-to-bin variabilities in genomic DNA read counts. (*G*) Comparison of coverage uniformities measured by Lorenz curves. The fractions of area under the curve were calculated, averaged for each group, and plotted. (*H*) Comparison of coverage uniformities measured by power spectral analysis. Power spectral densities of read distributions were obtained and averaged across frequencies >1/500 kb. (*I*) Heatmap of genome-wide copy-number profiles in bulk and single cells from SKBR3 cells by binning of 1-Mb genomic scale. Copy-number profiles from genome sequencing were compared to the CCLE data profiled using SNP array (at the *top* of the heatmap). (*J*) Correlation of copy numbers between data sets from each method and CCLE data set. Pearson's and Spearman's correlation coefficients were plotted against the *x*-axis. (FD) DNA fractionated from single cells by SIDR; (WD) genomic DNA from the whole-cell lysates of single cells.

For a fair comparison of data sets, we first performed in silico down-sampling (random selection of a subset of reads) to adjust the data sets of each sample to comparable sizes. The total reads of each sample were down-sampled to 24 million, the size of the smallest data set. Single-cell SIDR-seq mostly displayed comparable or slightly superior data quality to nuc-seq according to various sequencing metrics (Fig. 6A–E; Supplemental Fig. S17). SIDR FDs showed significantly higher rates of alignment (>90%) than other single-cell data generated by DR-Seq or nuc-seq (Fig. 6B) and consequently a larger fraction of properly paired reads than the others (Fig. 6D). As expected at this low depth of coverage, all data sets except DR-Seq showed an extremely low duplication rate (Fig. 6C). We assumed that the high duplication rate of single-cell DR-Seq was in part caused by sequencing reads derived from cDNA, because DR-Seq did not physically separate gDNA and mRNA (Fig. 6C; Supplemental Fig. S17C; Dey et al. 2015; Macaulay et al. 2015). Thus, we estimated the fraction of reads mapped to exonic regions and found that the exonic fraction in FD from DR-Seq (10.63 ± 0.58%) was 5.42 times higher than in FD from SIDR-seq (1.96 ± 0.02%) (Supplemental Fig. S18A). In single-cell DR-Seq data, the duplication rate of reads mapped to coding regions was 25.75 ± 0.35%, remarkably higher than the average duplication rate in the other sample types (0.66 ± 0.10%) (Supplemental Fig. S18B). The physical separation of gDNA from total RNA implemented in SIDR-seq seemed to prevent complications associated with transcript contamination, which was also supported by the coverage uniformity of scSIDR-seq data. WGS data by scSIDR-seq displayed comparable levels of coverage uniformity to WD WGS data from our study and nuc-seq when coverage uniformity was evaluated by CV across bins, AUC of Lorenz curves, and power spectrum of read density (Fig. 6F–H). These satisfactory indexes of sequencing metrics are likely to be the basis for accurate copy-number profiling by scSIDR-seq, as described subsequently.

We found similar trends in genome-wide copy-number profiles between SIDR-seq and array-derived Cancer Cell Line Encyclopedia (CCLE) data (Fig. 6I). When Pearson's and Spearman's correlations of copy numbers were analyzed, the degree of copy-number correlation between single-cell WGS data and the CCLE data was the greatest in scSIDR-seq followed by nuc-seq (Fig. 6J). Because of the minimization of technical losses of gDNA during physical separation, scSIDR-seq performed comparably to bulk sample data in genome sequencing (Fig. 6I,J; Supplemental Figs. S19, S20).

We also compared the overall quality of RNA-seq data profiled by scSIDR-seq to that of single-cell DR-Seq from the same cell line, SKBR3. When the total reads were down-sampled to 0.3 million (triplicates for each sample), while SIDR FRs showed comparable patterns to WRs or bulk cells in uniquely mapping rate and the fraction of reads mapped to exonic regions, FRs in DR-Seq showed considerably lower mappability and lower mapped fraction in exonic regions than data from bulk cells (Supplemental Fig. S21A,B), indicating the presence of reads derived from genomic DNA in RNA-seq data because of the lack of physical separation of these two types of nucleic acids. Although FRs in DR-Seq showed little variability in gene expression for the eight housekeeping genes across all SKBR3 samples (Supplemental Fig. S21D), their global gene expression profiles showed lower correlation with bulk samples generated from SIDR-seq and from DR-Seq compared to SIDR FRs (Supplemental Fig. S21E,F). In addition, compared with the CCLE gene expression array data (SKBR3), scSIDR-seq data showed higher correlations than FRs in DR-Seq (Supplemental Fig. S21G).

Taken together, SIDR-seq enables profiling of both the genome and transcriptome at the single-cell level at a quality comparable or superior to existing methodologies.

## Discussion

We developed an SIDR method, a novel approach that allows simultaneous isolation of genomic DNA and total RNA from single cells. Using robust magnetic separation in combination with an optimized cell lysis condition that preserves genomic DNA within the nucleus, the SIDR method enabled efficient and reliable recovery of genomic DNA and RNA transcripts. We have demonstrated the ability of SIDR to physically separate genomic DNA and total RNA from the same single cell without a discernible loss of either DNA or RNA or cross-contamination of nucleic acids.

Our data showed that under the lysis conditions used for SIDR, the plasma membrane was efficiently ruptured with the release of cytoplasmic components, and the integrity of the nucleus remained relatively well-preserved. Unlike the plasma membrane, which is impermeable to charged molecules, the nuclear membrane, with its plastic nuclear pore complexes, can maintain its integrity under osmotic pressure (Ting-Beall et al. 1993; Vasu and Forbes 2001; Grossman et al. 2012; Svec et al. 2013). Lamin immunostaining showed a clear envelope structure of the nuclear lamina with slight swelling. The nuclear envelope lamina network is known to form a compressed network shell of interconnected rods and thus shows elasticity (Dahl et al. 2004). The preserved integrity of the nucleus likely prevents genomic DNA from leaking into the supernatant fraction, as supported by the observation of minimal contamination of the RNA fraction with genomic DNA. After cell lysis, magnetic beads on the disrupted plasma membrane were sufficient to recover cell lysate, including the nuclei, indicating that the cytoskeletal networks might not completely disappear. In fact, a significant level of actin was present after cell lysis in our study. In a previous study, the presence of substantial amounts of retained cytoplasmic beta actin indicated that the Triton X-100 insoluble actin cytoskeleton still connected the extracellular matrix to the core nucleus after destruction of cells caused by hypotonic lysis with the detergent (Tarone et al. 1984; Gualtieri et al. 2004).

Although the integrity of the nuclear lamina was maintained (Fig. 1B,C), the lipid membrane of the nucleus was not likely to be intact after the cell lysis step, which was supported by the relatively effective recovery of nuclear-enriched mRNA in FR (Fig. 2C; Supplemental Figs. S1, S2). Similar to the nuclear membrane, the membranes of cytoplasmic organelles such as endoplasmic reticulum and mitochondria would not be intact, although all these organelles may be associated with the cytoskeletal networks and trapped in cell lysate. To examine whether cytoplasmic DNA molecules were recovered by the SIDR method, we performed qPCR assays for mitochondrially encoded NADH dehydrogenase 1 (*MT-ND1*) and mitochondrially encoded cytochrome b (*MT-CYB*). Significant fractions of mitochondrial DNA were recovered in FR by the SIDR procedure (Supplemental Fig. S22).

In the present study, isolation of single cells was verified by microscopic observations in each well of the customized 48-well microplate. Regardless of the method used for single-cell isolation, there may be a fraction of compartments that are empty or contain multiple cells. Interpretations based on the data obtained from multiple cells may lead to spurious biological conclusions. The 48-well microplate was designed to reduce the time needed for microscopic scanning, because single cells isolated from primary specimens should be immediately processed for subsequent sample preparation to minimize in vitro artifacts. After a microscopic examination, single cells were lysed in the microplate. We minimized transfer of samples to new tubes in order to reduce the risk of sample loss. Supernatants (total RNA) were transferred to prepared clean tubes, while bead-bound cell pellets (genomic DNA) were captured by placing a magnet on the bottom of the microplate. Alkaline lysis prior to whole-genome amplification was performed in the microplate without transferring cell lysates containing genomic DNA to new tubes.

The performance of the method was evaluated with a specific focus on parallel sequencing of the genome and transcriptome, although analytic methods that could utilize DNA and RNA obtained by SIDR are not limited to massively parallel sequencing. The efficient isolation of genomic DNA and total RNA by SIDR was critical for generation of high-quality single-cell sequencing data. In addition to various sequencing metrics, sequencing analysis of single-cell libraries prepared by the SIDR method revealed genomic and transcriptomic cell-to-cell variation and preserved distinctive signatures of cell lines (Figs. 4, 5). We have shown that single-cell SIDR-seq makes it possible to explore correlations between variation in the genome and the transcriptome at the single-cell level (Fig. 5).

To codetect gene sequences and transcripts from the same single cells, several studies have recently reported novel methods such as microfluidics-facilitated approaches (Han et al. 2014), gDNA-mRNA sequencing (DR-Seq) (Dey et al. 2015), genome and transcriptome sequencing (G&T-seq) (Macaulay et al. 2015), and single-cell triple omics sequencing (scTrio-seq) (Hou et al. 2016). Every technique has its advantages and limitations. For instance, because DR-Seq does not physically separate DNA and RNA before amplification, it may minimize losses of nucleic acids and chances of contamination (Dey et al. 2015). However, because this approach restricts transcript profiling to mRNA and requires in silico masking of the exonic regions of the genomic DNA, it imposes inherent limitations on the choice of the WGA method, detection of genetic variants in exonic regions, and expression profiling of noncoding RNA (Wu et al. 2014; Dey et al. 2015). Our results also indicated that various sequencing metrics for DR-Seq WGS and RNA-seq data were inferior to those for SIDR data because each data set contained a significant fraction of reads derived from the other because of simultaneous amplification of DNA and RNA without physical separation. In contrast, G&T-seq (Macaulay et al. 2015) physically separates polyadenylated [poly(A)] mRNA from genomic DNA by using oligo-dT primer conjugated beads (Raj and van Oudenaarden 2008; Hashimshony et al. 2012; Ramsköld et al. 2012; Wu et al. 2014). Consequently, G&T-seq is not applicable for profiling of nonpolyadenylated transcripts. Numerous functional transcripts, e.g., various noncoding RNAs, including tRNA and rRNA, are known to lack poly(A) tails. For example, among noncoding nonpolyadenylated RNAs, long noncoding RNAs (lncRNAs) form the largest transcript class in the human transcriptome (Yang et al. 2011; Ahadi et al. 2016). Dysregulated expression of lncRNAs may be a clinical marker of many cancers (Ahadi et al. 2016). Approximately 90% of the genome is estimated to be transcribed as noncoding RNA, but the expression and functionality of such molecules remain unclear (Hangauer et al. 2013; Kellis et al. 2014). Recently, analysis of noncoding RNAs at the single-cell level became feasible, as easier-seq was reported to reverse-transcribe total RNA in a polyadenylated-tail-independent manner and then amplify and sequence the RNA of single cells (Fu et al. 2016). In contrast to G&T-seq, which is not applicable for profiling nonpolyadenylated transcripts, RNA fractions isolated by the SIDR method are suitable input material for such polyadenylated-tail-independent methods for examining noncoding RNA expression at the single-cell level. Recently, scTrio-seq was reported to separate DNA and total RNA by cell lysis and subsequent centrifugation (Hou et al. 2016). However, some RNA-containing supernatant was left in the DNA fraction during the separation process to avoid disturbing the nuclear DNA precipitate, leading to a loss of RNA transcripts (Hou et al. 2016). By using magnetic separation in the SIDR method, we retained most of the RNA transcripts without disturbing the genomic DNA fraction. The SIDR method improves integrated analysis of the genome and transcriptome at the single-cell level by separating and recovering genomic DNA/total RNA efficiently and robustly.

In addition to analyzing the single-cell WGS data and evaluating the data quality, we also examined the effect of coverage depth on copy-number profiling. For this purpose, we compared copy numbers of single cells to those of bulk sample data on a genome-wide scale, varying the total read counts in a range of 0.05–24 million by in silico down-sampling. Notably, the results showed that there were no critical distinctions or losses of information with regard to CNV patterns or Pearson's correlation coefficients in down-sampled data sets, even those with the smallest total read count (Supplemental Fig. S20). These results showed that low-depth sequencing of SIDR-prepared samples is sensitive enough to capture copy-number information and uncover genetic heterogeneity across single cells.

Although we applied the SIDR method to analyze cancer cells, there is no reason to limit its application within cancer research. In addition, DNA isolated by the SIDR is suitable for profiling not only genetic alterations but also epigenome characteristics. As recent studies extensively characterizing genome, transcriptome, or epigenome at the single-cell level identified new and rare populations of cells in various organs/tissues (Smallwood et al. 2014; Zeisel et al. 2015; Gawad et al. 2016; Muraro et al. 2016) at different developmental stages (Deng et al. 2014; Scialdone et al. 2016) and pathological status (La Manno et al. 2016; Kee et al. 2017), the SIDR method is anticipated to integrate developmental lineage trees and regulatory status of cells providing new insight into normal development as well as pathogenesis.

Although micromagnetic beads for the method in this study were conjugated with an anti-EPCAM antibody for capturing the cell lysate, other antibodies targeting an appropriate cell surface protein may be used. Our preliminary data showed that an anti-CD33 antibody could be adopted to the SIDR method and recovered 97.3% ± 11.9 of DNA and 89.6% ± 17.8 of RNA fractions from HL-60 cell line samples (*n* = 3), based on LINE-1 and *GAPDH* RT-qPCR, respectively. The data suggested the applicability of SIDR for EPCAM-negative cells, but antibodies against other cell-type–specific surface antigens remain to be characterized for broader application of the method. In addition, cell lysis condition, such as the concentration of Triton X-100, might need to be optimized prior to application of the SIDR method for particular cell types of interest, although cell line samples and clinical tissue specimens tested in this study were well lysed to recover RNA under the condition.

Currently, our method has some limitations in scalability, sensitivity, accuracy of the analysis as well as dependency on antibody–antigen interaction. First, it requires manual manipulation based on microwell dilution, which limits the number of single cells that can be processed concurrently. Second, sample preparation and amplification were performed in microliter volumes in this study. For RNA and DNA sequencing of single cells, a reduction of the reaction volumes from microliters to nanoliters has been recommended to achieve more favorable reaction kinetics and reduce amplification bias (Gole et al. 2013; Wu et al. 2014). In this regard, microfluidic implementation of the method is desirable for automation of the process and reduction of the reaction volume, which might be feasible owing to the simplicity and robustness of SIDR.

In this study, we developed and validated the SIDR method, which allows simultaneous isolation of genomic DNA and total RNA from single cells. By using SIDR, we showed that simultaneous isolation of both genomic DNA and total RNA was feasible with a high recovery yield. Furthermore, scSIDR-seq integrated data about genomic features and RNA expression profiles from the same individual cells, enabling a comprehensive understanding of cellular heterogeneity and complexity at the single-cell level. SIDR is a novel, highly efficient, and simple method of parallel isolation of genomic DNA and total RNA from single cells that can become a promising platform for revealing and understanding a wide range of unknown correlations between genomic/epigenomic alterations and gene expression patterns.

## Methods

### Patient recruitment and tumor samples

A total of five patients diagnosed with breast cancer or lung cancer were recruited for this study. Tissue specimens were obtained from surgical excision without prior treatment. This study was approved by the Institutional Review Board (IRB) of Samsung Medical Center, and all patients provided signed informed consent for collection of specimens and detailed analyses of the derived genetic materials (Institutional Review Board no. 2015-12-094 and no. 2010-04-039).

### Reagents

All materials were used as received, unless otherwise noted. Protein G-conjugated magnetic microbeads were purchased from ThermoFisher Scientific, and buffer reagents were purchased from Sigma. Human anti-EPCAM antibody was purchased from Novus Biologicals (NB100-65094). Anti-lamin B2 and anti-beta actin antibodies were purchased from Abcam (ab8983 and ab8226, respectively).

### Cell culture and fluorescence image analysis

Three commercially available cell lines, MCF7, HCC827, and SKBR3, were obtained from the American Type Culture Collection (ATCC). When cells reached appropriate confluence, they were incubated with membrane staining reagents for 10 min at 37°C. Detailed methods are described in the Supplemental Methods.

### Fabrication of 48-well microplates

Soft-lithography techniques were used to fabricate 48-well microplates. Negative photo resin SU-8 (SU-8 2050; MicroChem) was spin coated on a silicon wafer and a 4-mm-diameter circle array with a 6-mm pitch was patterned by using photolithography to make a master mold. A mixture of PDMS prepolymer (Sylgard 184; Dow Corning Corp.) and curing agents (10:1, v:v) was poured into the master mold and degassed. It was placed on a hot plate for 60 min at 75°C, and then the cured PDMS was removed from the master mold. We punched each circular pattern to form a well structure and bonded them to a clean glass slide after plasma treatment (Cute-MPR; FEMTO Science). Fabricated devices were sterilized with ethylene oxide gas and immediately sealed for long-term storage.

### Single-cell isolation and cell lysis for simultaneous isolation of genomic DNA and total RNA

Detailed experimental procedures are described in the SIDR protocol section of Supplemental Methods. Briefly, dissociated cells prestained by CellTracker (Molecular Probes) were bound to anti-EPCAM antibody-conjugated magnetic beads and diluted to achieve a concentration of one cell/1 µL of PBS. We dispensed 1 µL of cell suspension into each well of the 48-well microplate, examined single-cell isolation by a fluorescence microscopy, and added 9 µL of lysis solution (0.2% Triton X-100 [Sigma-Aldrich] and 0.5% of the RNase Inhibitor [Clontech] in water). After a 10-min incubation at room temperature, supernatants containing total RNA were retrieved, whereas cell lysates including genomic DNA were captured by a magnet placed at the bottom of the 48-well microplate.

### Validation of simultaneous isolation of DNA and RNA by real-time quantitative PCR

Genomic DNA and total RNA from 10 cells underwent physical isolation by using hypotonic lysis followed by magnetic separation. For the quantification of isolated genomic DNA, the LINE-1 locus was amplified by real-time PCR using SYBR Green (Exiqon) according to the manufacturer's protocols. Fractionated total RNA was used as a template for cDNA synthesis with a Single Cell-to-CT Kit (Life Technologies). Detailed validation methods are described in the Supplemental Methods.

### Whole-genome and transcriptome amplification for single-cell sequencing

After hypotonic lysis of each single cell, supernatants (total RNA) and bead-bound cell pellets (genomic DNA) were physically separated. Single-cell whole-genome amplification (Repli-g single cell kit, Qiagen) was performed according to the manufacturer's protocols. Single-cell RNA samples were reverse transcribed and preamplified using SMART-Seq2 according to the manufacturer's protocols (SMARTer Ultra Low Input RNA for Sequencing-v3; Clontech). Detailed step-by-step procedures are described in the Supplemental Methods.

### Parallel sequencing of the whole genome, whole transcriptome, and whole exome

Using 1-ng aliquots of each cDNA sample, a WTS library was prepared using a Nextera XT DNA Sample Prep Kit (Illumina), according to the manufacturer's instructions. Then, the libraries were sequenced on a HiSeq 2500 system using 100-bp paired-end sequencing.

WGS libraries were constructed using a TruSeq Nano DNA Library Prep Kit (Illumina) according to the protocol for sample preparation for multiplexed paired-end sequencing. Low-coverage genome sequencing was performed on an Illumina HiSeq 2500 system with 100-bp paired-end sequencing.

Sequencing libraries for WES were created using the SureSelect XT Human All Exon V5 kit (Agilent Technologies) and subsequently analyzed by the HiSeq 2500 systems (Illumina) using the 100-bp paired-end mode of the TruSeq Rapid PE Cluster kit and TruSeq Rapid SBS kit (Illumina).

### Data analysis

All DNA sequencing data were aligned to build version hg19 of the human genome using BWA-MEM (version 0.7.4) (Li 2013) with default option parameters. Although the more recent human genome assembly GRCh38 has improved contiguity especially in centromeric regions and expanded alternative haplotypes, we do not expect realigning the reads to GRCh38 would have an appreciable impact on our conclusion. Because we compared data sets from genetically homogeneous cell line samples by applying identical processing procedures, our performance evaluation on genome-wide sequencing data should be consistent between the two similar assemblies of high quality. Further data processing procedures are fully described in the Supplemental Methods. Data sets generated in this manuscript can be found in Supplemental Table S1; low-quality cells (defined in Supplemental Fig. S3 and Supplemental Methods) were filtered out in downstream analysis. To compare the quality and performance of SIDR-seq with those of other methods, we downloaded raw FASTQ files of SKBR3 WGS data from DR-Seq (Dey et al. 2015) and nuc-seq (Wang et al. 2014) and applied the identical processing steps.

Processing of RNA-seq data was carried out as described previously (Kim et al. 2015, 2016). Poor-quality cells (defined in Supplemental Fig. S8 and Supplemental Methods) were removed to assess the quality and performance between WR and FR out of SIDR-seq, and SKBR3 RNA-seq data between SIDR-seq and DR-Seq (Dey et al. 2015). Detailed methods for processing RNA-seq data, as well as estimation of relative gene expression levels across samples, are fully described in the Supplemental Methods.

## Data access

The sequencing data from this study have been submitted to the EBI European Nucleotide Archive (ENA; http://www.ebi.ac.uk/ena) under accession numbers ERP022266 (RNA-seq), ERP022267 (WGS), and ERP022268 (WES), respectively.

## Supplementary Material

Supplemental Material
