# Supplementary material for: SIDR: simultaneous isolation and parallel sequencing of genomic DNA and total RNA from single cells
Source: Genome Res. 2018 Jan;28(1):75–87. doi: 10.1101/gr.223263.117 (PMC5749184; doi:10.1101/gr.223263.117)
Supplement: Supplemental Material [file supp_28_1_75__index.html]

SIDR: simultaneous isolation and parallel sequencing of genomic DNA and total RNA from single cells — Supplemental Material 

# SIDR: simultaneous isolation and parallel sequencing of genomic DNA and total RNA from single cells

## Supplemental Material

undefined

- Supplemental\_Material.pdf
