# Supplementary material for: SIDR: simultaneous isolation and parallel sequencing of genomic DNA and total RNA from single cells
Source: Genome Res. 2018 Jan;28(1):75–87. doi: 10.1101/gr.223263.117 (PMC5749184; doi:10.1101/gr.223263.117)
Supplement: Supplemental Material [file supp_gr.223263.117_Supplemental_Material_.pdf]

Supplemental Material

SIDR: simultaneous isolation and parallel sequencing of genomic DNA and total RNA from single cells

Kyung Yeon Han[1,9], Kyu-Tae Kim[1,9], Je-Gun Joung[1,9], Dae-Soon Son[1], Yeon Jeong Kim[1], Areum Jo[1], Hyo-Jeong Jeon[1], Hui-Sung Moon[1], Chang Eun Yoo[1], Woosung Chung[1,2], Hye Hyeon Eum[1,3], Sangmin Kim[4], Hong Kwan Kim[5], Jeong Eon Lee[2,6], Myung-Ju Ahn[7], Hae-Ock Lee[1,8], Donghyun Park[1,*], Woong-Yang Park[1,2,8,*]

[1] Samsung Genome Institute, Samsung Medical Center, Seoul 06351, South Korea

[2] Department of Health Sciences and Technology, SAIHST, Sungkyunkwan University, Seoul 06351, South Korea

[3] Department of Biomedical Sciences, College of Medicine, Seoul National University, Seoul 03080, South Korea

[4] Department of Breast Cancer Center, Samsung Medical Center, Seoul 06351, South Korea

[5] Department of Thoracic and Cardiovascular Surgery, Samsung Medical Center, Seoul 06351, South Korea

[6] Department of Surgery, Samsung Medical Center, Sungkyunkwan University School of Medicine, Seoul, 06351, South Korea

[7] Division of Hematology-Oncology, Department of Medicine, Samsung Medical Center, Sungkyunkwan University School of Medicine, Seoul 06351, South Korea

[8] Department of Molecular Cell Biology, Sungkyunkwan University School of Medicine, Suwon 16419, South Korea

* Corresponding authors: D.P. (dh37.park@samsung.com) and W-.Y.P. (woongyang.park@samsung.com)

[9] These authors contributed equally to this work.

TABLE OF CONTENTS

SUPPLEMENTAL METHODS 3
- Cell culture and pre-treatment 3
- Cell staining for fluorescence image analysis 3
- Antibody conjugation of magnetic microbeads 3
- Western blot analysis 3
- Determining cell cycle stages by flow cytometry 3
- Validation of simultaneous isolation of DNA and RNA by real-time quantitative PCR 4
- Whole genome and transcriptome amplification for single cell sequencing 4
- Parallel sequencing of the whole genome, whole transcriptome, and whole exome 5
- DNA sequencing preprocessing 6
- Copy-number estimation from whole-genome sequencing 6
- Quality control metrics in whole-genome sequencing 6
- Identification of single-nucelotide variants from whole-exome sequencing 6
- RNA sequencing processing 7
- Quality control metrics in RNA sequencing 7
- Estimation of chromosomal expression pattern 8
- Comparison of single-cell sequencing methodologies 9
- Statistical analysis 10

SIDR PROTOCOL 11
- REAGENTS 11
- EQUIPMENT 11
- SAMPLE PREPARATION 11
- PROCEDURE 12

SUPPLEMENTAL FIGURES 16
- Supplemental Figure S1 16
- Supplemental Figure S2 17
- Supplemental Figure S3 18
- Supplemental Figure S4 25
- Supplemental Figure S5 26
- Supplemental Figure S6 27
- Supplemental Figure S7 28
- Supplemental Figure S8 30
- Supplemental Figure S9 36
- Supplemental Figure S10 37
- Supplemental Figure S11 38
- Supplemental Figure S12 40
- Supplemental Figure S13 42
- Supplemental Figure S14 44
- Supplemental Figure S15 48
- Supplemental Figure S16 49
- Supplemental Figure S17 50
- Supplemental Figure S18 52
- Supplemental Figure S19 54
- Supplemental Figure S20 55
- Supplemental Figure S21 56
- Supplemental Figure S22 58

SUPPLEMENTAL TABLE 59
- Supplemental Table S1 59

REFERENCES 60

SUPPLEMENTAL METHODS

Cell culture and pre-treatment

Three commercially available cell lines, MCF7, HCC827, and SKBR3, were obtained from the American Type Culture Collection (ATCC; Manassas, VA). Cell lines were maintained in DMEM (MCF7 cells), RPMI-1640 (HCC827 cells) or McCoy's 5A medium (SKBR3 cells) supplemented with 10% fetal bovine serum (FBS).

Cultured cells were harvested by trypsinization and resuspension in PBS at a concentration of 1×10^6 cells/mL. Cells were identified by pre-staining with CellTracker Green (Molecular Probes, Eugene, OR) following incubation with 5 μM staining dye for 20 min at 37 °C.

Cell staining for fluorescence image analysis

When cells reached appropriate confluence, they were washed with the Live Cell Imaging buffer (Life Technologies, Waltham, MA) and incubated with 1000-fold diluted CellMask Deep Red plasma membrane stain (Life Technologies, Waltham, MA) at 37 °C for 10 min. Fluorescently labeled cells were harvested by trypsinization and resuspension in PBS. Membrane-stained cells were incubated with a nuclear membrane targeted anti-lamin B2 antibody at 37 °C for 30 min. After immunostaining, cells were mounted using VECTASHIELD antifade mounting medium with DAPI (Vector Laboratories, Burlingame, CA) and visualized under an inverted microscope with a motorized stage (Olympus, Japan).

Antibody conjugation of magnetic microbeads

Protein G-conjugated magnetic microbeads (2.8 μm) were purchased from ThermoFisher Scientific. The magnetic beads were incubated with the anti-EpCAM antibody at room temperature for three hours. The antibody-conjugated magnetic beads were washed three times with PBS to remove any residual antibodies and stored in 0.1% BSA solution at 4 °C for up to one month.

Western blot analysis

Bead-bound and intact MCF7 cells were lysed in the hypotonic lysis buffer. Equal amounts of cell extracts were subjected to 4 to 12%-gradient SDS-PAGE and transferred onto a nitrocellulose membrane for western blotting. The membranes were developed using an HRP-conjugated substrate kit and protein quantities were analyzed using ImageQuant™ LAS 400 (GE Healthcare, Pittsburgh, PA).

Determining cell cycle stages by flow cytometry

For the use of fluorescence-activated cell sorting (FACS) to collect cells based on the cell-cycle stages, Vybrant® DyeCycle™ Orange stain (Life Technologies, CA) from the freezer was equilibrated to room temperature prior to the use. Cells were pelleted and resuspended in 1 mL complete culture media at a concentration of 1×10^6 cells/mL. DyeCycle™ Orange stain (2 μL; Life Technologies, CA) was added (final staining concentration 10 μM), and the cells were incubated at 37˚C for 30 minutes, protected from light. After staining, a FACS machine was used to sort G1-phase, S-phase, and G2/M-phase samples of the MCF7

cell line according to the manufacturer's instructions and cell samples were analyzed on flow cytometer using 488 nm excitation or 532 nm excitation and orange emission.

Validation of simultaneous isolation of DNA and RNA by real-time quantitative PCR

For bead-cell binding, 2 μL of EpCAM-conjugated magnetic beads were incubated with ~1×10^4 cells in 100 μL PBS at 37 °C for 30 min. The approximate concentration of CellTracker pre-stained cells was measured by using a Countess automated cell counter (Invitrogen, Waltham, MA). Next, the cells were diluted and counted to achieve a concentration of 10 cells/μL, as previously described (Park et al. 2012). Genomic DNA and total RNA from 10 cells underwent physical isolation by using hypotonic lysis followed by magnetic separation. For the quantification of isolated genomic DNA, the LINE-1 locus was amplified by real-time PCR using SYBR Green (Exiqon, Woburn, MA) according to the manufacturer's protocols. The LINE-1 locus region was amplified by the following pair of PCR primers: 5′-TCACTCAAAGCCGCTCAACTAC-3′ and 5′-TCTGCCTTCATTTCGTTATGTACC-3′. The Cp (crossing point) values of the target gene were determined by the LightCycler 480 software (Roche, Branchburg, NJ). For the quantification of mitochondrial DNA, 1,000 cells were used for TaqMan assays (Life Technologies) were used: *MT-ND1* (forward primer: 5'-TCACAACACAAGAACACCTCTGATT-3'; reverse primer: 5'-TTGGTCTCTGCTAGTGTGGAGATA-3') and *MT-CYB* (forward primer: 5'-CTTTCACTTCATCTTGCCCTTCATT-3'; reverse primer: 5'-CCCGTTTCGTGCAAGAATAGGA-3').

Fractionated total RNA was used as a template for cDNA synthesis with a Single Cell-to-CT™ Kit (Life Technologies, Waltham, MA). One unit of DNase I was added to each RNA sample and the RNA mix was incubated at room temperature for 10 min. After adding one unit of the stop solution, reverse transcription and subsequent pre-amplification were performed according to the manufacturer's protocols. The TaqMan® Gene Expression Assays for *GAPDH*, *CDKN1A*, *PSMC4, GATA6, APBB2,* and *SVIL* (Hs03929097_g1, Hs00355782_m1, and Hs00197826_m1, Hs00232018_m1, Hs00921383_m1, and Hs00931004_m1, respectively, Life Technologies, Waltham, MA) were pooled and diluted to a final concentration of 0.2× in 1× TE buffer, pH 8.0. Before performing real-time PCR, pre-amplified cDNA was diluted 20-fold in a 1×TE buffer, pH 8.0. For the relative quantification of ribosomal RNAs, the following probes for the TaqMan® Gene Expression Assay (Life Technologies) were used: *18S rRNA* (Hs99999901_s1) and *5S rRNA* (forward primer: 5'- CGCCCGATCTCGTCTGAT-3'; reverse primer: 5'-GGTCTCCCATCCAAGTACTAACCA-3'). The C_T (cycle threshold) values of the target regions were quantified by the LightCycler 480 software and normalized in relation to the *GAPDH* signal.

Whole genome and transcriptome amplification for single cell sequencing

Detailed step-by-step procedures are described in the section of SIDR protocol. After hypotonic lysis of each single cell, supernatants (total RNA) and bead-bound cell pellets (genomic DNA) were physically separated by placing a magnet on the bottom of the 48-well microplate.

Total RNA fractions suspended in hypotonic lysis buffer were transferred to clean tubes. Whole RNA samples from single cells were also processed by hypotonic lysis. Single-cell RNA samples were reverse

transcribed and pre-amplified using SMART-Seq2 according to the manufacturer's protocols (SMARTer® Ultra™ Low Input RNA for Sequencing-v3; Clontech, Mountain View, CA).

To account for technical noise of scRNA-seq, External RNA Controls Consortium (ERCC) spike-in RNAs (Life Technologies, Carlsbad, CA) were added to the part of single-cell RNA samples (14 single cells of MCF7 cell line). 6 FRs and 8 WRs were processed with ERCC spike-ins. 0.45 μL of a 1:1,000,000 dilution of ERCC spike-in mixture was supplemented to each RNA sample. For ERCC spike-in experiments, cells were prepared from the same batch, sorted with the same batches of reagents.

After isolating RNA fractions, PBS was added to the cell-bead pellets containing genomic DNA to bring the volume up to 4 μL. For whole cell lysate control samples, single cells without fractionating RNA were also prepared in 4 μL PBS buffer. Single-cell whole genome amplification (Repli-g single cell kit, Qiagen, Hilden, Germany) was performed according to the manufacturer's protocols.

Parallel sequencing of the whole genome, whole transcriptome, and whole exome

Prior to quality validation, PCR-amplified cDNA was purified by using an Agencourt Ampure XP Kit (Beckman Coulter, Brea CA) according to the manufacturer's protocols. After purification, 1-μL aliquots of the amplified cDNA from each sample were validated using a High Sensitivity DNA Chip from the Agilent High Sensitivity DNA Kit as described by the user manual (Agilent, Santa Clara, CA). A Qubit® 2.0 Fluorometer (Life Technologies, Waltham, MA) was also used to quantify the amount of synthesized cDNA. For scRNA-seq library construction, we used the amplified cDNA samples that had distinct peaks spanning from 400 to 10,000 bp, peaked at ~2,000 bp and yielded approximately 2–10 ng of cDNA, according to the manufacturer's guidelines. Using 1-ng aliquots of each cDNA sample, a WTS library was prepared using a Nextera XT DNA Sample Prep Kit (Illumina, San Diego, CA), according to the manufacturer's instructions. Then, the libraries were sequenced on a HiSeq 2500 system using 100-bp paired-end sequencing.

Quantity and quality of the amplified genomic DNA were assessed using a Qubit® 2.0 Fluorometer (Life Technologies, Waltham, MA) and a Nanodrop (Thermo scientific, Waltham, MA). For the preparation of WGS and WES libraries, only samples with amplified genomic DNA yields of approximately 40 μg per 50 μL reaction volume with a purity greater than 1.8 (A260/A280) were processed for shearing using an S220 Focused-ultrasonicator (Covaris, Woburn, MA). WGS libraries were constructed using a TruSeq Nano DNA Library Prep Kit (Illumina, San Diego, CA) according to the protocol for sample preparation for multiplexed paired-end sequencing. Low-coverage genome sequencing was performed on an Illumina HiSeq 2500 system with 100-bp paired-end sequencing.

To investigate the correlations of variants between the genome and transcriptome, we performed deep WES in 17 single MCF7 cells and 2 bulk MCF7 samples. Sequencing libraries for WES were created using the SureSelect XT Human All Exon V5 kit (Agilent Technologies, Inc., Santa Clara, CA), and subsequently analyzed by the HiSeq 2500 systems (Illumina, San Diego, CA) using the 100-bp paired-end mode of the TruSeq Rapid PE Cluster kit and TruSeq Rapid SBS kit (Illumina). Mean target coverage for exome data was 147.14 ± 42.87×.

DNA sequencing preprocessing

Whole-genome and whole-exome sequencing reads were aligned to the human reference genome (version hg19) using BWA-MEM (version 0.7.4) (Li 2013) with default parameters. Putative duplicates amongst the mapped reads were marked using the MarkDuplicates module in the Picard tool (version 1.118, http://broadinstitute.github.io/picard). Sites potentially harboring small insertions or deletions were realigned using the IndelRealigner module, and base qualities in sequence reads were recalibrated using the BaseRecalibrator module in the GATK tool (version 3.2-2) (DePristo et al. 2011) with dbSNP (version 137) (Sherry et al. 2001), 1000G (phase 1)(The 1000 Genomes Project Consortium 2015) and HapMap (phase 3)(The International HapMap 3 Consortium 2010) data for known polymorphic sites.

Copy-number estimation from whole-genome sequencing

Genomic copy-number was estimated from low-coverage whole-genome sequence read density by dividing the genome into bins of variable length and counting the number of unique reads in each bin. As described in the previous approach introduced by Navin *et al*. (Navin et al. 2011), the variable bin size was adjusted depending on the mappability of sequences to regions of the human genome so that each bin had an equal number of reads. The median genomic length spanned by each bin was 500 kb. Regions surrounding centromeres were masked to remove false-positive amplification events. With regard to GC-content bias in the bins, total depth-adjusted log ratio bin-counts were normalized by the LOWESS (locally weighted scatterplot smoothing) fitting with the smoothing parameter of 0.3. Segmented copy-number profiles were estimated using the circular binary segmentation (CBS) algorithm implemented in the DNAcopy package (Seshan and Olshen 2016) with a significance level of 0.001 required for the test to accept change-points. Sex chromosomes were excluded in downstream analysis.

Quality control metrics in whole-genome sequencing

The alignment statistics of the BAM files were assessed using the flagstat function in SAMtools (Li et al. 2009). In order to assess genome-wide coverage distribution, we first calculated the coefficient of variation (CV) of bin-counts in each sample. Next, as previously performed in Zong *et al*. (Zong et al. 2012), we constructed Lorenz curves to estimate the cumulative fraction of reads according to the cumulative fraction of the genome covered at increasing read depths. In addition, we performed a power spectral density analysis to measure the read depth variability using the periodogram function with the "smooth" method in the GeneCycle package (Ahdesmaki et al. 2015). As shown in Supplemental Fig. S3, samples satisfying all of the following conditions were included in downstream analysis: 1) rate of uniquely mapping to the genome reference ≥0.8; 2) first quartile of bin-counts ≥100; 3) CV of bin-counts <2; 4) in Lorenz curves, fraction of area under the curve (AUC) to the perfect coverage uniformity ≥0.3; 5) in power spectral density curves, average values within genomic scale less than 500 kb <1.

Identification of single-nucelotide variants from whole-exome sequencing

The HaplotypeCaller module in the GATK tool (version 3.2-2) (DePristo et al. 2011) to detect single-nucelotide variants (SNVs) was employed for WES data with the known polymorphic sites from dbSNP

(version 137) (Sherry et al. 2001), 1000G (phase 1) (The 1000 Genomes Project Consortium 2015) and HapMap (phase 3) (The International HapMap 3 Consortium 2010). When calling the variants, we applied the option parameters as follows: -stand_call_conf 30.0 -stand_emit_conf 10.0. We filtered variants that passed the recalibration processes by applying the VariantRecalibrator module (with the option parameters: -an QD -an MQRankSum -an ReadPosRankSum -an FS -an DP -mode SNP --maxGaussians 4) and the ApplyRecalibration module (with the option parameters: --ts_filter_level 99.5 -mode SNP) in the GATK tool. Filtered variants with minimum depth ≥10 were annotated using the SnpEff tool (Cingolani et al. 2012) with the hg19 database, and exonic variants were additionally filtered to compare with SNVs called from RNA-seq.

RNA sequencing processing

RNA-seq data were processed as previously described (Kim et al. 2015; Kim et al. 2016). In brief, RNA sequence reads were aligned to the human reference genome (hg19) using the 2-pass default mode of STAR (version 2.4.0i) with the annotation of GENCODE (version 19) (Harrow et al. 2012). Using the same reference and annotation, gene expression was quantified in units of TPM (transcript per million) applying RSEM (version 1.2.18) (Li and Dewey 2011) with default option parameters. TPM values less than one were considered unreliable and substituted with zero. Genes of zero expression across all single cells were initially removed. To assess the ERCC transcripts in a subset of MCF7 samples, we prepared the additional reference and annotation by adding the given ERCC sequences and corresponding annotation that were provided from the manufacturer (Ambion, Carlsbad, CA).

Detection of SNVs in RNA-seq data was carried out as previously described (Kim et al. 2015). Briefly, aligned reads were additionally processed by adding read groups (using the AddOrReplace ReadGroups module of Picard), sorting (using the sort function of SAMtools), marking for duplicate reads (using the MarkDuplicates module of Picard), splitting into exon segments (using the SplitNCigarReads module of GATK), hard-clipping any sequences overhanging the intronic regions (using the RealignerTargetCreator module of GATK), realigning (using the IndelRealigner module of GATK) and recalibrating (using the BaseRecalibrator module of GATK). Variant calling was performed using the HaplotypeCaller module of GATK with option parameters as follows: -recoverDanglingHeads –dontUseSoftClippedBases -stand_emit_conf 20 -stand_call_conf 20. Called variants were then initially filtered using the VariantFiltration module of GATK with option parameters as follows: -window 35 -cluster 3 -filterName FS -filter "FS > 30.0" -filterName QD -filter "QD < 2.0". Variants with minimum depth ≥5 and variant call quality Q >20 were then further filtered to reduce potential false positives, using the SNPiR approach (Piskol et al. 2013). Exonic SNVs from RNA-seq data were compared with exome-sequencing profiles.

Quality control metrics in RNA sequencing

To identify genes that are expressed at high levels and with low variation across cancer cells, we analyzed mRNA expression profiles of cancer cell lines from the Cancer Cell Line Encyclopedia (CCLE) (Barretina et al. 2012). We downloaded the pre-normalized mRNA expression data of 1,037 cancer cell lines (http://www.broadinstitute.org/ccle, CCLE_Expression_Entrez_2012-09-29.gct) that were profiled using

the Affymetrix array platform. When we selected genes highly expressed with little variability by applying the cutoff values (average gene expression >10 and standard deviation <0.25, respectively), eight housekeeping genes were commonly identified across the total (n = 1037), breast (n = 59) and lung (n = 187) cancer cell line groups (Supplemental Fig. S6). We considered these eight genes (*RPL5*, *RPS19*, *RPL13A*, *RPL37A*, *RPS16*, *CALM2*, *GAPDH* and *RPS11*) as constitutively expressed with minimal variability across individual cancer cells, and thereby used them as markers to identify poor quality scRNA-seq data. The expression of these eight genes was also validated by high correlations between bulk RNA-seq data and CCLE data (Supplemental Fig. S7). Compared to corresponding bulk cell RNA-seq data, scRNA-seq data showing > 3 fold-changes of $\log_2$ expression values in any of these markers were of poor quality, as coherently indicated by lower rates of unique mapping, higher rates of duplicated reads and fewer detected genes (Supplemental Fig. S8). These data suggest that variable expression of these genes in single cells is highly likely to be due to technical variability rather than biological causes. Indeed, after filtering poor quality cells, these markers were stably expressed with little variability across individual cells, as shown in Supplemental Figs. S9, S11A and S13A.

On the other hand, we also sought to identify highly variable genes that can be used to distinguish cells. Applying the previous approach implemented in Seurat package (Satija et al. 2015), we calculated the average and dispersion of gene expression across samples. The measure of dispersion was estimated by placing genes into 20 bins based on average expression ($\log_2$(TPM + 1)) and quantifying log ratio Z-scores of the variance divided by the mean for each gene across samples. Variable genes were selected using the cutoff of average expression >3 and dispersion >1.5, and discriminated the intrinsic expression profiles between three cell lines regardless of samples types, as shown in Fig. 4D and Supplemental Fig. S11B.

In addition, we evaluated whether cell-cycle phases significantly contributed to inter-cellular variability of gene expression profiles, which might affect subgrouping samples. To validate the determination of cell-cycle phases based on expression profiles, we sorted MCF7 cells according to G1-, S- and G2/M phase, respectively, as described in the section, 'Single cell sorting using 48-well microplates or flow cytometry'. We assessed the relative expression levels of the reported cell-cycle markers (Whitfield et al. 2002), and determined the specific cell-cycle phase based on the most enriched gene-set signature (Z-score). As expected, gene-set activation increased in accordance with the corresponding cell-cycle phase in bulk cells as well as in single cells, as shown in Supplemental Fig. S12B,C. The other single-cell samples randomly collected were profiled to determine their cell-cycle phases and investigate how many cell-cycle related genes were found among variable genes within each cell line. Only small fraction of variable genes was cell cycle-related genes (approximately 5.56% when applying the cutoffs of average expression >3 and dispersion >1.5), which had little effect on how cells were subgrouped based on variable gene expression profiles as shown in Fig. 4G and Supplemental Fig. S13.

Estimation of chromosomal expression pattern

We estimated chromosomal expression patterns in RNA-seq data to compare with genomic copy-number profiles in WGS data, following the previous approach introduced by Tirosh *et al.* (Tirosh et al. 2016). In

order to remove background signals of gene expression in lung cancer (HCC827) and breast cancer (MCF7 and SKBR3) cells, we used non-patient expression profiles as tissue-matched references. Raw FASTQ files for lung (ERR030879) and breast (ERR030883) tissues were downloaded from the Illumina Human Body Map 2.0 Project. Given the read length of these FASTQ files (paired-end 2×50 bp RNA-seq), we prepared another reference file to be used in STAR alignment, and applied the same processing pipeline that we used for our data.

We first transformed our expression profiles to $\log_2((TPM/10)+1)$ values, and filtered out genes which met either of the following conditions: 1) gene expression was all zero in at least one of cell lines, and 2) the average expression level of the gene was lower than that of total genes in each cell line or in all samples. The remaining 6,318 genes were used to estimate the ratio of gene expression levels in each cancer sample to those in the tissue-matched references. Given the variability of gene expression ratios across cancer samples, relative gene expression signatures were quantified by Z-score transformation. We sorted genes by their genomic locations, and then calculated a moving average with a window of 150 analyzed genes in order to smooth transcriptional variance along the genomic coordinates with enhanced the trends and patterns. We compared transcriptional expression along the genomic coordinates with genomic copy-number to estimate the correlation between these two profiles across all sample types. For the analysis, segmented genomic copy-number profiles and smoothed transcriptional expression profiles were identically binned to 1 Mb, and transformed to Z-scores.

Comparison of single-cell sequencing methodologies

To compare the quality and performance of SIDR-seq with those of other methods, we downloaded previously published raw FASTQ files of SKBR3 WGS data: a bulk cell sample (SRR1639624) and three single cells (SRR1639625, SRR1639626 and SRR1639627) from DR-Seq (Dey et al. 2015), and a bulk cell sample (SRR504589) and two single cells (SRR504599 and SRR504607) from nuc-seq (Wang et al. 2014). For the comparison, each WGS data was down-sampled to generate a series of data with 0.05, 0.1, 0.2, 0.3, 0.5, 0.75, 1, 2, 3, 5, 7.5, 10, 12.5, 15, 17.5, 20, and 24 million total read counts in triplicate, and applied the same pipeline. Because DR-Seq sequencing libraries were constructed without physical separation of the nucleic acids before amplification (Dey et al. 2015), we also re-aligned our and public SKBR3 WGS data to the genome reference masked in coding regions as previously described in Dey *et al.*, and processed the other steps identically as performed with the original genome reference.

We evaluated the correlation of copy-number profiles not only between WGS obtained by different methods, but also between WGS data and SNP array data. We downloaded the normalized SKBR3 Affymetrix SNP data from CCLE, which is segmented using the CBS algorithm (http://www.broadinstitute.org/ccle, CCLE_copynumber_2013-12-03.seg.txt). To compare their relative ratios, we applied a 1 Mb bin along the genome.

In addition, to compare the quality of single-cell RNA-seq of SIDR-seq to those of DR-Seq (Dey et al. 2015), we downloaded raw FASTQ files of SKBR3 RNA-seq data: a bulk cell sample (SRR1639637) and a barcode-indexed single-cell sample (SRR1639638). Single cells from DR-Seq data (SRR1639638) were sorted according to the cell-specific barcodes and subjected to the following analysis when their number

of reads was higher than 0.1 M (n = 21). Sequencing reads of all SKBR3 RNA-seq samples were down-sampled to 0.3 M total read in triplicate. For comparison with SKBR3 bulk cells profiled by gene expression microarray from CCLE, gene expression from SKBR3 bulk and single-cell RNA-seq data was adjusted by the ComBat method (Johnson et al. 2007) implemented in the sva package (Leek et al. 2012).

Statistical analysis

All values are represented as the mean ± s.e.m (standard error of the mean). Linear regression was applied to scatter plots with Pearson's correlation coefficient represented (r). Its statistical significance (P) was calculated using two-sided Student's t-tests. To compare copy-numbers or gene expression across samples, we performed Z-score transformation. Multiple regression analysis was carried out to test the explanatory power of the transcriptomes of different sized pools of single cells to those of bulk cells, as previously described (Kim et al. 2015; Kim et al. 2016). Adjusted R-squares of multiple regression analysis were calculated by random sampling of single cells with 1,000 iterations.

SIDR PROTOCOL

REAGENTS

- Cells or tissue samples ready for isolation.

- CellTracker Green (Molecular Probes, Eugene, OR, USA)

- Distilled water (ThermoFisher Scientific, Waltham, MA, USA)

- PBS (ThermoFisher Scientific)

- Protein G-conjugated magnetic microbeads (ThermoFisher Scientific)

- Antibody (Appropriate antibody targeting surface antigens of cell lines or tissue samples)

- Triton X-100 (Sigma Aldrich, St. Louis, MO, USA)

- (Optional) External RNA Controls Consortium (ERCC) spike-in RNAs (Life Technologies, Carlsbad, CA, USA)

- (Optional) 10× Lysis Buffer - v3 (Clontech, Mountain View, CA, USA)

- Buffer DLB, stop solution, 1 M DTT (Repli-g single cell kit; Qiagen, Hilden, Germany)

- 3′ SMART CDS Primer II A, 5× first-strand buffer, DTT, dNTP Mix, SMARTer IIA Oligonucleotide, RNase Inhibitor, and SMARTScribe Reverse Transcriptase (SMARTer® Ultra™ Low Input RNA for Sequencing-v3; Clontech)

- REPLI-g sc Reaction Buffer, REPLI-g sc DNA Polymerase (Repli-g single cell kit; Qiagen)

EQUIPMENT

- 48-well microplates (Fabrication method is described in the Methods section of the main text)

- Motorized microscopy (e.g., Olympus, Tokyo, Japan)

- Thermal cycler (e.g., Bio-Rad)

- Cell counter (e.g. Countess, ThermoFisher Scientific)

- Vortexer (e.g. Scientific Industries, Bohemia, NY, USA)

SAMPLE PREPARATION

- FD: fractionated genomic DNA by the SIDR method

- FR: fractionated total RNA by the SIDR method

- WD: whole-cell lysates containing genomic DNA used as control preparations for the comparison of genomic DNA concentrations

- WR: whole-cell lysates containing total RNA used as control preparations for the comparison of RNA concentrations

PROCEDURE

Cell collection and pre-treatment; TIMING 1 h, depending on samples and number of cells

1. Harvest cells into a sterile conical tube

✓ Optional: Dissociation of single cells from cancer tissues

- Enzymatically digest cancer tissues for 2 h.

- Centrifuge cell suspensions with Ficoll-Paque PLUS (GE Healthcare, Uppsala, Sweden) to remove dead cells.

2. Pre-stain harvested cells following incubation with 5 μM CellTracker Green staining dye for 20 min at 37°C

Cell-bead conjugation and density measurement; TIMING 0.5 h

3. Measure the density of CellTracker pre-stained cells with a fluorescence cell counter.

4. Add 2 μL of antibody-conjugated magnetic beads (Methods section in the main text) to 100 μL of cell suspension at a concentration of 1×10^5 cells/mL

5. Rotate cell-bead mixtures at room temperature for 20 min.

6. Place the tube containing cell-bead mixture using a magnetic stand for 1 min and remove the supernatant.

7. Resuspend the cell-bead mixture from Step 6 with 100 μL of fresh PBS.

8. Dilute the bead-bound cells to achieve a concentration of 1 cell/μL.

Single cell sorting; TIMING 1 h, depending on method and number of targeting cells

9. Manually pipette 1 μL of single cell suspensions into the 48-well microplate and confirm their placement by motorized microscopy. Optionally, Steps 8 and 9 can be conducted using micromanipulators.

10. Select and mark appropriate numbers of wells containing single cell.

Simultaneous isolation of genomic DNA and total RNA; TIMING 1 h

11. Prepare hypotonic lysis buffer for the SIDR method combining the following components. Scale-up as needed.

Component	Volume per sample (μL)	Volume for 48 samples (25% overage) μL
1% Triton X-100	1.8	108
RNase Inhibitor	0.05	3
Antibody-conjugated magnetic beads	0.09	5.4
Distilled water	7.06	423.6
Total volume	9	540

✓ Optional I: Addition of ERCC spike-ins

- Add ERCC spike-in mixture (0.45 μL of a 1:1,000,000 dilution per sample) into a hypotonic lysis buffer above.

12. Pipette 9 μL of HLB into each well of the 48-well microplate containing sorted single cells.

✓Optional I: Cell lysis for WR control samples

- Prepare 1× lysis buffer by mixing 160 μL of distilled water with 19 μL of 10× Lysis Buffer and 1 μL of RNase Inhibitor for 20 samples and vortex briefly to mix

- Pipette 9 μL of 1× lysis buffer into each well of the 48-well microplate containing sorted single cells for WR control samples.

13. Incubate for 10 min at room temperature.

14. Isolate RNA molecules from single cells.

(i) After incubation, place the 48-well microplates containing single cells for SIDR onto a magnet for 1 min.

(ii) Retrieve lysis solution containing total RNA, whereas cell lysates including genomic DNA are captured by a magnet placed at the bottom of the 48-well microplate.

(iii) Name the retrieved fractions as FRs and residual cell lysates as FDs. The volume recovered should be ~10 μL.

(iv) Add PBS into each well of 48-well microplates containing genomic DNA to bring the volume to 4 μL.

✓Optional I: Collect lysis solution from WR samples and transfer to clean tubes after incubation.

✓Optional II: Prepare 10 ng of purified RNA extracted from each sample for bulk RNA at a concentration of 1 ng/μL in triplicate.

15. Prepare a denaturation buffer for the whole genome amplification reaction by mixing 2.75 μL of Buffer DLB and 0.25 μL of 1 M DTT. Scale-up as needed.

✓Optional I: Add 3 μL of PBS into WDs from Step 10 and perform the following steps.

16. Add 3 μL of denaturation buffer to FDs from Step 14.

17. Incubate at 65°C for 10 min.

18. After cell lysis, add 3 μL of stop solution and store on ice.

19. Place the 48-well plate containing FDs onto a magnet to retrieve genomic DNA from excess bead components and transfer the solutions into clean tubes.

Optional: Prepare 1 μg of purified DNA extracted from each sample for bulk DNA in triplicate.

Whole genome amplification; TIMING 8 h

20. Prepare the master mix for whole genome amplification (Repli-g single cell kit) according to the manufacturer's protocols.

21. Incubate genomic DNAs from single cells with master mix at 30°C for 8 h followed by polymerase inactivation at 65°C for 3 min.

Reverse transcription and whole transcriptome amplification; TIMING 3 h

22. Place RNA samples (WRs, FRs, or purified RNAs) on ice, and add 1 μL of 3′ SMART CDS Primer II A.

23. Place the tubes into a preheated thermal cycler and run the following program:

72°C, 3 min

4°C, forever

24. Prepare a Master Mix for reverse transcription according to the manufacturer's protocols:

Component	Volume per sample (μL)	Stock Concentration
5× First-Strand Buffer	4	
DTT	0.5	100 mM
dNTP Mix	1	20 mM
SMARTer IIA Oligonucleotide	1	12 μM
SMARTScribe Reverse Transcriptase	2	100 U/μL
RNase Inhibitor	0.5	40 U/μL
Total volume	9	

25. Add 9 µL of the Master Mix to each reaction tube from Step 23.

26. Mix the contents of the tubes by gently pipetting and incubate at 42°C for 90 min, followed by 70°C for 10 min for the first-strand cDNA synthesis.

27. After cDNA synthesis, prepare a PCR Master Mix Combine the following reagents according to the manufacturer's protocols.

Component	Volume per sample (µL)
2× SeqAmp PCR Buffer	25
PCR Primer II A - v3	1
SeqAmp DNA Polymerase	1
SMARTer IIA Oligonucleotide	1
Nuclease-free water	2
Total volume per reaction	30

28. Perform LD PCR (long-distance PCR) for amplification using the PCR Master Mix from Step 27 with the following PCR protocol: 95°C for 1 min; X cycles of 98°C for 10 s, 65°C for 30 s, 68°C for 3 min; 72°C for 10 min. For single cell-derived RNA, X was 24 cycles; for the RNA from bulk cells, X was 9 cycles.

Supplemental Figure S1

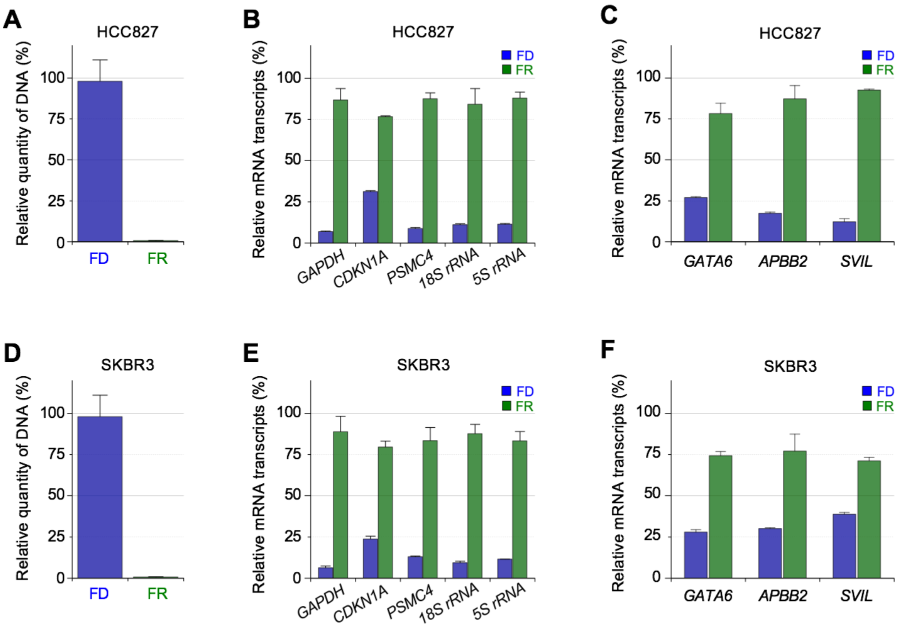

Supplemental Figure S1. Recovery rates of DNA (*A,D*) and RNA (*B,C,E,F*) by the SIDR method in HCC827 (*A–C*) and SKBR3 (*D–F*) cell lines. The efficiency of DNA recovery by the SIDR method was estimated by real-time PCR targeting the LINE-1 locus. The efficiency of RNA recovery by the SIDR method was estimated by real-time PCR targeting (*B,E*) cytoplasmic RNAs (*GAPDH*, *CDKN1A*, *PSMC4*, *18S rRNA*, *5S rRNA*) and (*C,F*) the additional three transcripts reported to be enriched in the nucleus (*GATA6*, *APBB2*, *SVIL*). Nucleic acids were extracted from 10 cells. "FD" and "FR" refer to genomic DNA and total RNA, respectively, fractionated by the SIDR method. The amount of DNA in "FR" and of RNA in "FD" indicates the amount of residual contamination in the counterpart fractions because of incomplete separation. The amounts of nucleic acids in each fraction were normalized to those in the whole cell lysates of 10 cells. Error bars represent the s.e.m.

Supplemental Figure S2

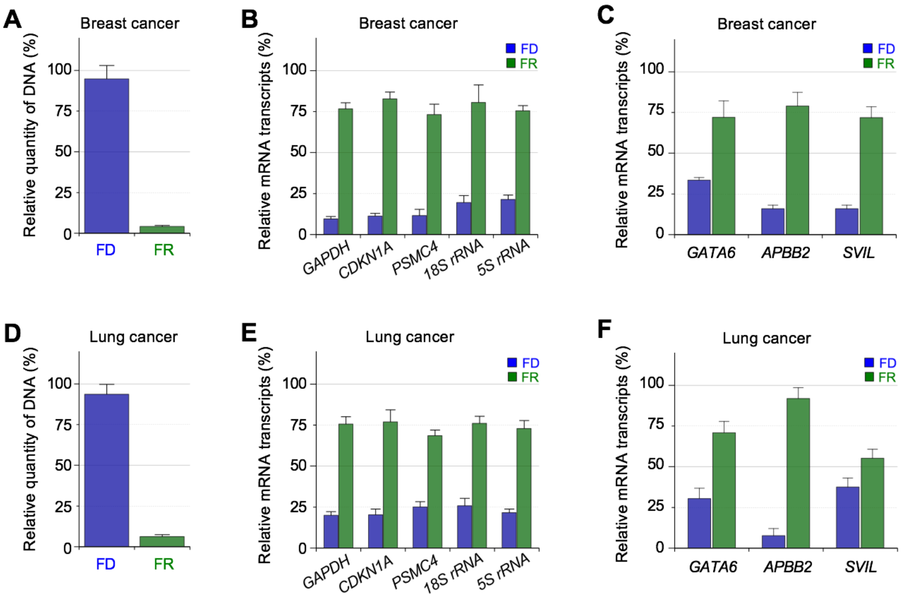

Supplemental Figure S2. Recovery rates of DNA (*A*,*D*) and RNA (*B*,C,*E*,*F*), by the SIDR method in breast cancer (*A*–*C*) and lung cancer (*D*–*F*) tissues. The efficiency of DNA recovery by the SIDR method was estimated by real-time PCR targeting the LINE-1 locus. The efficiency of RNA recovery by the SIDR method was estimated by real-time PCR targeting (*B*,*E*) cytoplasmic RNAs (*GAPDH*, *CDKN1A*, *PSMC4*, *18S rRNA*, *5S rRNA*) and (*C*,*F*) the additional three transcripts reported to be enriched in the nucleus (*GATA6*, *APBB2*, *SVIL*). Nucleic acids were extracted from 10 cells of breast cancer tissues (n = 2) and lung cancer tissues (n = 3). “FD” and “FR” refer to genomic DNA and total RNA, respectively, fractionated by the SIDR method. The amount of DNA in “FR” and of RNA in “FD” indicates the amount of residual contamination in the counterpart fractions because of incomplete separation. The amounts of nucleic acids in each fraction were normalized to those in the whole cell lysates of 10 cells. Error bars represent the s.e.m.

Supplemental Figure S3 (1/7)

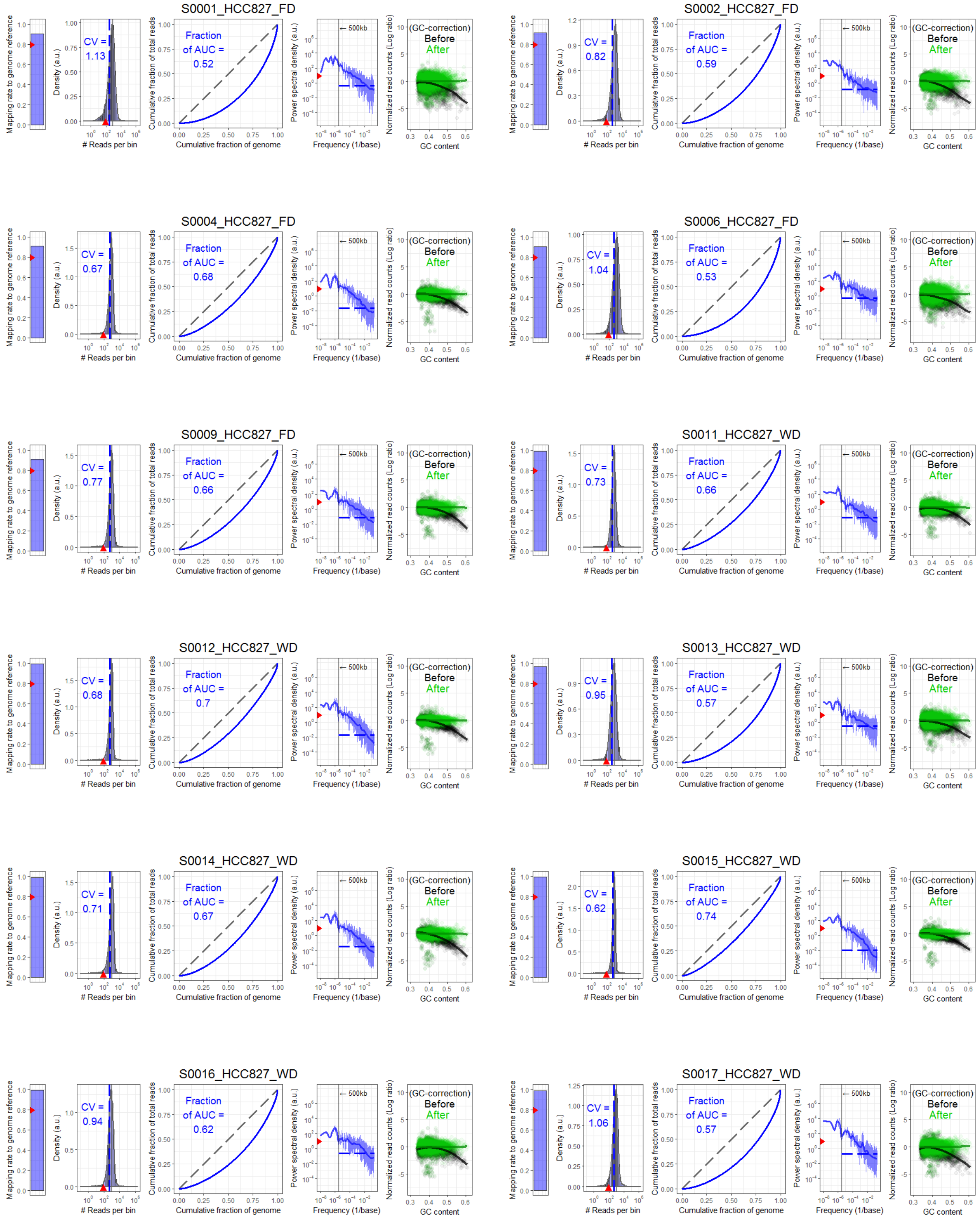

Supplemental Figure S3 (2/7)

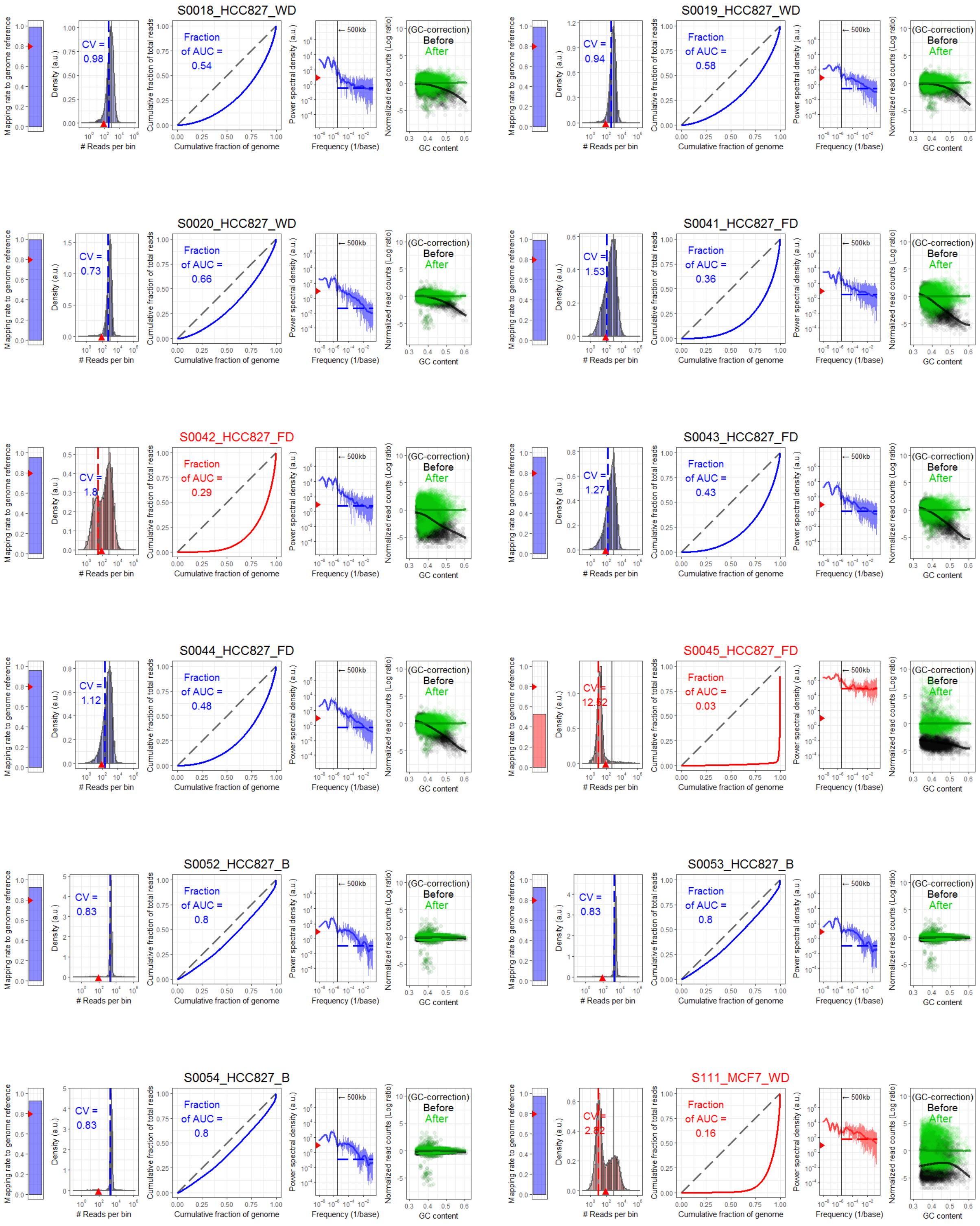

Supplemental Figure S3 (3/7)

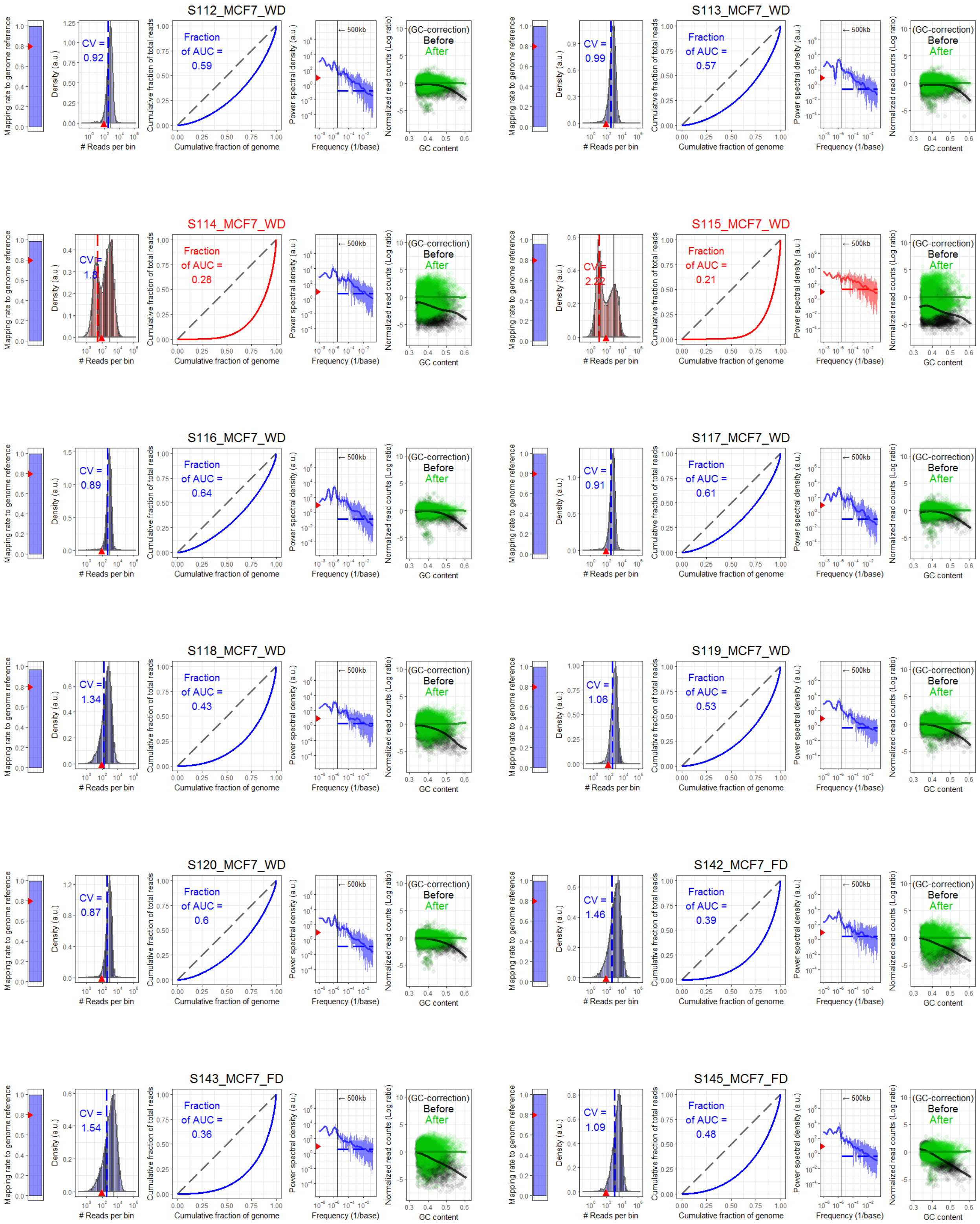

Supplemental Figure S3 (4/7)

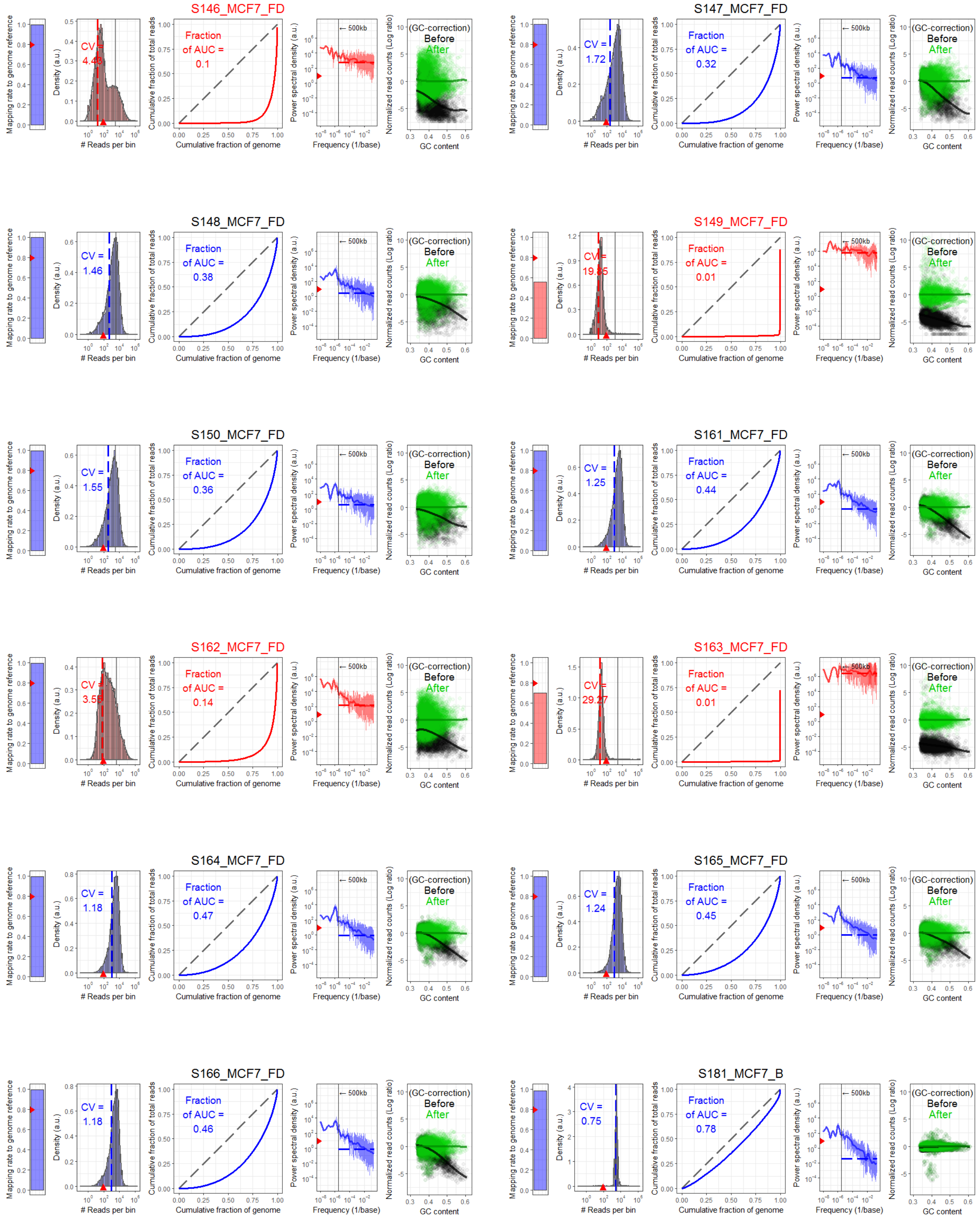

Supplemental Figure S3 (5/7)

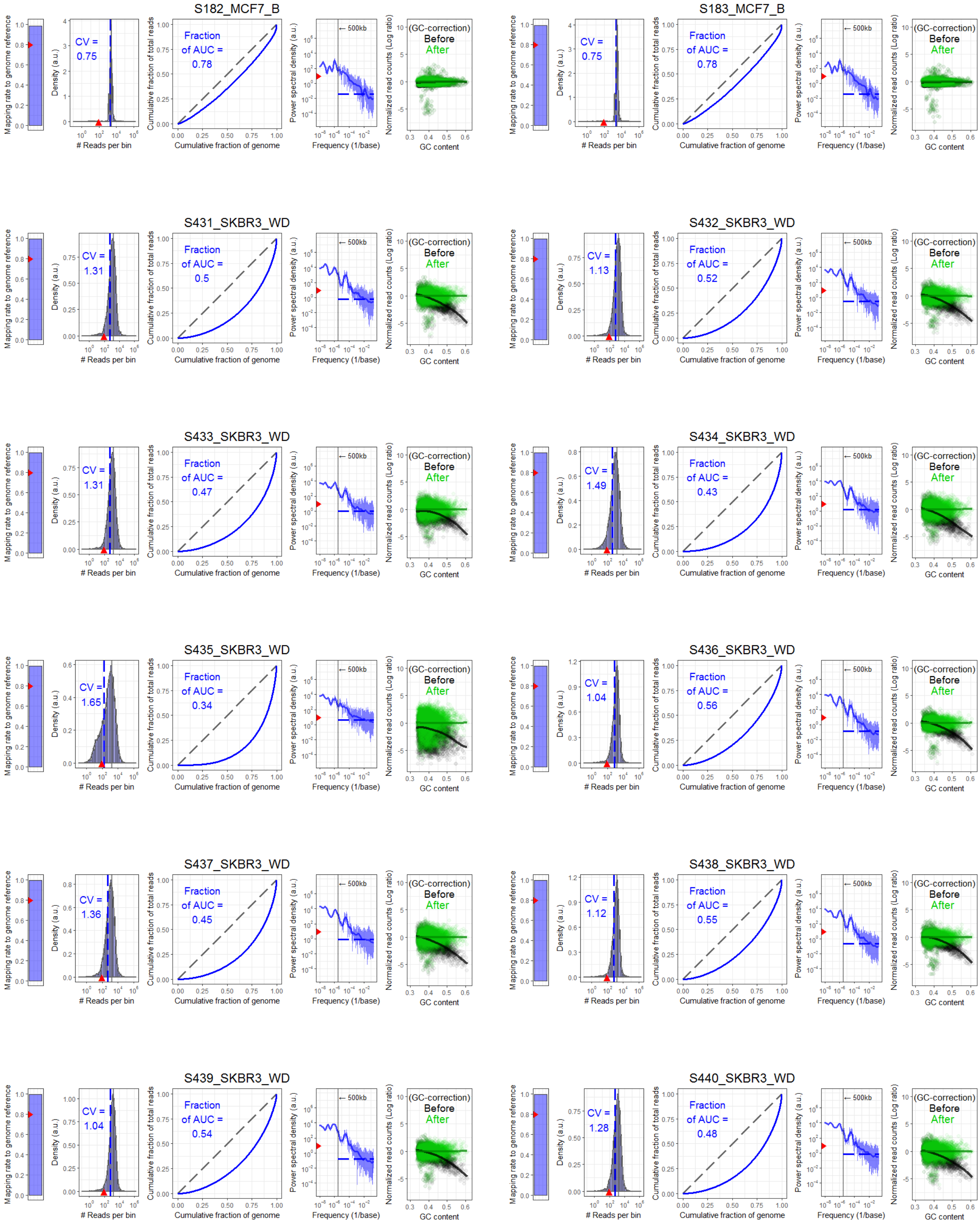

Supplemental Figure S3 (6/7)

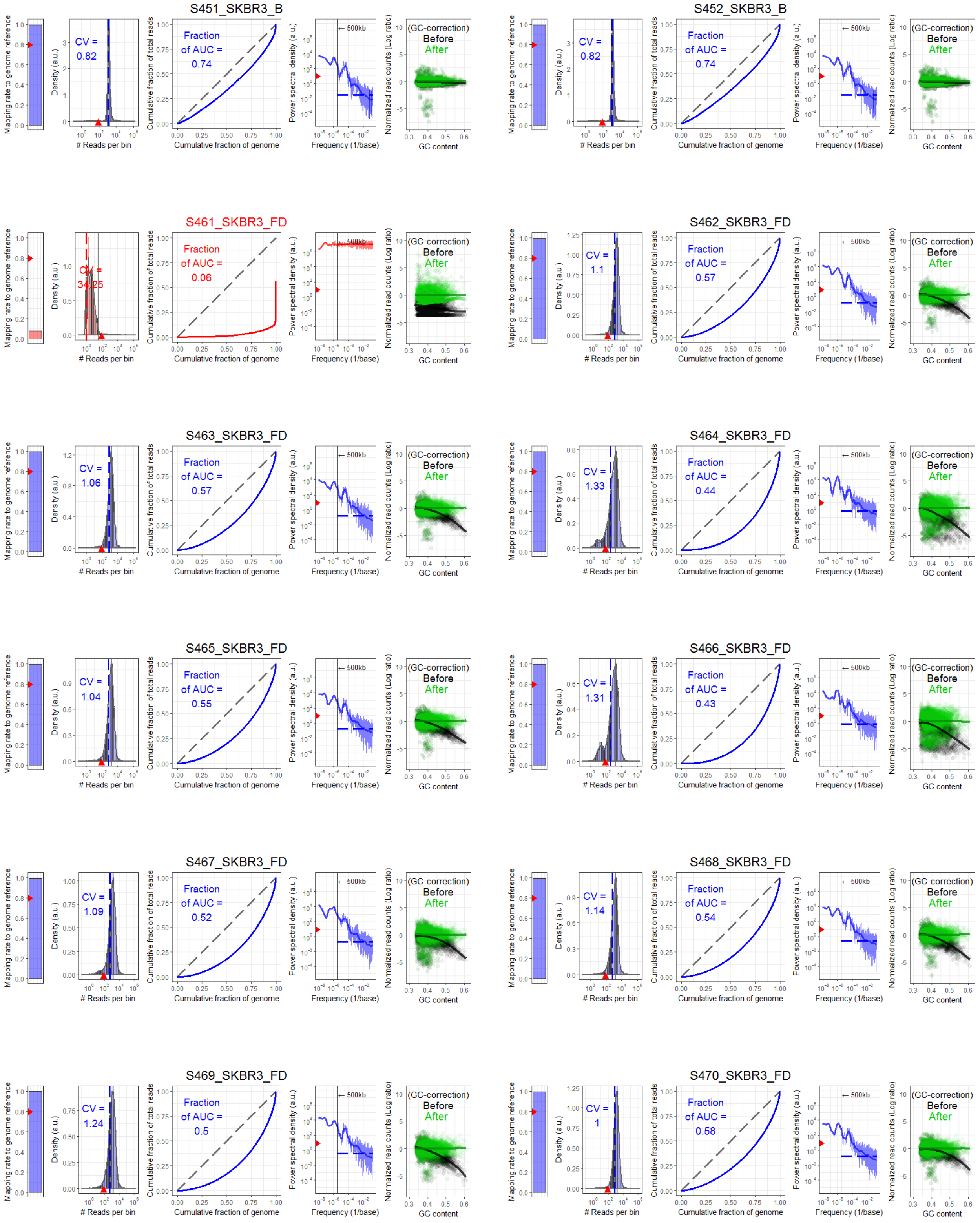

Supplemental Figure S3 (7/7)

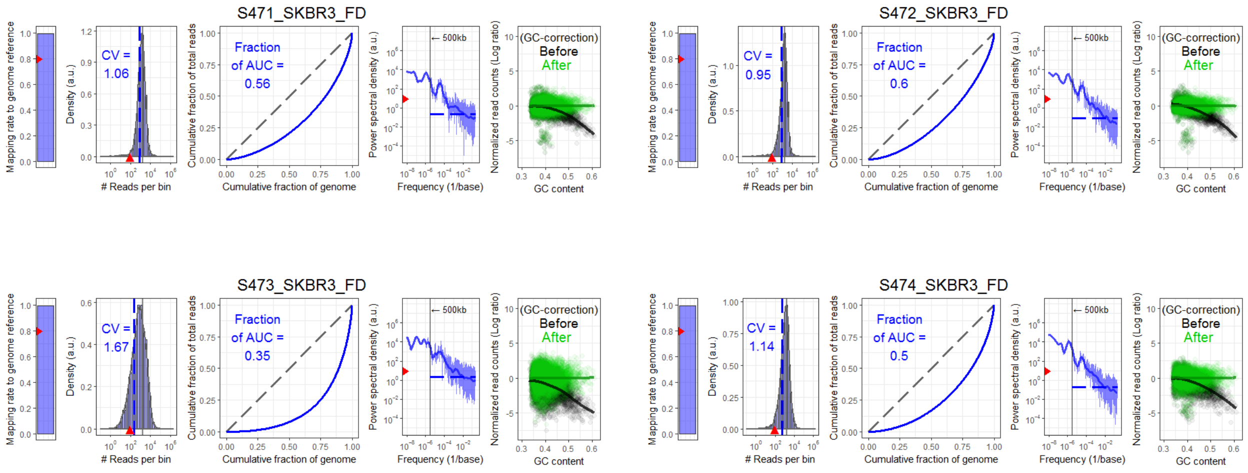

Supplemental Figure S3. Quality assessment of genome sequencing data. In total, 58/68 single cells (85%) passed QC criteria. Only sequencing data which met the following quality criteria were included in downstream analysis: 1) rate of uniquely mapping to the genome reference ≥0.8; 2) first quartile of bin-counts ≥100; 3) CV of bin-counts <2; 4) in Lorenz curves, fraction of area under the curve (AUC) to the perfect coverage uniformity ≥0.3; 5) in power spectral density curves, average values within genomic scale less than 500 kb <1.

Supplemental Figure S4

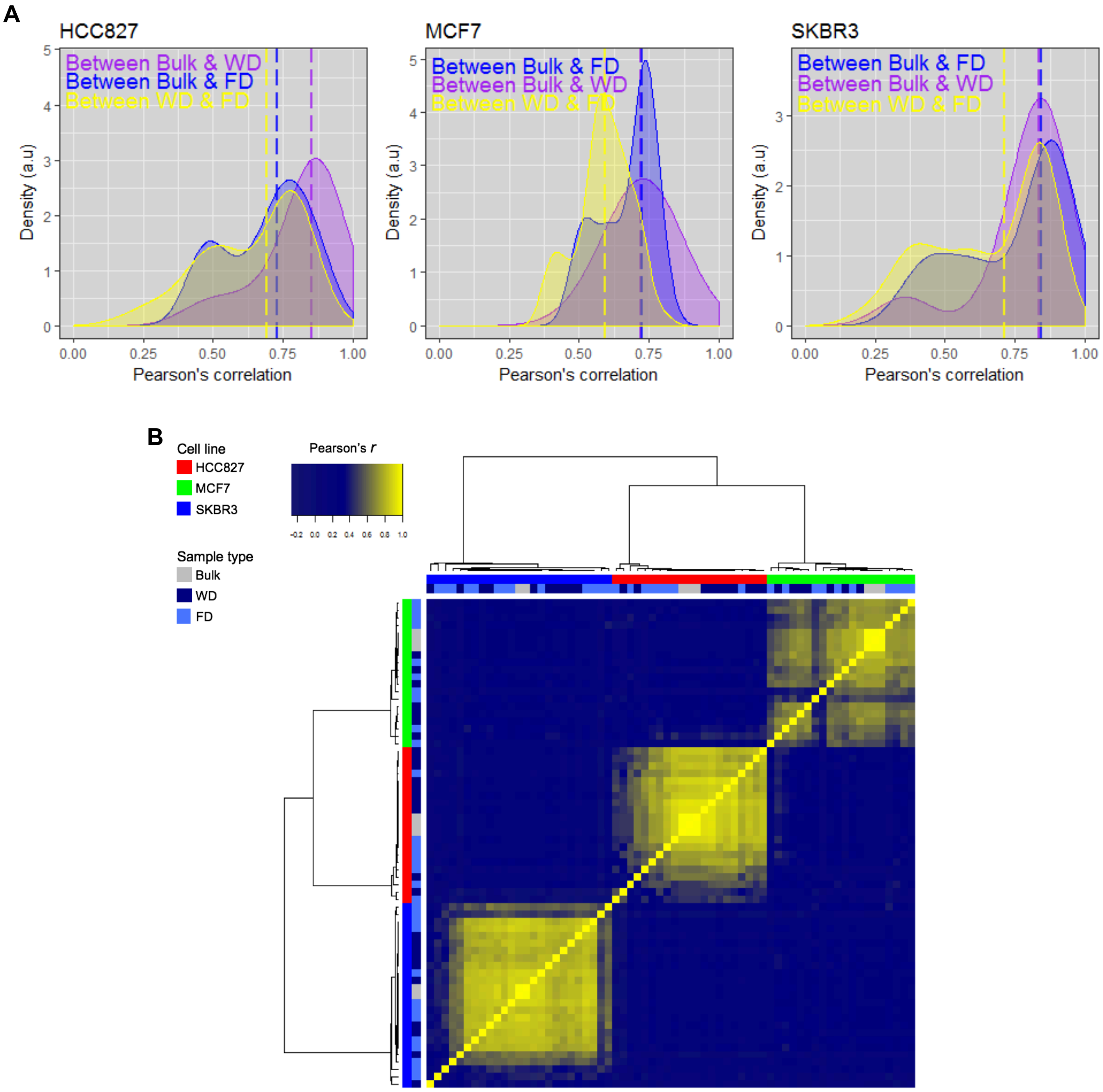

Supplemental Figure S4. Pairwise comparisons of copy-number profiles between bulk cells and single cells. (*A*) The distribution of Pearson's correlation coefficients of the pairwise comparisons. Dashed vertical lines indicate the median values. (*B*) Correlation heatmap for copy-number profiles based on genome sequencing data sets from three samples types (Bulk, WD and FD) out of three cell lines (HCC827, MCF7, and SKBR3). Dendrograms were generated using Ward's method.

Supplemental Figure S5

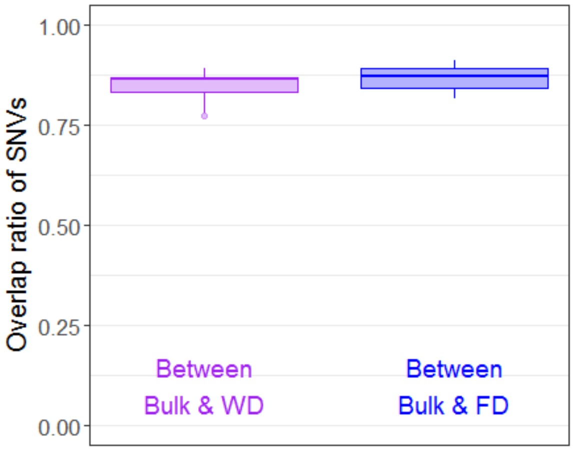

Supplemental Figure S5. The concordant fraction of single-cell SNVs validated in bulk cells. Among SNVs detected in MCF7 single-cell WES data, the fractions of the SNVs concordantly detected in bulk-cell WES data were plotted for WD and FD. Boxes show 25th and 75th percentile with 10th and 90th percentile whiskers.

Supplemental Figure S6

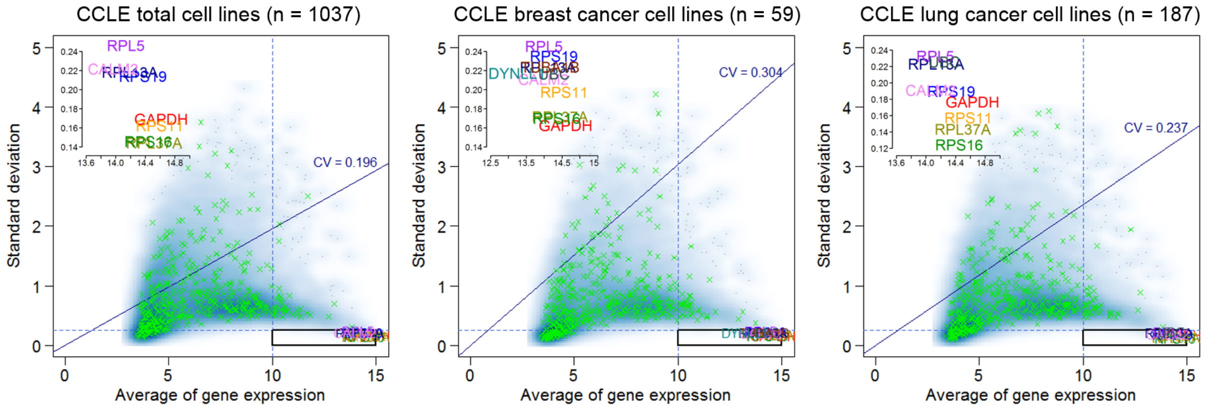

Supplemental Figure S6. Selection of constitutively expressed genes. Using the Cancer Cell Line Encyclopedia (CCLE) expression profiles, gene expression variabilities (standard deviation, y-axis) across the total (n = 1037), breast (n = 59) and lung (n = 187) cancer cell lines were plotted against their gene expression levels (x-axis). Housekeeping genes are marked with a green ×. The inset box at the lower right corner indicates genes that are highly expressed with a minimal variability (averaged gene expression >10 and standard deviation <0.25). Eight genes commonly found in all groups were selected: *RPL5*, *RPS19*, *RPL13A*, *RPL37A*, *RPS16*, *CALM2*, *GAPDH* and *RPS11*.

Supplemental Figure S7

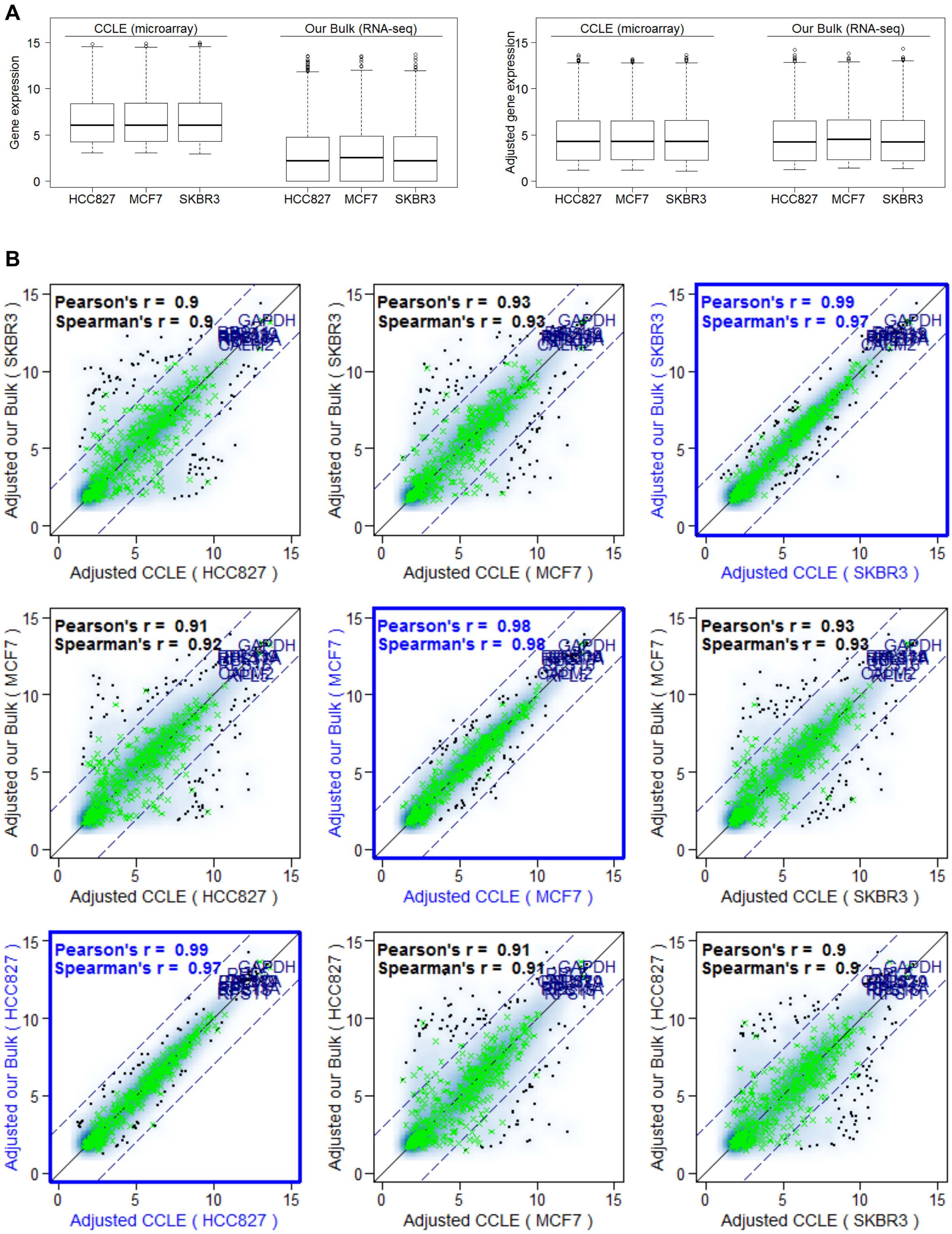

Supplemental Figure S7. Comparison of bulk RNA-seq data with CCLE microarray expression data. (*A*) Batch effect correction of gene expression data using the ComBat method. Distributions of Gene expression levels before and after the correction were box-plotted on the left and right panels, respectively. Boxes show 25th and 75th percentile with 10th and 90th percentile whiskers. (*B*) Pairwise comparison of gene expression levels between bulk RNA-seq data and CCLE data. The correlations increased between data pairs of matched cell lines (highlighted in thicker blue boxes). The eight constitutively expressed genes were highly correlated in all pairwise comparisons. Housekeeping genes were marked as green ×. Dashed diagonal lines represent the three fold-change thresholds in $\log_2$ ratio between two gene expression profiles.

Supplemental Figure S8 (1/6)

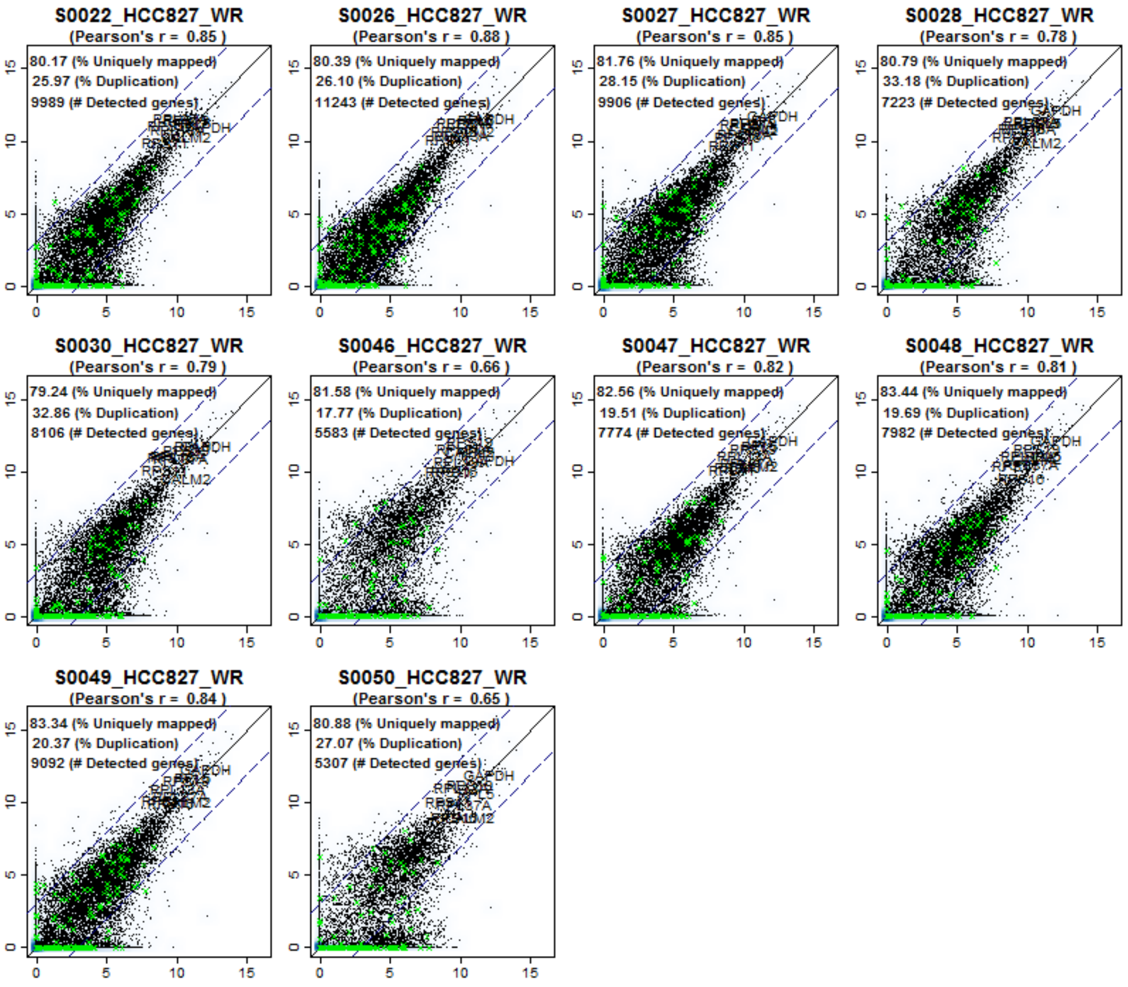

Supplemental Figure S8 (2/6)

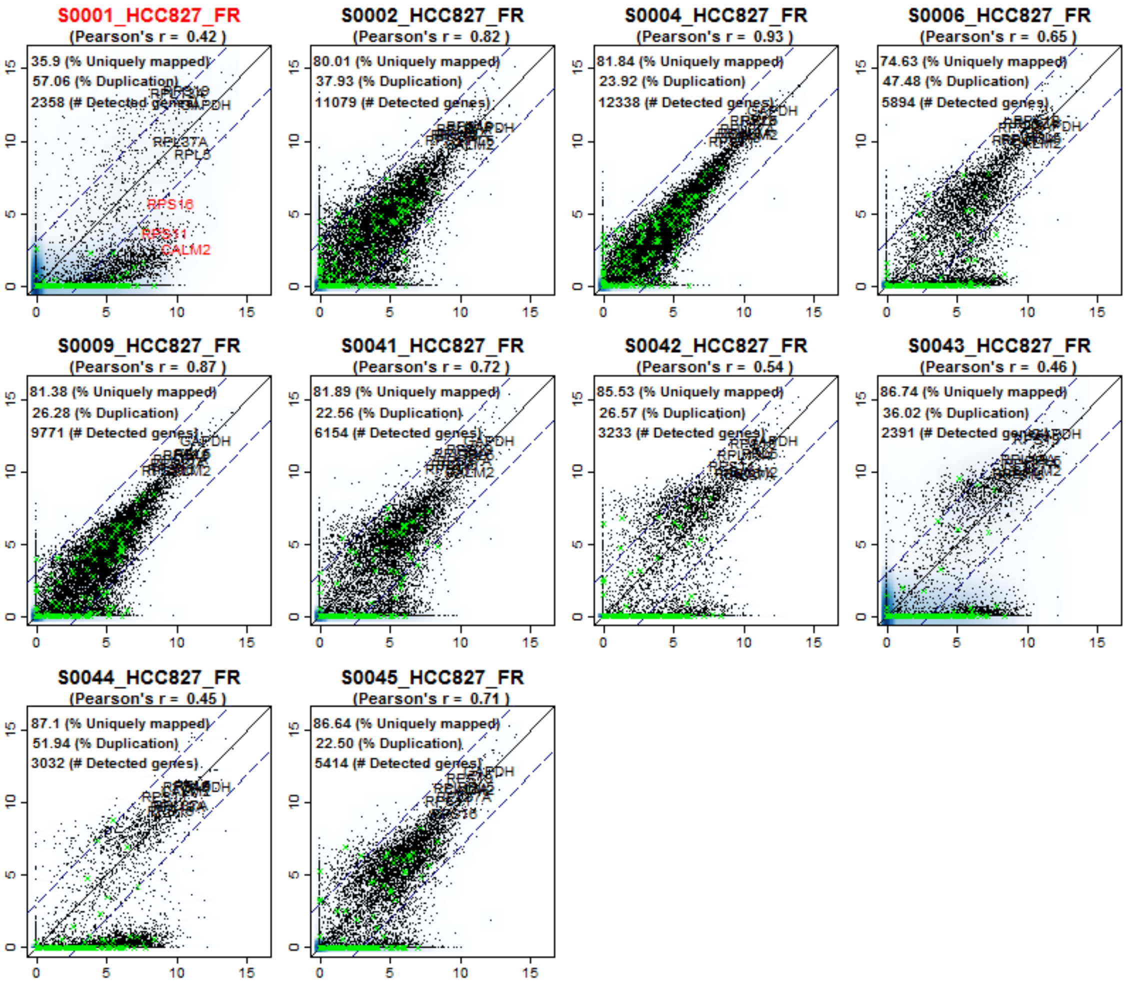

Supplemental Figure S8 (3/6)

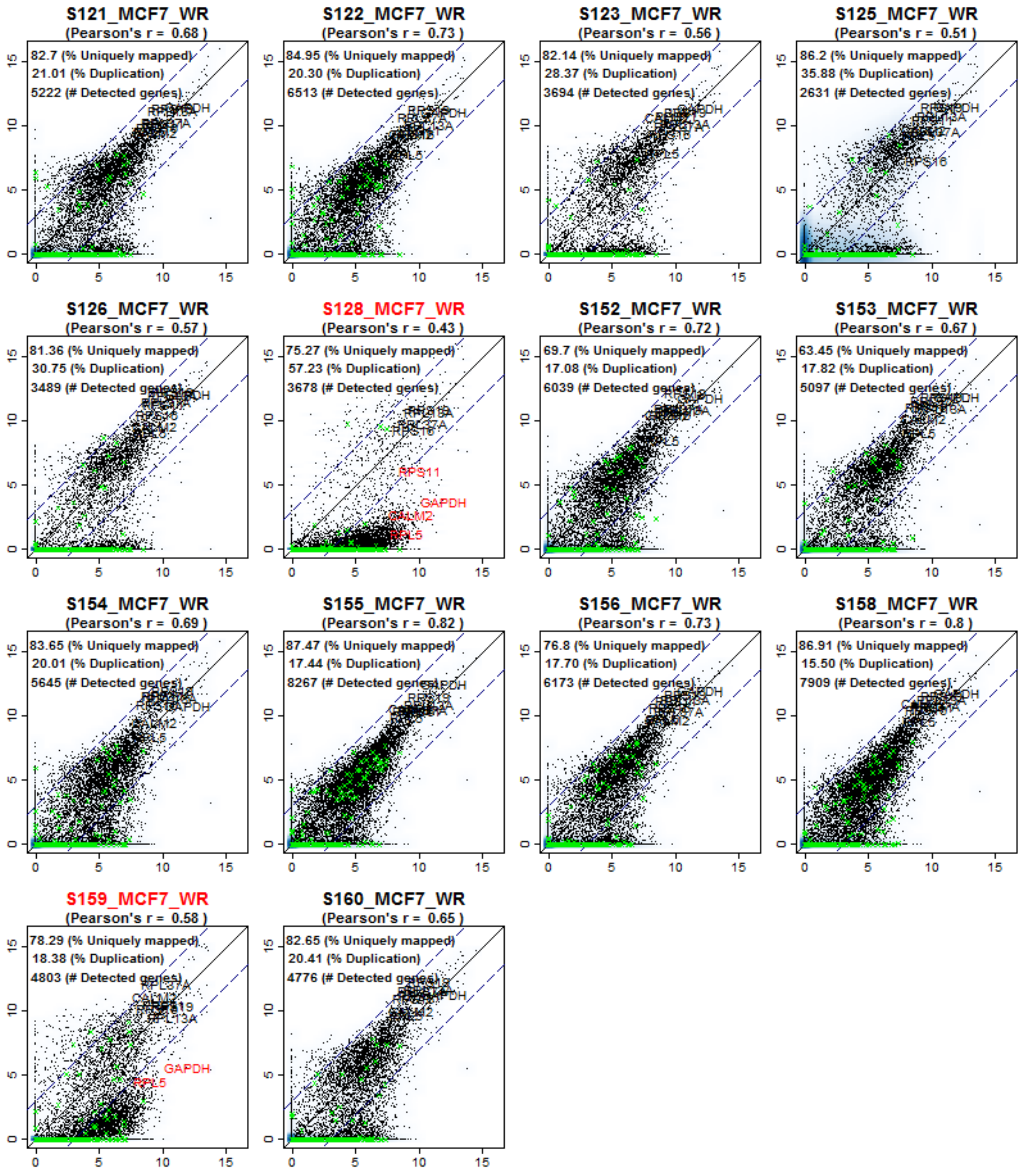

Supplemental Figure S8 (4/6)

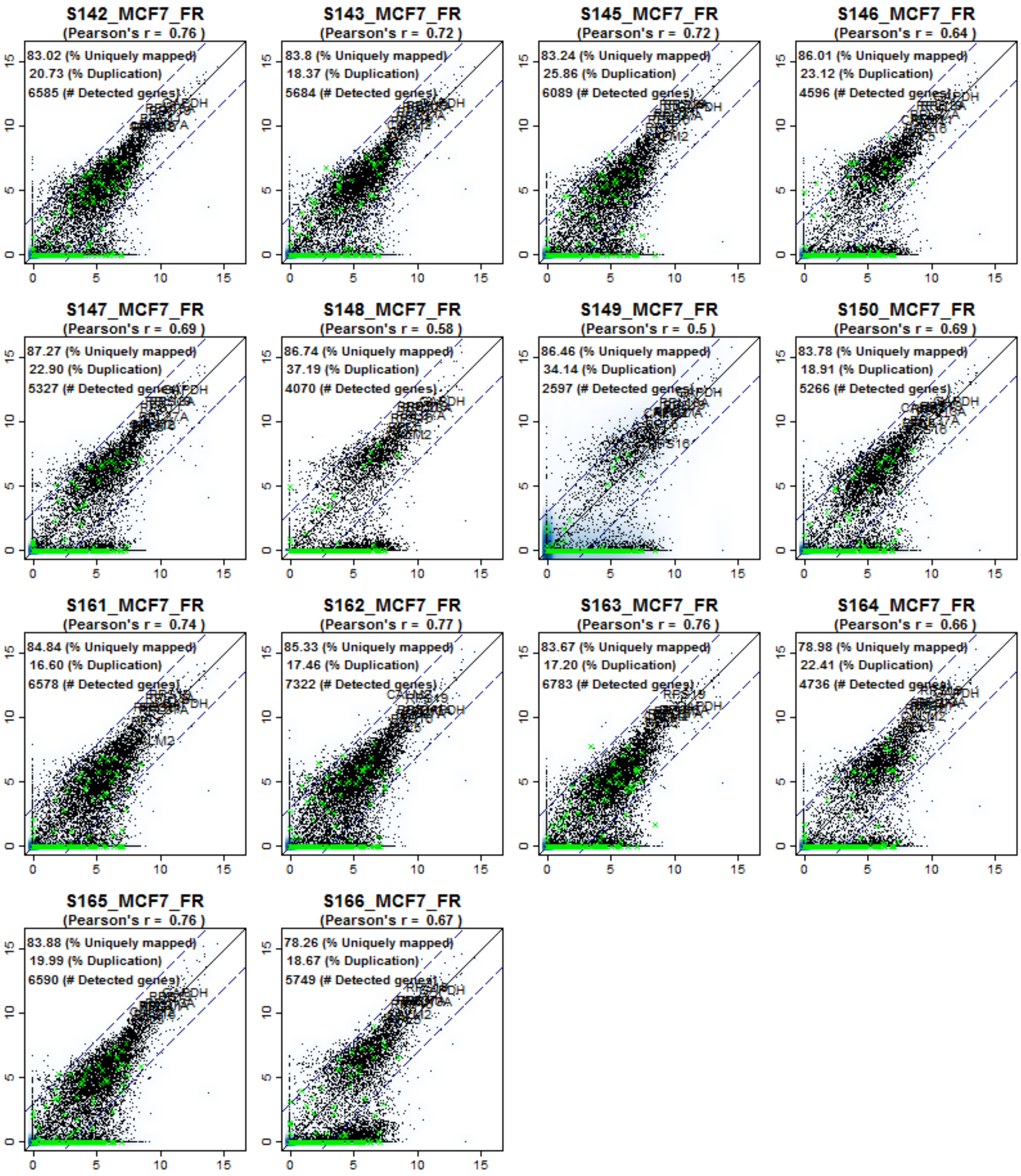

Supplemental Figure S8 (5/6)

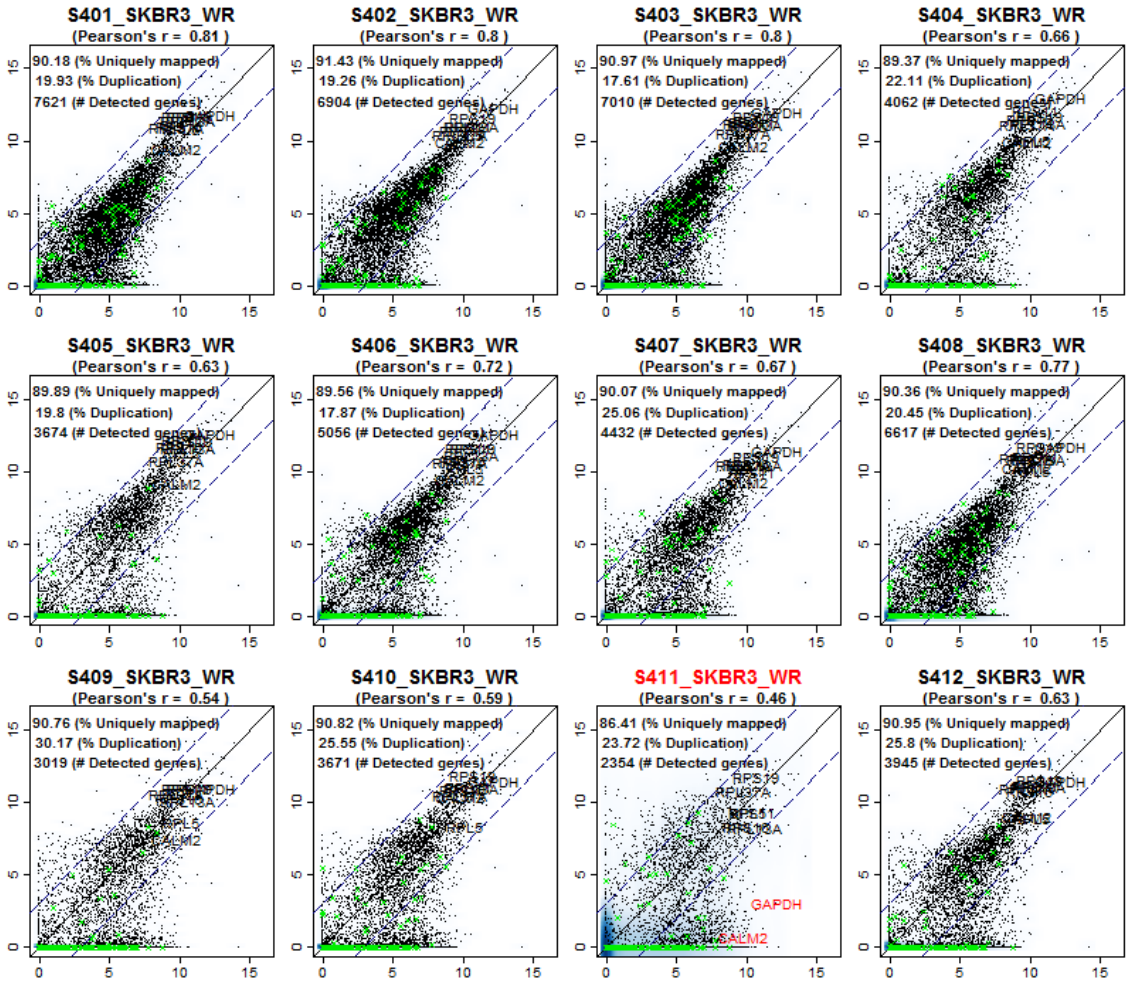

Supplemental Figure S8 (6/6)

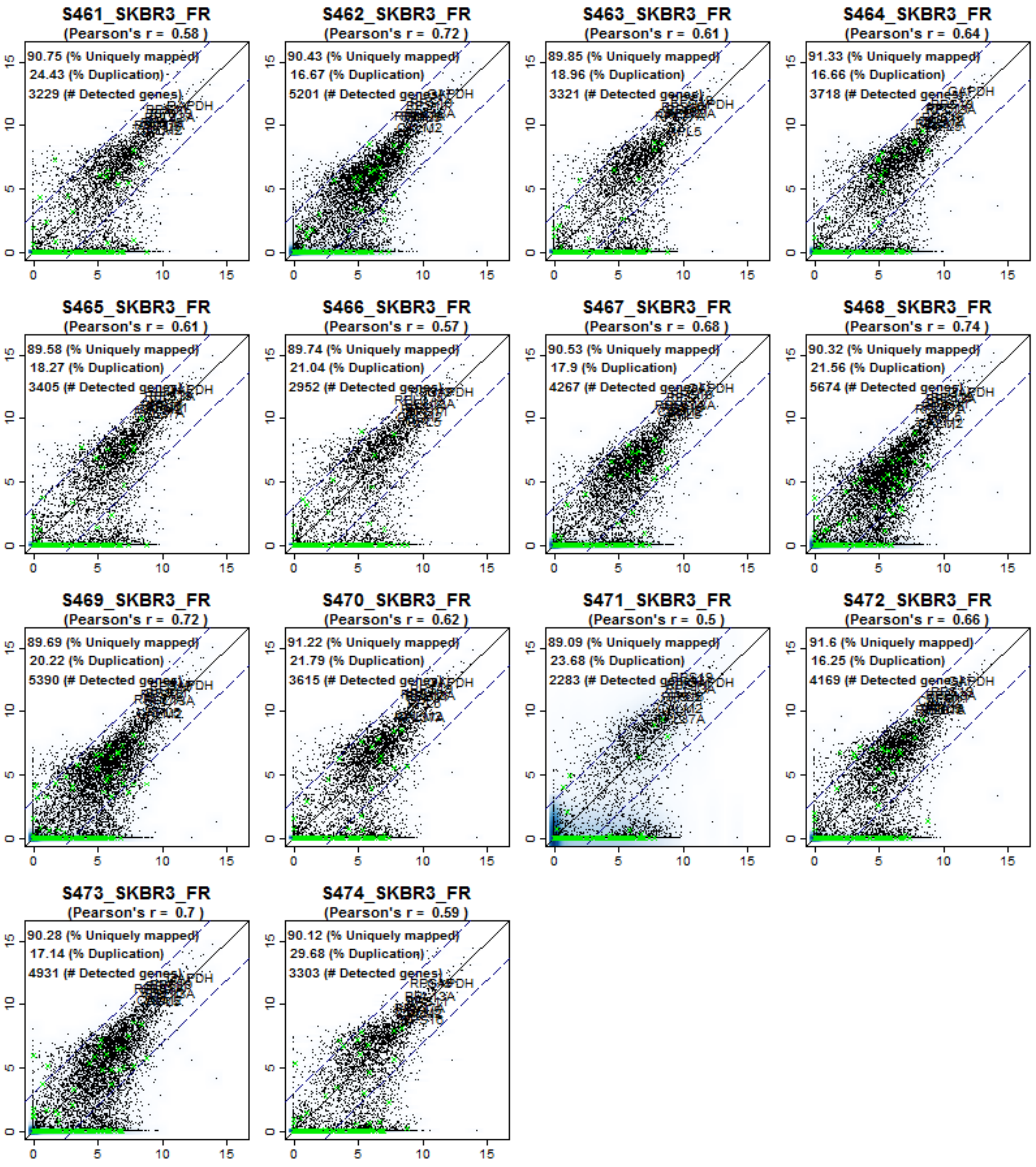

Supplemental Figure S8. Quality assessment of RNA-seq data. Gene expression levels between single cell RNA-seq data (*y*-axis) and bulk RNA-seq data (*x*-axis) were compared. Using the eight constitutively expressed genes identified in Supplemental Fig. S6, we filtered out poor quality samples (labelled in red) when log_2 ratio of these genes differed more than three-fold (highlighted with red out of the dashed lines). In total, 70/74 single cells (95%) passed QC criteria. We observed that poor quality cells showing at least 3-fold change in gene expression of these markers also had lower correlation to corresponding bulk cells, a lower rate of unique mapping, a higher rate of duplicated reads and lower number of detected genes.

Supplemental Figure S9

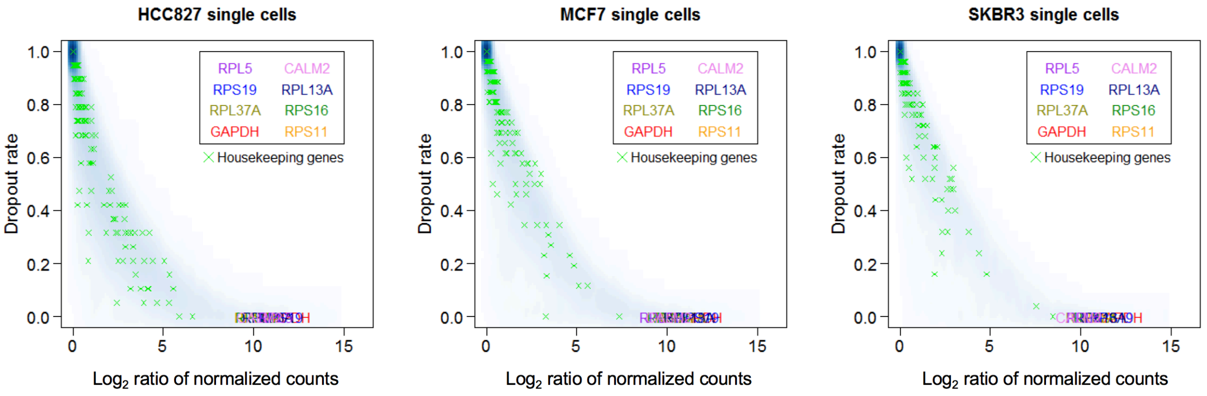

Supplemental Figure S9. The relationship between dropout rate (i.e. fraction of cells in which a gene of interest was not detectable) and mean non-zero expression level of housekeeping genes. The eight housekeeping genes with little variability (Supplemental Fig. S6) are marked in color. Housekeeping genes are marked with a green ×.

Supplemental Figure S10

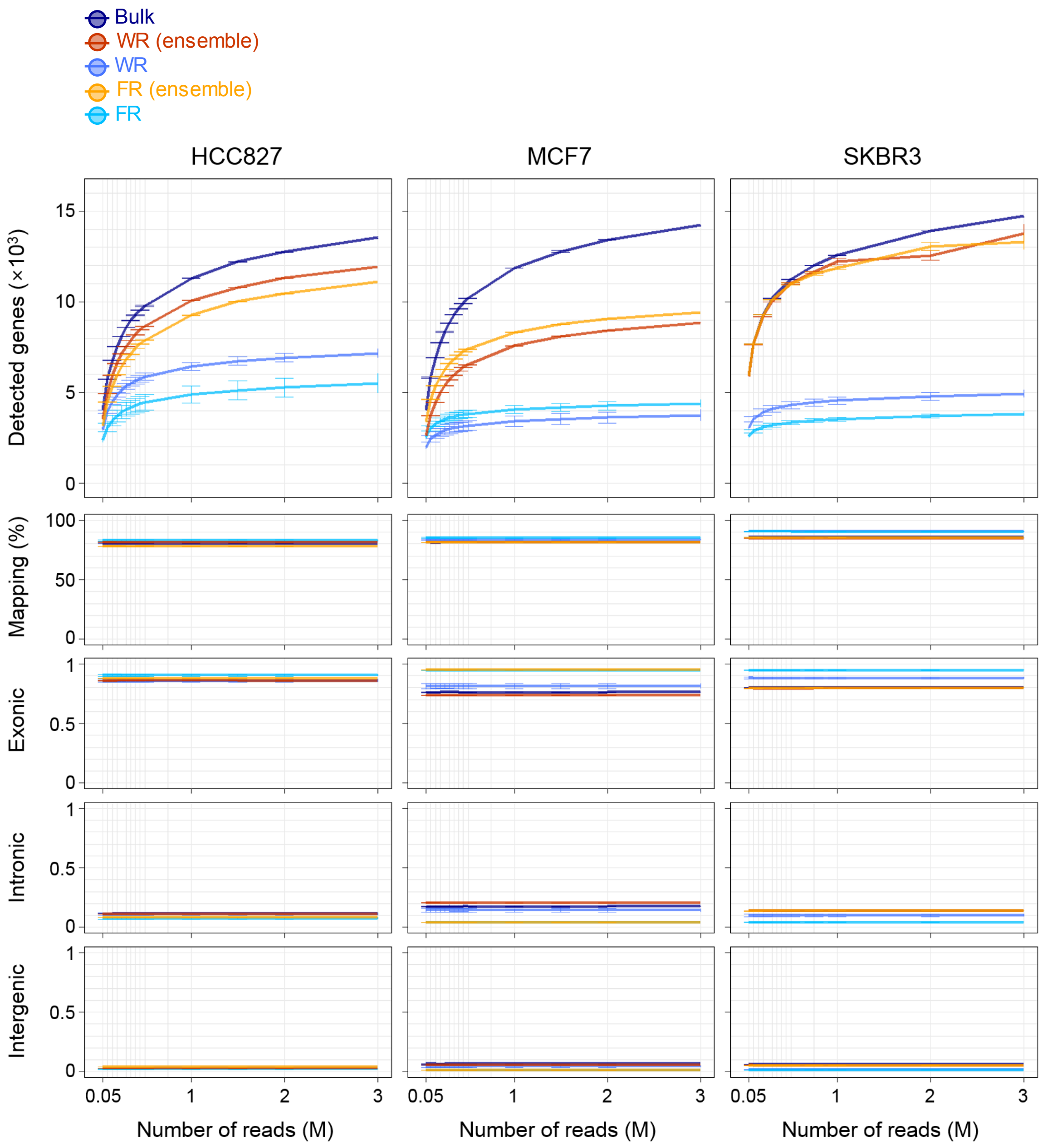

Supplemental Figure S10. Quality control metrics of RNA sequencing with increasing numbers of reads. The number of unique genes detected at a given number of raw reads. Raw reads as indicated on *x*-axis (0.05, 0.1, 0.15, 0.2, 0.3, 0.4, 0.5, 0.75, 1, 2 and 3 million) were randomly selected (in quintuplicate) in each sample library. For the construction of the merged single cell data sets (ensemble), raw reads from all single cell libraries (both single cell fractions and whole single cells) were pooled and analyzed to mimic the bulk cell library. Error bars represent the s.e.m.

Supplemental Figure S11

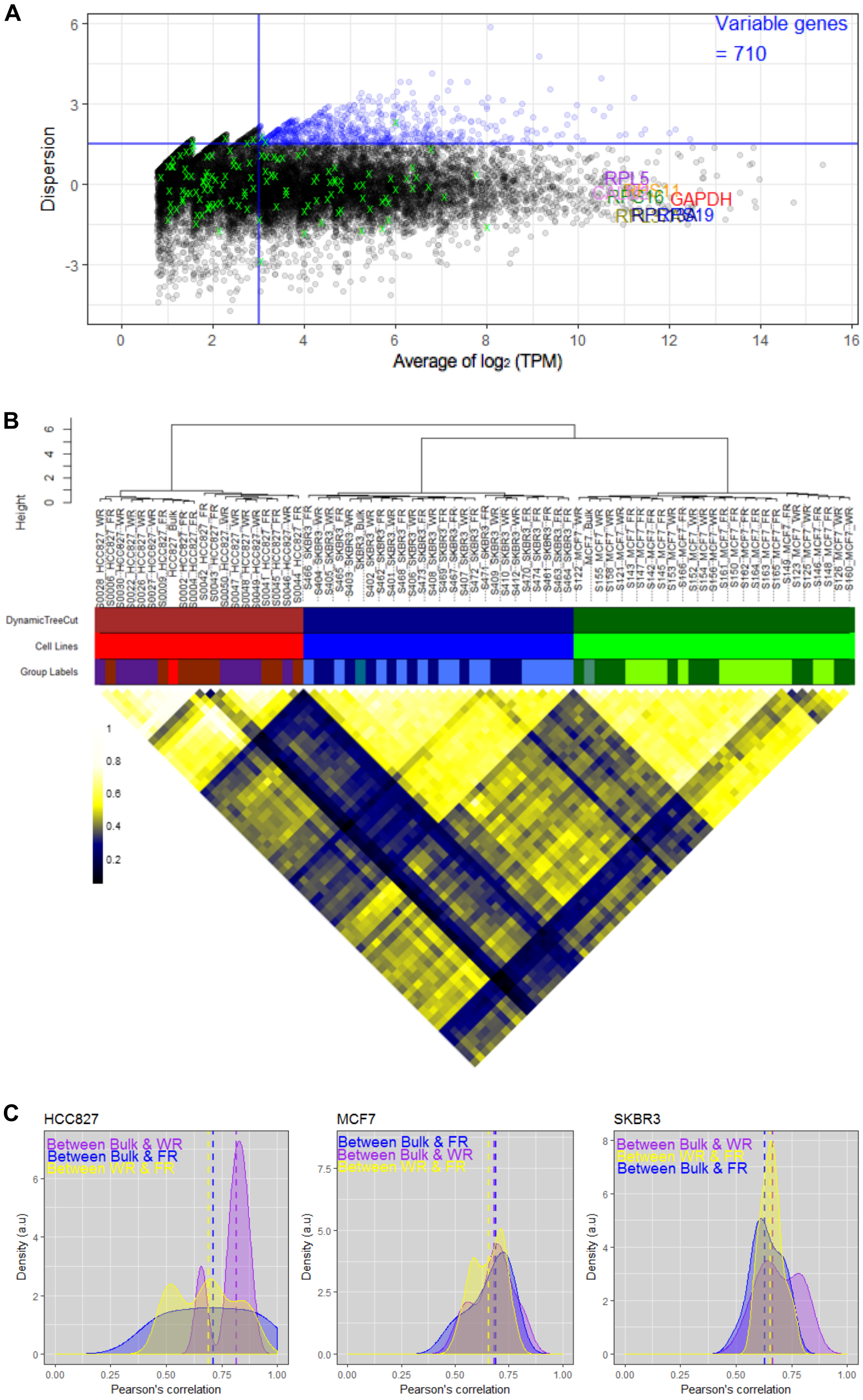

Supplemental Figure S11. Identification of cell line-specific gene expression profiles. (*A*) Selection of genes displaying high cell-to-cell variability in their expression levels. Two-dimensional scatterplot shows 710 variable genes, applying the cutoff of average expression ($\log_2$(TPM + 1)) >3 and dispersion >1.5. Housekeeping genes are marked with a green ×. The eight housekeeping genes with little variability (Supplemental Fig. S6) are marked in color. (*B*) Unsupervised hierarchical clustering of the expression profiles of the variable genes was performed, applying Pearson's distance with Ward's method. Pairwise correlation coefficients (Pearson's) are shown in the heatmap of lower panel. (*C*) The distribution of correlation coefficients of the gene expression data between RNA-seq data sets. Dashed vertical lines indicate the median values.

Supplemental Figure S12

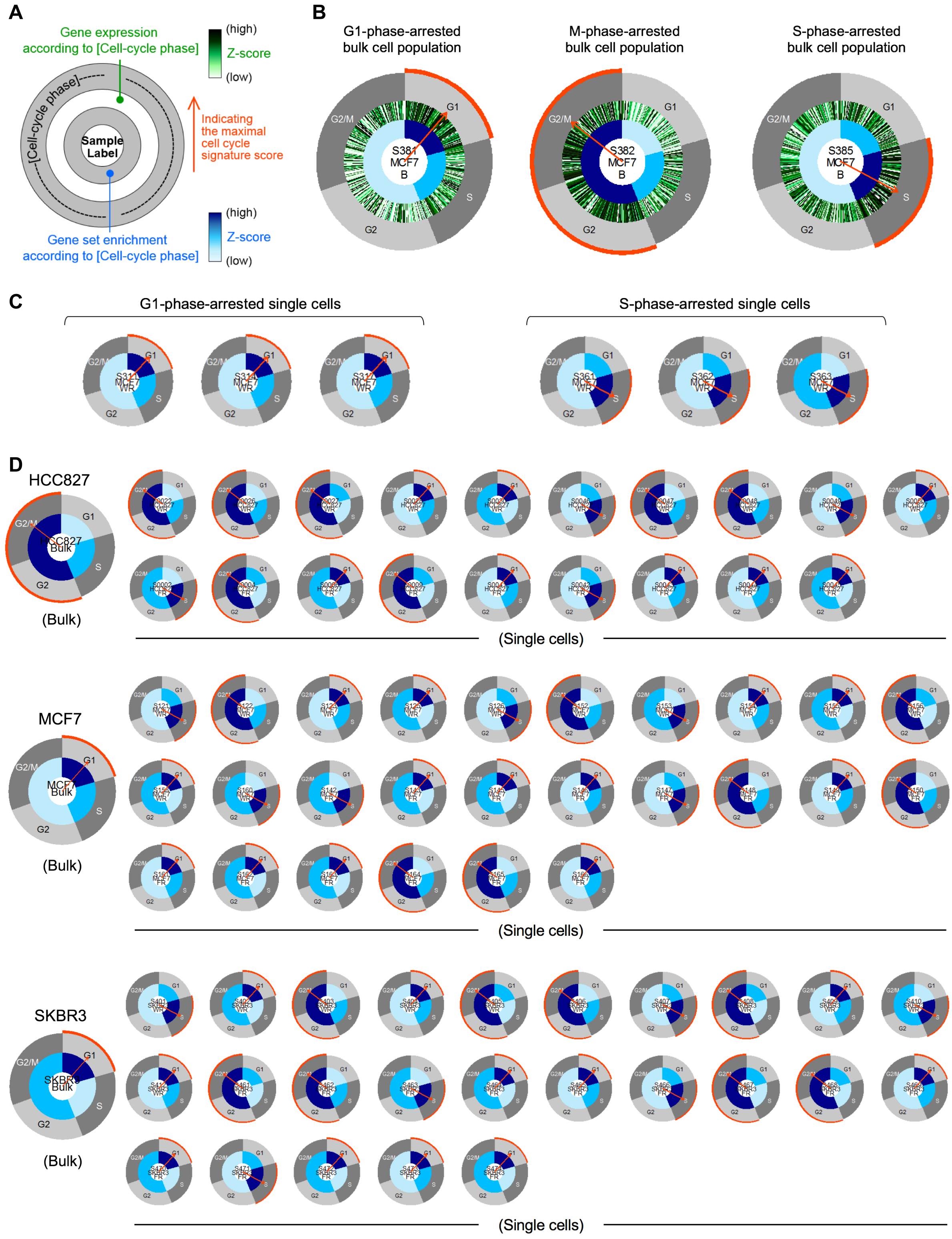

Supplemental Figure S12. Analysis of the cell cycle based on phase-specific gene expression. (*A*) Schematic drawing explaining data presentation in the circular chart. (*B*) Representation of cell-cycle-related gene expression of bulk cells. Genes with a consistent pattern of periodic expression reported in previous work were selected (Whitfield et al. 2002). Cells in G1, S, and M phases were isolated by fluorescence activated cell sorting (FACS) cytometry. The highest expression level of Z-scores among cell-cycle phases is indicated with an orange arrow. (*C*) Validation of cell-cycle inference based on marker gene expression using G1 and S phase single cells isolated by FACS. (*D*) Estimation of cell cycle based on cell cycle-related gene expression.

Supplemental Figure S13

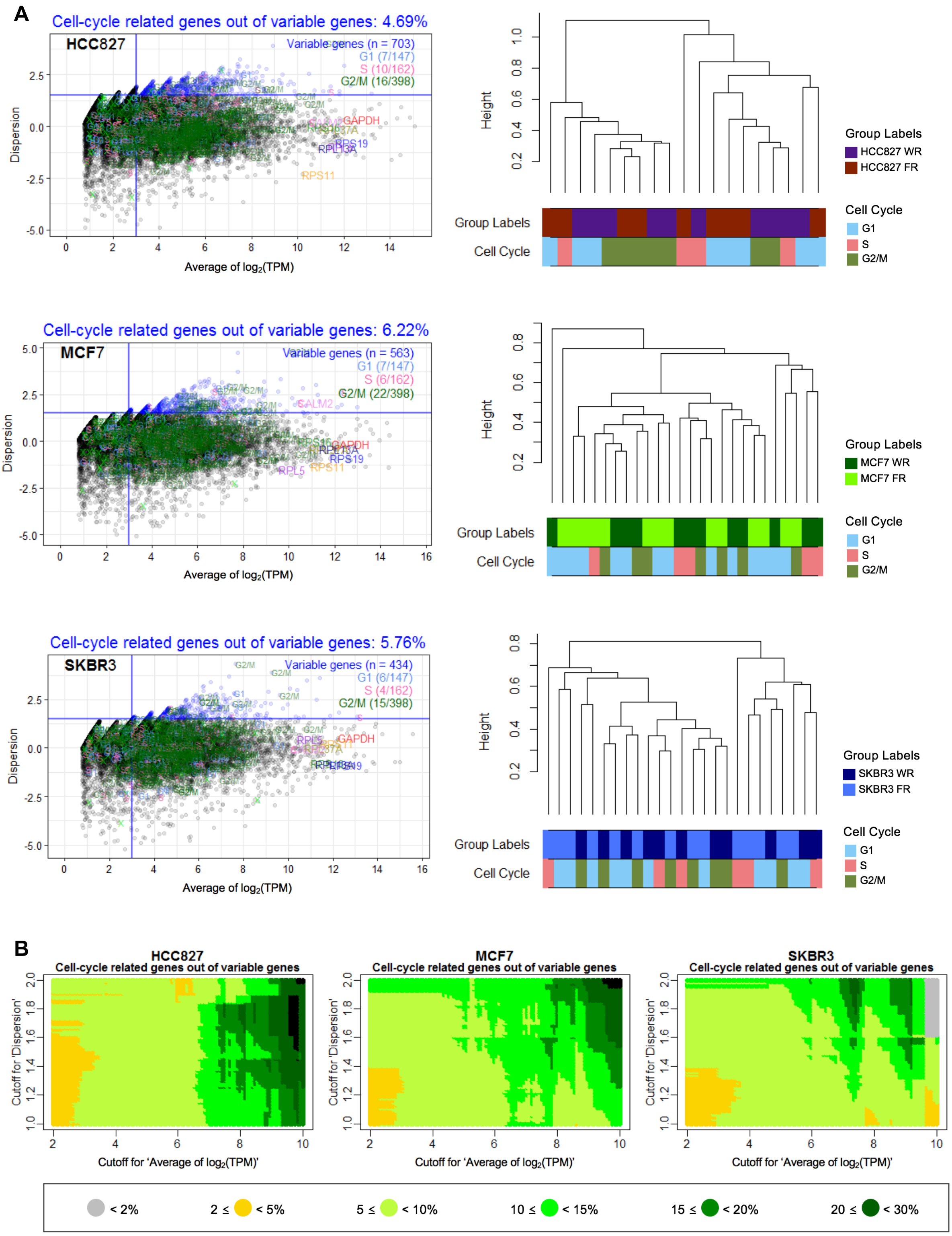

Supplemental Figure S13. Contribution of cell-cycle related genes to cell-to-cell heterogeneity. (*A*) Two-dimensional scatterplots to visualize gene expression levels and their cell-to-cell variability. The fractions of the variable genes related to the cell-cycle are displayed at the top of the right panels, applying the cutoff of average expression ($\log_2$(TPM + 1)) >3 and dispersion >1.5. Unsupervised hierarchical clustering of the variable gene expression profiles was performed for WRs and FRs of different cell cycles as shown on the left. The dendrograms were generated using Ward's method and Pearson's distance. (*B*) Investigation of how the fraction of variable genes identified as cell-cycle related changes with different cutoffs of average expression and dispersion. Heatmaps representing the fractions of cell-cycle related variable genes depending on their cut-off values are shown; gene expression levels and degree of cell-to-cell variability are included. The colors of circles represent the fractions of genes identified as cell-cycle related.

Supplemental Figure S14 (1/3)

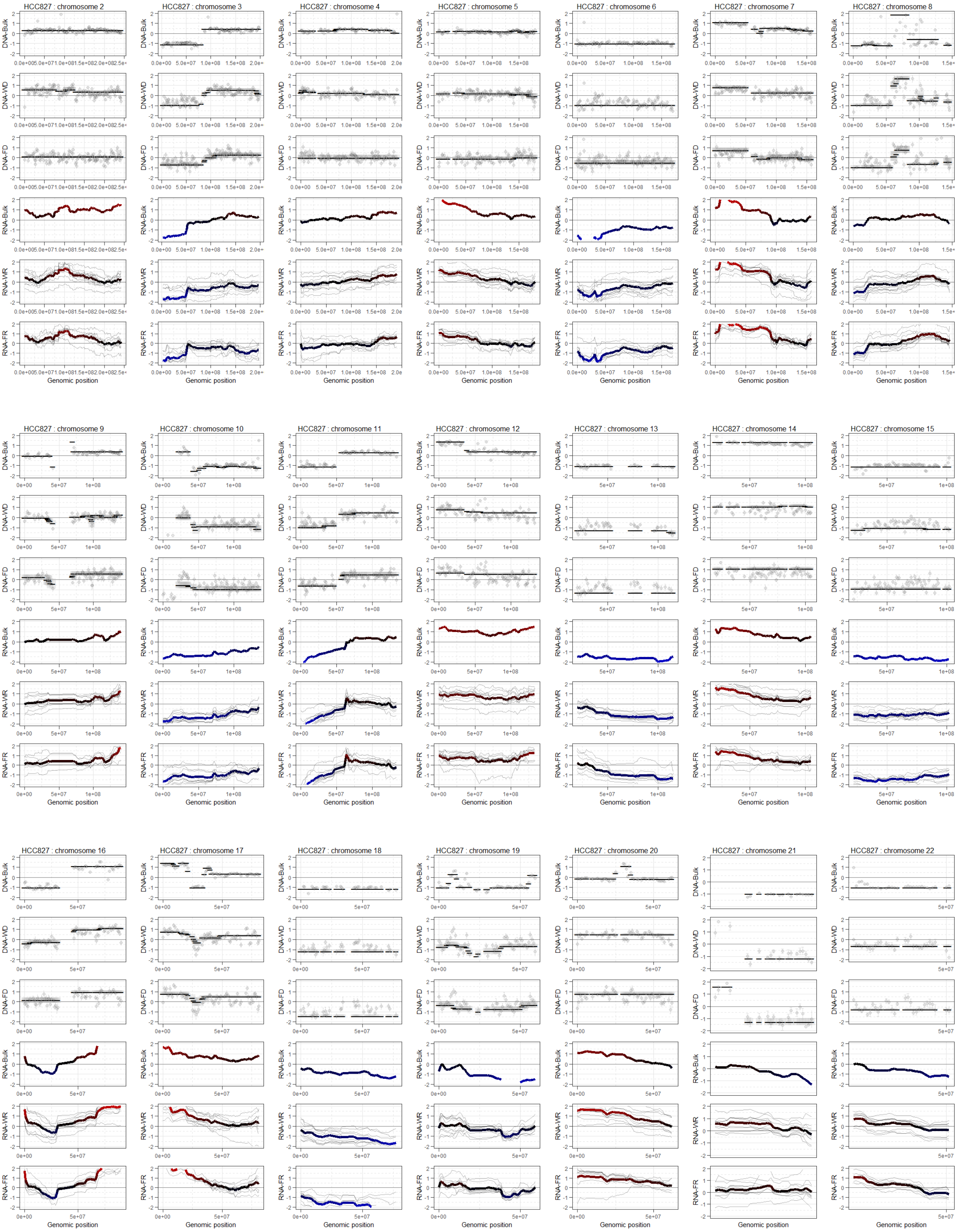

Supplemental Figure S14 (2/3)

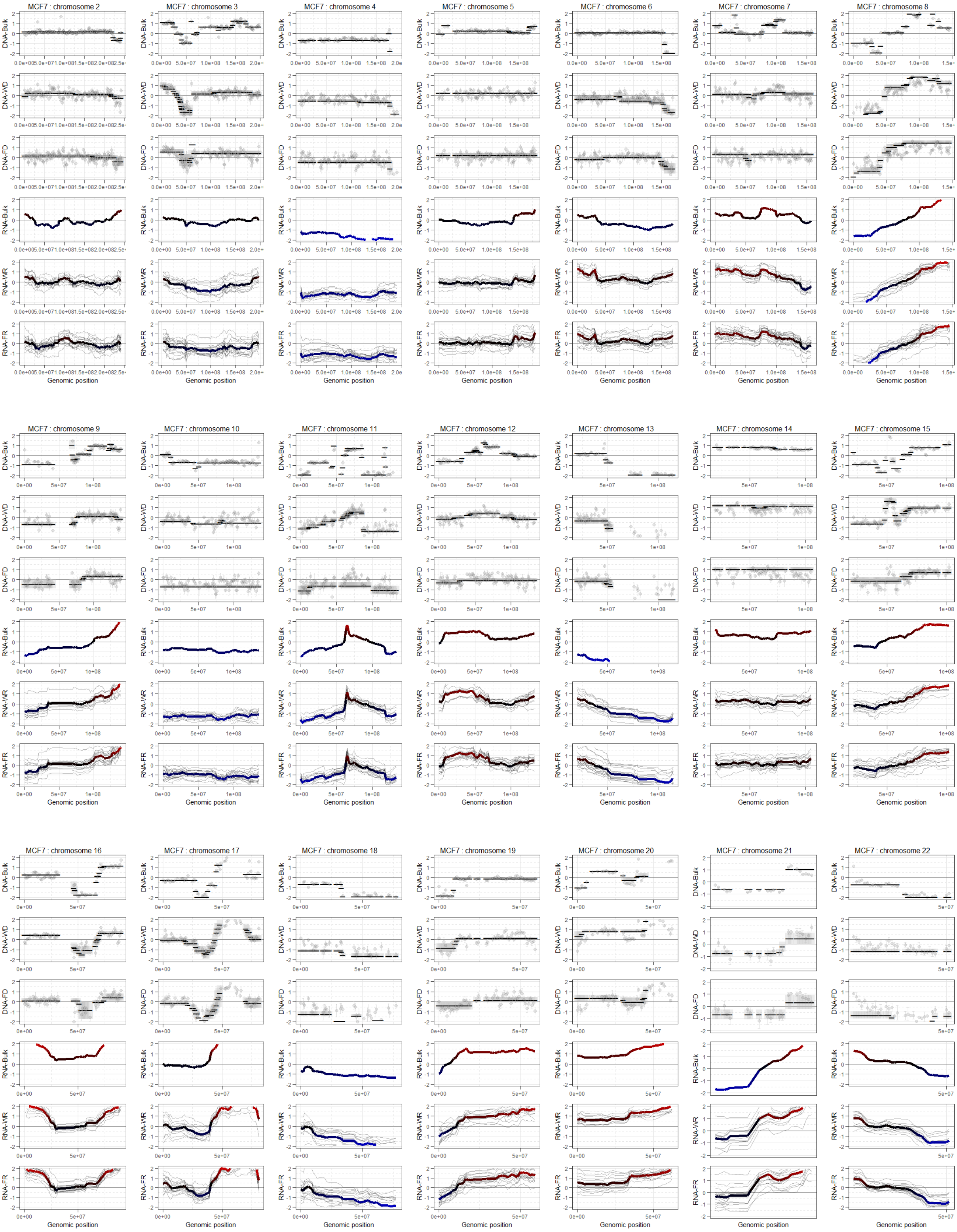

Supplemental Figure S14 (3/3)

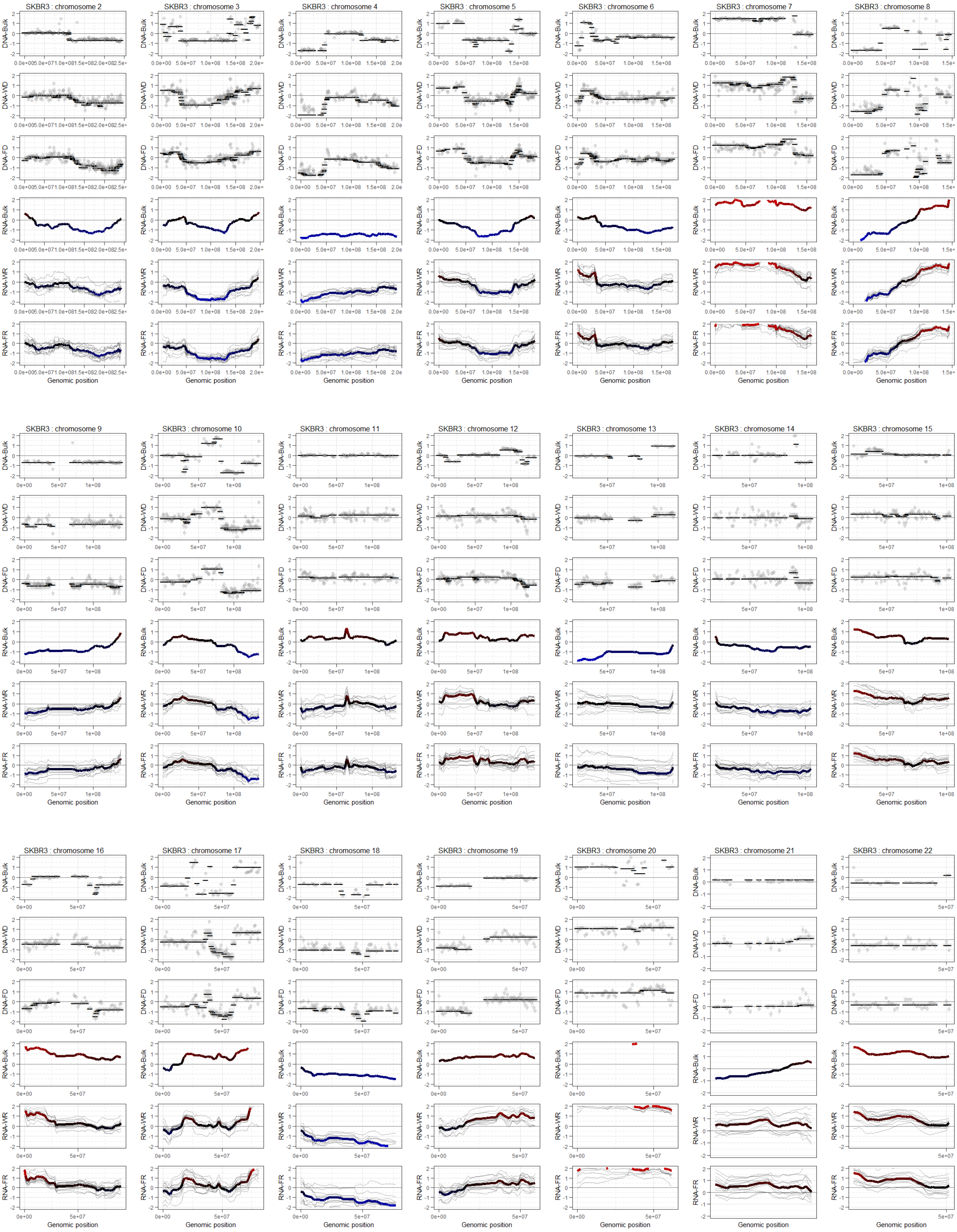

Supplemental Figure S14. Comparison of genomic copy-numbers and chromosomal gene expression patterns for each chromosome. The plot for chromosome 1 is available in Fig. 5B. In the upper three plots, the log_2 ratio of genomic copy-numbers (dots) and their CBS-derived segmented values (black lines) estimated from bulk and single cells for DNA sequencing are shown. The lower three plots show the chromosomal gene expression values (Z-score) from bulk and single cells (each in thinner gray line and their average in colored line).

Supplemental Figure S15

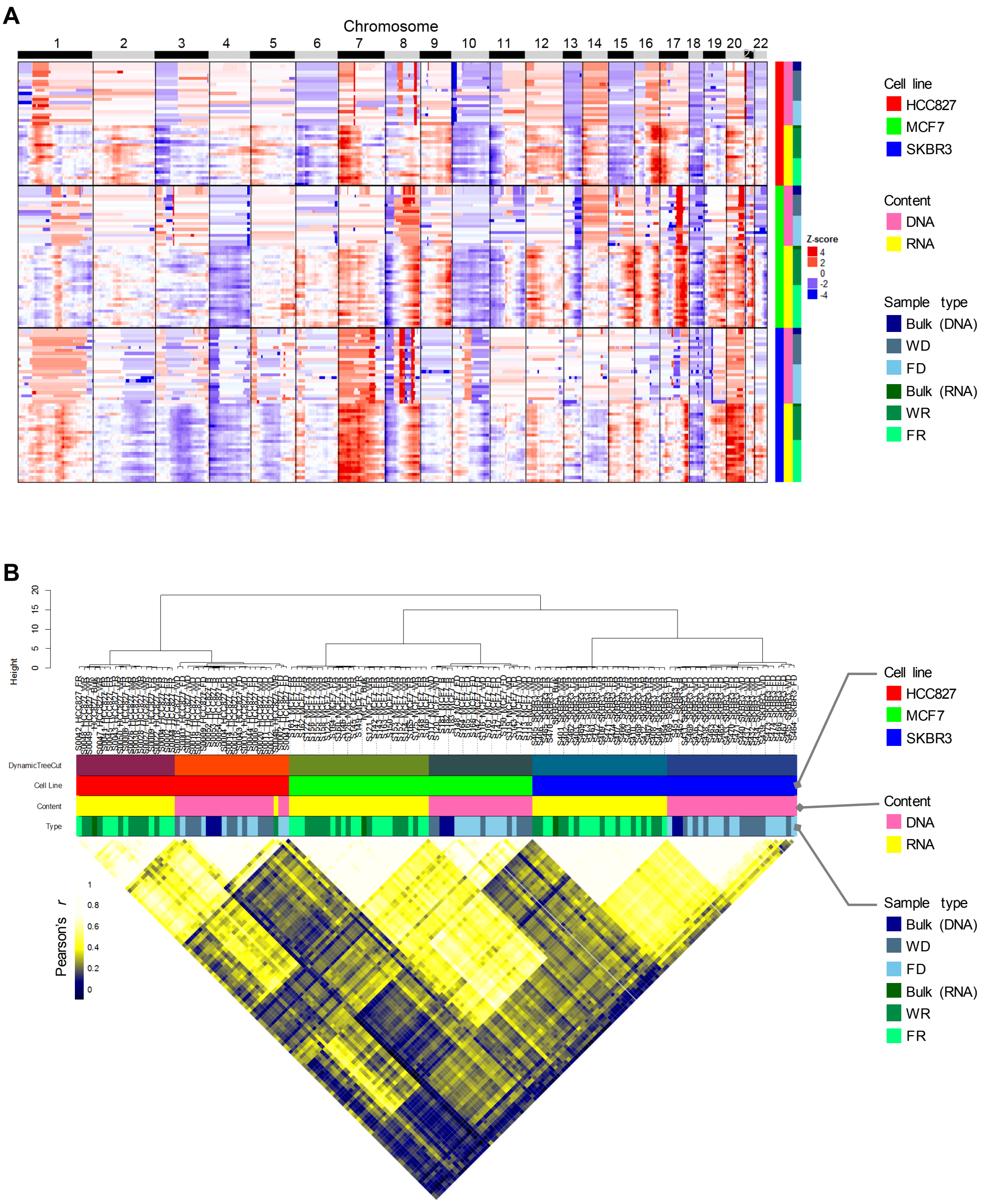

Supplemental Figure S15. Relationships between genomic copy-numbers and chromosomal expressions among HCC827, MCF7, and SKBR3 cells. (*A*) Heatmap of genomic copy-numbers and chromosomal expression across all autosomal chromosomes. (*B*) Unsupervised hierarchical clustering was performed using Ward's method and Pearson's distance over copy-number profiles estimated from genomic data and chromosomal expression data. Pairwise comparison calculated in Pearson's correlation coefficient is shown in the heatmap of the lower panel.

Supplemental Figure S16

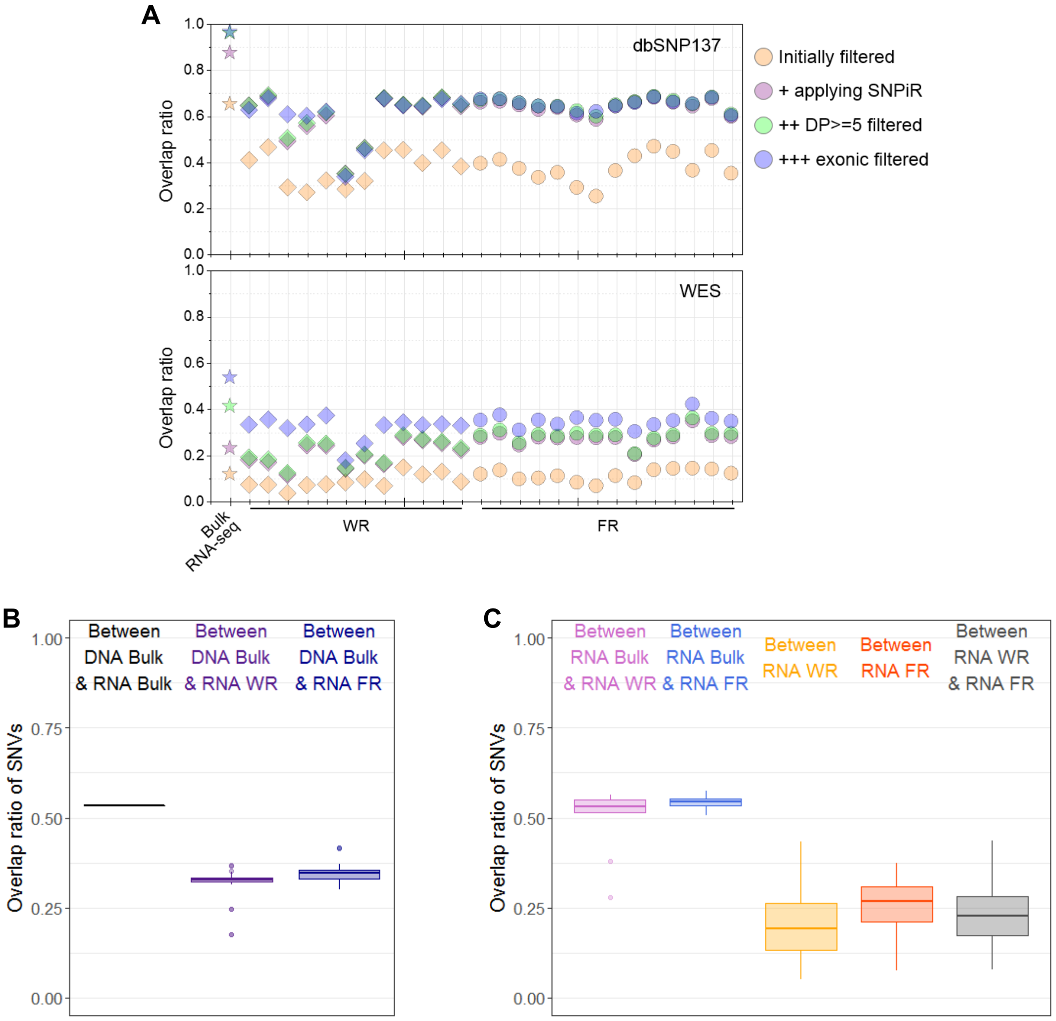

Supplemental Figure S16. Detection of variants in single-cell RNA-seq data of MCF7. (*A*) Fraction of RNA-seq variants present in dbSNP137 (upper panel) and detected in bulk WES data (lower panel). The candidate SNVs were detected in WR and FR single-cell RNA-seq data and subjected to sequential filtering steps (colored labels). Bulk cell RNA-seq data was also compared. (*B*) Boxplots displaying the fraction of RNA-seq SNVs detected in bulk cells of WES data (*C*) Fraction of WR and FR RNA-seq variants detected in bulk or single-cell RNA-seq data. (*B* and *C*) Boxes show 25th and 75th percentile with 10th and 90th percentile whiskers.

Supplemental Figure S17

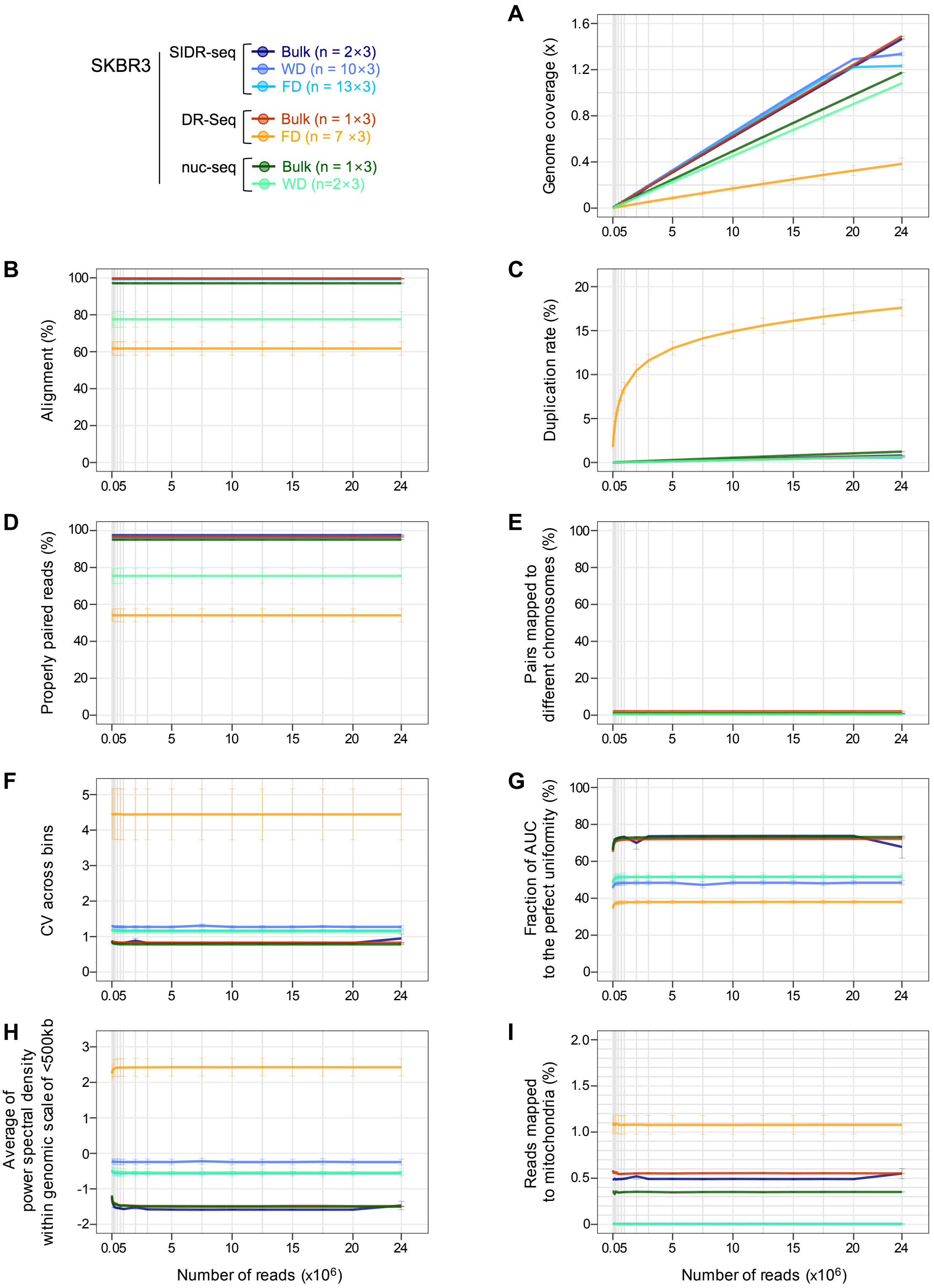

Supplemental Figure S17. Sequencing depth-dependent sensitivity and data reliability of single cell genome sequencing methods: SIDR-seq, DR-Seq, and nuc-seq. Raw reads as indicated on *x*-axis (0.05, 0.1, 0.2, 0.3, 0.5, 0.75, 1, 2, 3, 5, 7.5, 10, 12.5, 15, 17.5, 20 and 24 million) were randomly selected (in triplicate) in each sample library. (*A*) Mean genome coverage according to sequencing depth. (*B*) The rate of proper alignment (*C*) Duplication rate for the libraries. (*D*) Percentages of properly paired reads. (*E*) The percentage of reads whose paired reads mapped to different chromosomes. (*F*) Bin-to-bin variabilities in genomic DNA read counts (*G*) Comparison of coverage uniformities measured by Lorenz curves. Fractions of the area under the curve were calculated, averaged for each group, and plotted. (*H*) Power spectral densities of read distributions were obtained and averaged across frequencies greater than 1/500 kb.

Supplemental Figure S18

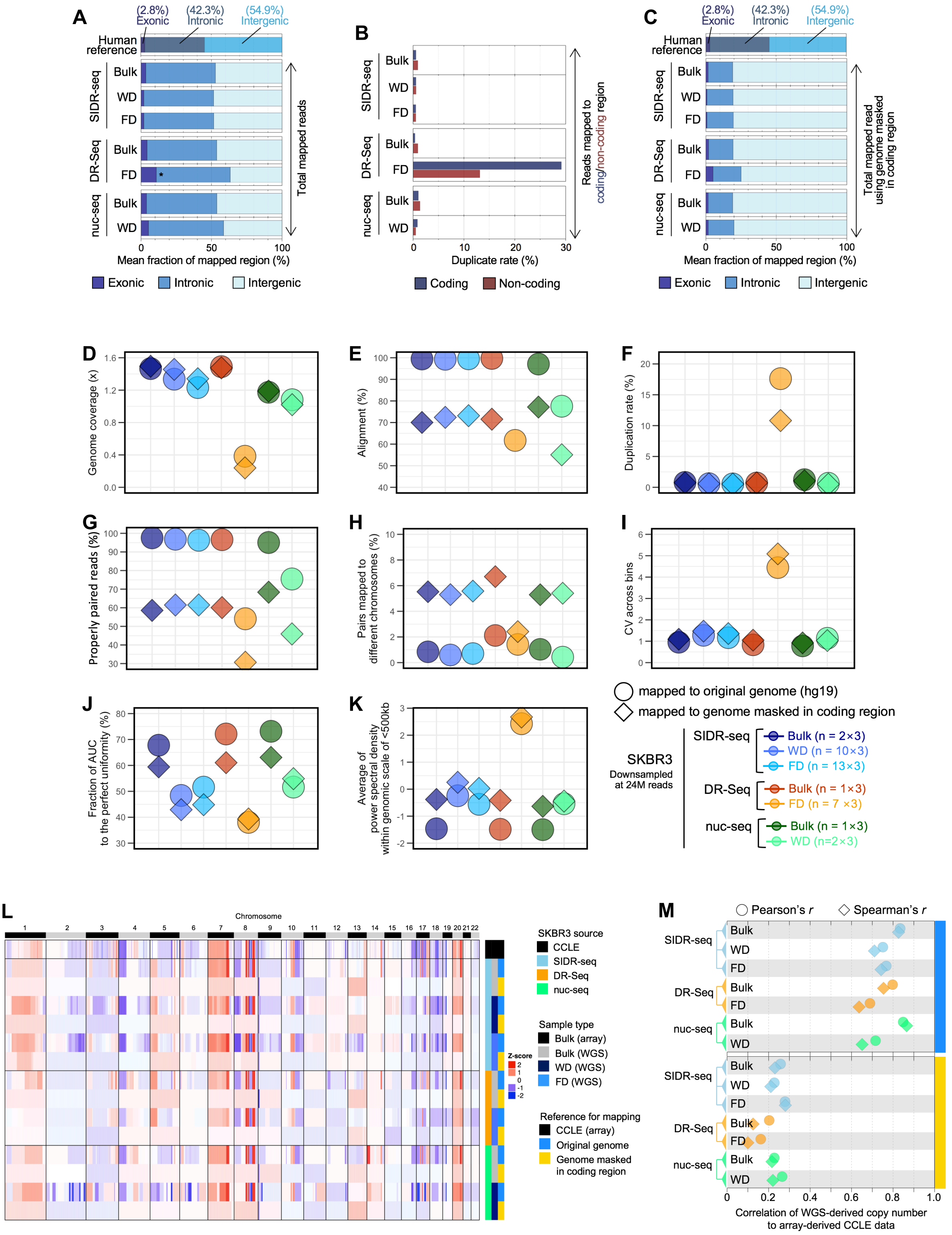

Supplemental Figure S18. Comparisons of sensitivity and data reliability among single cell genome sequencing methods using the genome reference masked in coding regions. The number of sequencing reads in each sample was set to 24 million in triplicate by randomly down-sampling from all available reads. (*A*) Fractions of genome sequencing reads mapped to exonic, intronic, and intergenic regions of the original human reference genome. Relative higher exonic fraction in the FD sample of DR-Seq was denoted as an asterisk (mean ± s.e.m; FD of DR-Seq, 10.84 ± 0.81%; Bulk of SIDR-seq, 3.36 ± 0.01%; WD of SIDR-seq, 2.00 ± 0.02%; FD of SIDR-seq, 1.96 ± 0.02%; Bulk of DR-Seq, 4.22 ± 0.001%; Bulk of nuc-seq, 3.87 ± 0.001%; WD of nuc-seq, 5.28 ± 0.12%). Top panel shows the reference fraction of human genome (Alexander et al. 2010). (*B*) Duplication rates of genome sequencing reads mapped to coding or non-coding regions. The non-coding regions include all regions other than exonic coding regions, such as intronic and intergenic regions. (*C*) Fractions of genome sequencing reads mapped to exonic, intronic, and intergenic regions in alignments to a human genome with masked coding regions. Top panel shows the reference fraction of human genome (Alexander et al. 2010). (*D-K*) Sequencing reads were mapped to either the human reference genome or the genome masked in coding regions. The analyses were performed before and after masking of the genome in coding regions. (*D-H*) The summary of sequencing metrics. (*D*) Genome sequencing depth of coverage. Plots display fractions of sequencing reads (*E*) properly aligned to the reference genome, (*F*) duplicated, (*G*) properly paired, (*H*) with their paired reads mapped to different chromosomes. (*I*) Bin-to-bin variabilities in genomic DNA read counts (*J*) Comparison of coverage uniformities measured by Lorenz curves. The fractions of the area under the curve were calculated and averaged for each group. (*K*) Comparison of coverage uniformities measured by power spectral analysis. Power spectral densities of read distributions were obtained and averaged across frequencies greater than 1/500 kb. (*L*) Heatmap of genome-wide copy-number profiles in bulk and single cells from SKBR3 cells by binning of 1 Mb genomic scale. Copy-number profiles from genome sequencing were compared to the CCLE data profiled using SNP array (at the top of the heatmap). (*M*) Correlation of copy-numbers between data sets from each method and CCLE data set. Pearson's and Spearman's correlation coefficients were plotted against x-axis. Upper panel (blue), using the original reference; lower panel (yellow), using the genome reference masked in coding region.

Supplemental Figure S19

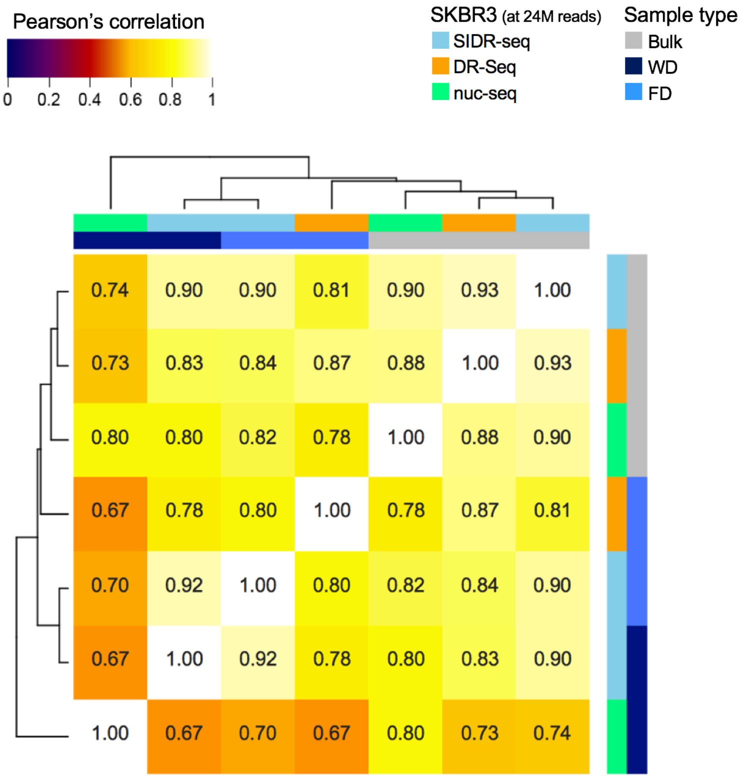

Supplemental Figure S19. Pairwise correlation of copy-number profiles among WGS SKBR3 data across SIDR-seq, DR-Seq, and nuc-seq. The hierarchical clustering tree was generated using Pearson's correlation coefficients of copy-numbers with Ward's method. The color scale indicates the degree of correlation.

Supplemental Figure S20

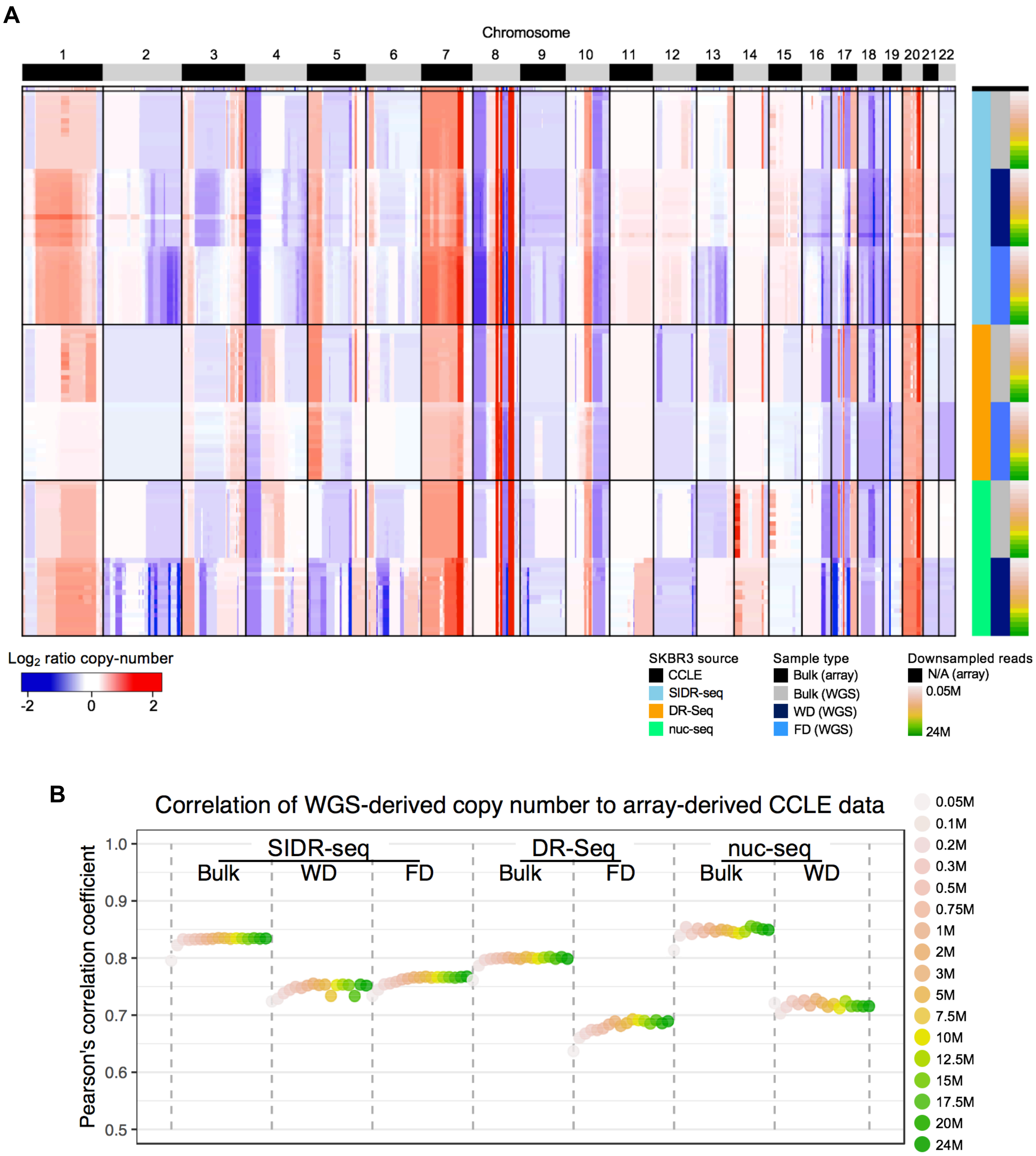

Supplemental Figure S20. Comparisons of copy-numbers between array-derived CCLE data and samples from SIDR-seq, DR-Seq, and nuc-seq. Sequencing reads were down-sampled to total read counts in a range from 0.05 to 24 M. (*A*) Heatmap of genome-wide copy-number profiles in bulk and single cells from SKBR3 cells with various read depths. Copy-number profiles were compared to the CCLE data. (*B*) The effect of sequencing depth on copy-number profiling. The depth of WGS data were varied from 0.05 to 24 million reads by *in silico* down-sampling. Pearson's correlation coefficients of copy-numbers between the DNA-seq data and CCLE data were calculated.

Supplemental Figure S21

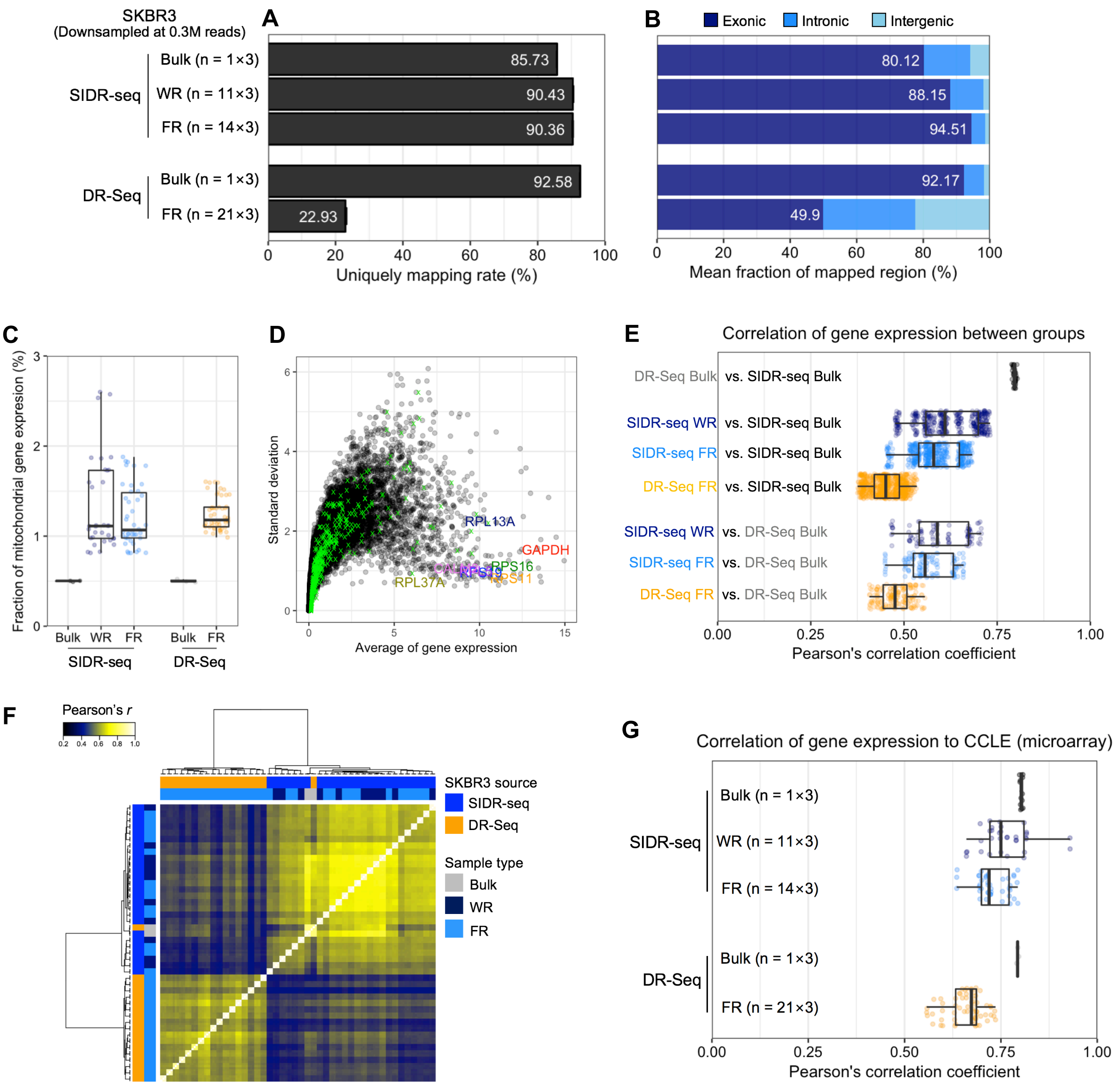

Supplemental Figure S21. Comparisons of quality of RNA-seq data between SIDR-seq and DR-Seq. Single cells from DR-Seq data (SRR1639638) were sorted according to the cell-specific barcodes, and subjected to the following analysis when their number of reads were at least higher than 0.1 M (n = 21). Sequencing reads of all SKBR3 RNA-seq samples were down-sampled to total read counts at 0.3 M in triplicate. (*A*) Percentages of uniquely mapped reads to the reference genome. (*B*) Fractions of reads mapped to exonic, intronic, and intergenic regions of the original human reference genome. Mean fraction of reads mapped to exonic regions is indicated in numbers. (*C*) The percentage of reads mapped to mitochondrial chromosome. (*D*) Relationship between average expression level and variability across single cells and bulk samples both from SIDR-seq and DR-Seq. The eight housekeeping genes with little variability (Supplemental Fig. S6) are marked in color. Housekeeping genes are marked as green. (*E*) Global

expression correlations between single cell types and bulk samples. (*F*) Pairwise correlation of gene expression profiles among RNA-seq SKBR3 data from SIDR-seq and DR-Seq. (*G*) Correlation of gene expression profiles between data sets from each method and CCLE data (SKBR3 gene expression microarray). (*C*, *E* and *G*) Boxes show 25th and 75th percentile with 10th and 90th percentile whiskers.

Supplemental Figure S22

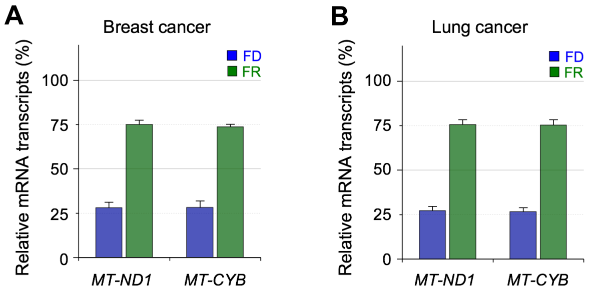

Supplemental Figure S22. Recovery rates of mitochondrial DNA by the SIDR method for tissue samples from breast cancer (*A*) and lung cancer (*B*) patients. The recovery rate of mitochondrial DNA was estimated by real time PCR targeting *MT-ND1* and *MT-CYB*. Mitochondrial DNAs were extracted from 1,000 cells of human breast cancer tissues (n=2) and human lung cancer tissues (n=3). "FD" refers to DNA fractionated by the SIDR method the amount of DNA in "FR" indicates the amount of residual contamination in the RNA fractions due to incomplete separation. The amounts of nucleic acids in each fraction were normalized to those in the whole cell lysates of 1,000 cells for each cell type. Error bars represent the s.e.m.

Supplemental Table S1. Overall QC statistics

Cell lines	Isolation method	The number of samples that passed library preparation QC		The number of samples that passed sequencing data QC		
		DNA	RNA	WGS	RNA-seq	WGS & RNA-seq
**HCC827**	Whole	10/10	10/13	10/10	10/10	NA
	Fraction	15/15	10/15	8/10	9/10	7/10
	Bulk	3/3	1/1	3/3	1/1	NA
**MCF7**	Whole	10/10	14/15	7/10	12/14	NA
	Fraction	14/14	14/14	10/14	14/14	10/14
	Bulk	3/3	1/1	3/3	1/1	NA
**SKBR3**	Whole	10/10	12/12	10/10	11/12	NA
	Fraction	14/14	14/14	13/14	14/14	13/14
	Bulk	3/3	1/1	2/2	1/1	NA
**Single-cell QC passing rate**	Whole	30/30 (100%)	36/40 (90.0%)	27/30 (90.0 %)	33/36 (91.7 %)	NA
	Fraction	43/43 (100%)	38/43 (88.4%)	31/38 (81.6%)	37/38 (97.4%)	30/38 (78.9%)
	Total	73/73 (100%)	74/83 (89.2%)	58/68 (85.3%)	70/74 (94.6%)	NA

NA: not applicable

REFERENCES

Ahdesmaki M, Fokianos K, Strimmer K. 2015. GeneCycle: Identification of Periodically Expressed Genes. R package version 1.1.2.

Alexander RP, Fang G, Rozowsky J, Snyder M, Gerstein MB. 2010. Annotating non-coding regions of the genome. *Nat Rev Genet* **11**: 559-571.

Barretina J, Caponigro G, Stransky N, Venkatesan K, Margolin AA, Kim S, Wilson CJ, Lehar J, Kryukov GV, Sonkin D et al. 2012. The Cancer Cell Line Encyclopedia enables predictive modelling of anticancer drug sensitivity. *Nature* **483**: 603-607.

Cingolani P, Platts A, Wang le L, Coon M, Nguyen T, Wang L, Land SJ, Lu X, Ruden DM. 2012. A program for annotating and predicting the effects of single nucleotide polymorphisms, SnpEff: SNPs in the genome of Drosophila melanogaster strain w1118; iso-2; iso-3. *Fly* **6**: 80-92.

DePristo MA, Banks E, Poplin R, Garimella KV, Maguire JR, Hartl C, Philippakis AA, del Angel G, Rivas MA, Hanna M et al. 2011. A framework for variation discovery and genotyping using next-generation DNA sequencing data. *Nat Genet* **43**: 491-498.

Dey SS, Kester L, Spanjaard B, Bienko M, van Oudenaarden A. 2015. Integrated genome and transcriptome sequencing of the same cell. *Nat Biotechnol* **33**: 285-289.

Harrow J, Frankish A, Gonzalez JM, Tapanari E, Diekhans M, Kokocinski F, Aken BL, Barrell D, Zadissa A, Searle S et al. 2012. GENCODE: the reference human genome annotation for The ENCODE Project. *Genome Res* **22**: 1760-1774.

Johnson WE, Li C, Rabinovic A. 2007. Adjusting batch effects in microarray expression data using empirical Bayes methods. *Biostatistics* **8**: 118-127.

Kim KT, Lee HW, Lee HO, Kim SC, Seo YJ, Chung W, Eum HH, Nam DH, Kim J, Joo KM et al. 2015. Single-cell mRNA sequencing identifies subclonal heterogeneity in anti-cancer drug responses of lung adenocarcinoma cells. *Genome Biol* **16**: 127.

Kim KT, Lee HW, Lee HO, Song HJ, Jeong DE, Shin S, Kim H, Shin Y, Nam DH, Jeong BC et al. 2016. Application of single-cell RNA sequencing in optimizing a combinatorial therapeutic strategy in metastatic renal cell carcinoma. *Genome Biol* **17**: 80.

Leek JT, Johnson WE, Parker HS, Jaffe AE, Storey JD. 2012. The sva package for removing batch effects and other unwanted variation in high-throughput experiments. *Bioinformatics* **28**: 882-883.

Li B, Dewey CN. 2011. RSEM: accurate transcript quantification from RNA-Seq data with or without a reference genome. *BMC Bioinformatics* **12**: 323.

Li H. 2013. Aligning sequence reads, clone sequences and assembly contigs with BWA-MEM. *ArXiv e-prints* **1303**.

Li H, Handsaker B, Wysoker A, Fennell T, Ruan J, Homer N, Marth G, Abecasis G, Durbin R. 2009. The Sequence Alignment/Map format and SAMtools. *Bioinformatics* **25**: 2078-2079.

Navin N, Kendall J, Troge J, Andrews P, Rodgers L, McIndoo J, Cook K, Stepansky A, Levy D, Esposito D et al. 2011. Tumour evolution inferred by single-cell sequencing. *Nature* **472**: 90-94.

Park J-M, Lee J-Y, Lee J-G, Jeong H, Oh J-M, Kim YJ, Park D, Kim MS, Lee HJ, Oh JH. 2012. Highly efficient assay of circulating tumor cells by selective sedimentation with a density gradient medium and microfiltration from whole blood. *Anal Chem* **84**: 7400-7407.

Piskol R, Ramaswami G, Li JB. 2013. Reliable identification of genomic variants from RNA-seq data. *Am J Hum Genet* **93**: 641-651.

Satija R, Farrell JA, Gennert D, Schier AF, Regev A. 2015. Spatial reconstruction of single-cell gene expression data. *Nat Biotechnol* **33**: 495-502.

Seshan V, Olshen A. 2016. DNAcopy: DNA copy number data analysis. R package version 1.48.0.

Sherry ST, Ward MH, Kholodov M, Baker J, Phan L, Smigielski EM, Sirotkin K. 2001. dbSNP: the NCBI database of genetic variation. *Nucleic Acids Res* **29**: 308-311.

The 1000 Genomes Project Consortium. 2015. A global reference for human genetic variation. *Nature* **526**: 68-74.

The International HapMap 3 Consortium. 2010. Integrating common and rare genetic variation in diverse human populations. *Nature* **467**: 52-58.

Tirosh I, Izar B, Prakadan SM, Wadsworth MH, 2nd, Treacy D, Trombetta JJ, Rotem A, Rodman C, Lian C, Murphy G et al. 2016. Dissecting the multicellular ecosystem of metastatic melanoma by single-cell RNA-seq. *Science* **352**: 189-196.

Wang Y, Waters J, Leung ML, Unruh A, Roh W, Shi X, Chen K, Scheet P, Vattathil S, Liang H. 2014. Clonal evolution in breast cancer revealed by single nucleus genome sequencing. *Nature* **512**: 155-160.

Whitfield ML, Sherlock G, Saldanha AJ, Murray JI, Ball CA, Alexander KE, Matese JC, Perou CM, Hurt MM, Brown PO. 2002. Identification of genes periodically expressed in the human cell cycle and their expression in tumors. *Mol Biol Cell* **13**: 1977-2000.

Zong C, Lu S, Chapman AR, Xie XS. 2012. Genome-wide detection of single-nucleotide and copy-number variations of a single human cell. *Science* **338**: 1622-1626.
